# Large Language Models as a Consulting Hotline for Patients With Breast Cancer and Specialists in China: Cross-Sectional Questionnaire Study

**DOI:** 10.2196/66429

**Published:** 2025-05-27

**Authors:** Hui Liu, Jialun Peng, Lu Li, Ao Deng, XiangXin Huang, Guobing Yin, Haojun Luo

**Affiliations:** 1 Department of Thyroid and Breast Surgery, The Second Affiliated Hospital of Chongqing Medical University, 74 Linjiang Road, Chongqing, 400010, China, 86 13452999485; 2 Department of Hepatobiliary Surgery, The Second Affiliated Hospital of Chongqing Medical University, Chongqing, China; 3 Department of Thyroid and Breast Surgery, Renji Hospital Affiliated of Chongqing University, Chongqing, China

**Keywords:** large language models, breast cancer, health education, cross-sectional study

## Abstract

**Background:**

The disease burden of breast cancer is increasing in China. Guiding people to obtain accurate information on breast cancer and improving the public’s health literacy are crucial for the early detection and timely treatment of breast cancer. Large language model (LLM) is a currently popular source of health information. However, the accuracy and practicality of the breast cancer–related information provided by LLMs have not yet been evaluated.

**Objective:**

This study aims to evaluate and compare the accuracy, practicality, and generalization-specificity of responses to breast cancer–related questions from two LLMs, ChatGPT and ERNIE Bot (EB).

**Methods:**

The questions asked to the LLMs consisted of a patient questionnaire and an expert questionnaire, each containing 15 questions. ChatGPT was queried in both Chinese and English, recorded as ChatGPT-Chinese (ChatGPT-C) and ChatGPT-English (ChatGPT-E) respectively, while EB was queried in Chinese. The accuracy, practicality, and generalization-specificity of each inquiry’s responses were rated by a breast cancer multidisciplinary treatment team using Likert scales.

**Results:**

Overall, for both the patient and expert questionnaire, the accuracy and practicality of responses from ChatGPT-E were significantly higher than those from ChatGPT-C and EB (all *Ps*<.001). However, the responses from all LLMs are relatively generalized, leading to lower accuracy and practicality for the expert questionnaire compared to the patient questionnaire. Additionally, there were issues such as the lack of supporting evidence and potential ethical risks in the responses of LLMs.

**Conclusions:**

Currently, compared to other LLMs, ChatGPT-E has demonstrated greater potential for application in educating Chinese patients with breast cancer, and may serve as an effective tool for them to obtain health information. However, for breast cancer specialists, these LLMs are not yet suitable for assisting in clinical diagnosis or treatment activities. Additionally, data security, ethical, and legal risks associated with using LLMs in clinical practice cannot be ignored. In the future, further research is needed to determine the true efficacy of LLMs in clinical scenarios related to breast cancer in China.

## Introduction

Breast cancer has become the most common malignant tumor globally, with an estimated 11.7% of all new cancer cases in 2020 [1]. The incidence of breast cancer has been rising in China, with 420,000 Chinese women diagnosed in 2020, accounting for 18% of global cases [2]. Breast cancer also contributes significantly to cancer-related deaths; however, early detection and timely treatment play a significant role in reducing its mortality rate [3-5]. Providing health education through appropriate channels and disseminating accurate medical health information to the public can help improve public awareness of breast cancer, thereby alleviating the burden of breast cancer in China. Currently, the internet is the primary source for people to obtain health information. Recent studies showed that 55% of Europeans aged 16‐74 years seek health-related information online, while in mainland China, nearly 79% of the population searches for health information on the internet [6,7]. However, the quality of online health information varies considerably, and inaccurate or even erroneous health information may lead to patients making inappropriate medical decisions, posing a threat to public health [8-10]. Large language model (LLM) is a type of chatbot that combines artificial intelligence with natural language processing, are trained on massive text data [11]. ChatGPT, developed by OpenAI, has garnered global attention since its release and been applied across multiple fields. ERNIE Bot (EB; Chinese name: Wenxin-Yiyan), developed by Baidu, benefits from Baidu’s strong influence in artificial intelligence and has achieved significant popularity and a user base in the Chinese market. These tools have recently become widely popular and demonstrated significant potential in the medical field [12]. Studies have shown that ChatGPT has greater potential for patient education in breast reconstruction and diabetes self-management, while also being able to accurately answer some cancer-related questions [11,13,14]. However, some scholars have questioned the accuracy and practicality of the medical health information provided by ChatGPT [12]. Currently, there is a lack of studies evaluating the educational potential of ChatGPT and EB—two of the most commonly used LLMs in China—among Chinese patients with breast cancer and their utility for Chinese breast cancer physicians. To address this gap, this study assesses whether these LLMs can serve as educational tools for Chinese patients with breast cancer and clinical assistance tools for Chinese breast cancer specialists by comparing the accuracy and reliability of responses to breast cancer–related questions between ChatGPT and EB.

## Methods

### Questionnaire Design and Data Collection

The questions asked to LLMs consisted of a patient questionnaire and an expert questionnaire, each containing 15 questions and covering aspects such as the diagnosis, treatment, prognosis, and follow-up of breast cancer. The patient questionnaire was derived by distributing a questionnaire to patients with breast cancer to investigate their most important concerns (Textbox 1). The expert questionnaire was summarized by two experienced breast surgeons, based on the National Comprehensive Cancer Network (NCCN) Clinical Practice Guidelines in Oncology for breast cancer and the International Consensus Guidelines for advanced breast cancer (Textbox 2) [15,16]. On January 15, 2024, all questions were input into ChatGPT (version 4.0) and EB (version 4.0). Each input was independently entered using the “new chat” function and inputted twice to detect its repeatability. To optimize the responses of the LLMs, prompt engineering was applied with the same lead-in statement: “Now that you are a breast cancer specialist, please answer the following questions,” which was input into the LLMs along with each question. As ChatGPT was developed in the United States, we queried ChatGPT in both Chinese and English, denoted as ChatGPT-Chinese (ChatGPT-C) and ChatGPT-English (ChatGPT-E), respectively, and the EB, developed in China, was queried in Chinese only. The responses from ChatGPT and EB were recorded using Microsoft Excel (Multimedia Appendix 1).

Textbox 1. **Specific contents of patient questionnaire.**

**Patient Questionnaire**

1. Is breast cancer hereditary, and will it have an impact on my descendants?
2. What impact does the staging of breast cancer have on treatment and prognosis?
3. What are the treatment methods for breast cancer, and which one should I choose?
4. What are the various surgical treatment methods for breast cancer, and how do they each impact the appearance of the breast?
5. What is the total cost of treating breast cancer in China?
6. What aspects are included in the postoperative rehabilitation training for breast cancer, and what benefits does it bring to rehabilitation
7. Is breastfeeding possible during breast cancer treatment?
8. Why do I need systemic treatment (such as chemotherapy, endocrine therapy, or targeted therapy) after breast cancer surgery?
9. What are the adverse reactions of drugs used in breast cancer treatment?
10. How do I manage psychological and emotional health issues during the treatment of breast cancer?
11. What lifestyles contribute to the recovery of breast cancer patients?
12. What daily care is required for a subcutaneously implanted infusion port?
13. Can breast cancer patients have normal fertility after discharge?
14. What is the risk of recurrence and the corresponding monitoring methods after breast cancer treatment?
15. If a breast cancer patient has other chronic illnesses or new health issues that need to be addressed, how should these issues be coordinated with the treatment of breast cancer?

Textbox 2. **Specific contents of expert questionnaire.**

**Expert Questionnaire**

1. What are the screening methods for breast cancer?
2. What imaging and biomarkers will you use to assist in the preoperative diagnosis of breast cancer?
3. What are the requirements for the surgical margins in breast-conserving surgery for ductal carcinoma in situ?
4. For cN1 breast cancer patients who have converted to cN0 after neoadjuvant therapy, what are the requirements for sentinel lymph node biopsy at this stage?
5. What surgical methods do you know for stage I breast reconstruction?
6. What is the strategy for adjuvant chemotherapy in early-stage triple-negative breast cancer?
7. For early-stage high-risk breast cancer patients with strongly positive hormone receptors, which adjuvant endocrine therapy would you recommend?
8. What are the different classes of drugs for anti-HER2 therapy?
9. What is the first-line treatment of choice for stage IV or recurrent metastatic HR-positive/HER2-negative breast cancer?
10. What are the conditions for exemption from radiotherapy after breast-conserving surgery
11. What are the common regimens for neoadjuvant therapy in triple-negative breast cancer?
12. What are your basic principles for the treatment of metastatic breast cancer?
13. What are your recommendations for the management of bone health in patients during adjuvant endocrine therapy?
14. For young female breast cancer patients with HR-positive tumors who express a desire for fertility, what considerations do you have in the treatment plan?
15. How should long-term follow-up and monitoring be conducted for breast cancer patients?

### Response Assessment

The breast cancer multidisciplinary treatment team scored the accuracy, practicality, and generalization-specificity of each response using a Likert scale, with the poorer of the two responses being included in the final score if the responses were inconsistent. The team consisted of 13 members, including 7 breast cancer specialists, 2 imaging specialists, 2 pathology specialists, and 2 nursing specialists. The Likert scale is a hierarchical scale, originally developed by Likert and has been used extensively in several research studies [17,18]. Accuracy was divided into 6 levels from 1 to 6, with higher scores indicating better accuracy (Table 1). Practicality was divided into 4 levels from 1 to 4, with higher scores indicating better practicality (Table 2). The generalization-specificity score (GSS) is divided into 5 levels from 1 to 5, with higher scores indicating better specificity (Table 3). To reduce bias caused by individual differences in understanding the scoring system, all experts reviewed and discussed the scoring criteria of the Likert scale before the assessment.

**Table 1.** Accuracy scoring standard.

Scoring description	Scoring
Completely incorrect	1
More incorrect than correct	2
Approximately equal correct and incorrect	3
More correct than incorrect	4
Nearly all correct	5
All correct	6

**Table 2.** Practical scoring standard.

Scoring description	Scoring
Completely impractical	1
Slightly practical	2
Moderately practical	3
Very practical	4

**Table 3.** Generalization-Specificity Score (GSS) scoring standard.

Scoring description	Scoring
Fully generalized, with no specific details or targeted information provided	1
Primarily generalized but mentions some relevant details or information	2
Combines generalized content with some specific details or information	3
Rather specific, but the details or targeted information are insufficient and can be improved	4
Fully specific, with comprehensive details and highly targeted information	5

### Statistical Analysis

The Shapiro-Wilk test was used to determine the normality of the scores and the Levene test was used to evaluate the homogeneity of variance. Differences between two groups were assessed using the Wilcoxon rank sum test. The Kruskal-Wallis test evaluated differences between three or more groups of variables, and the Dunn test was used for two-way between-group comparisons of variables that were not normally distributed. *P*<.05 was deemed statistically significant. The intraclass correlation coefficient (ICC) was used to evaluate the consistency of accuracy, practicality scores and GSS among 13 raters. An ICC ≥0.75 was considered to indicate good consistency. All statistical analyses were performed using R software (version 4.0.3; R Foundation for Statistical Computing).

### Ethical Considerations

This study did not gather patient data and did not involve human subjects. Therefore, approval by the institutional review board of Chongqing Medical University was not required.

## Results

**Figure 1.**
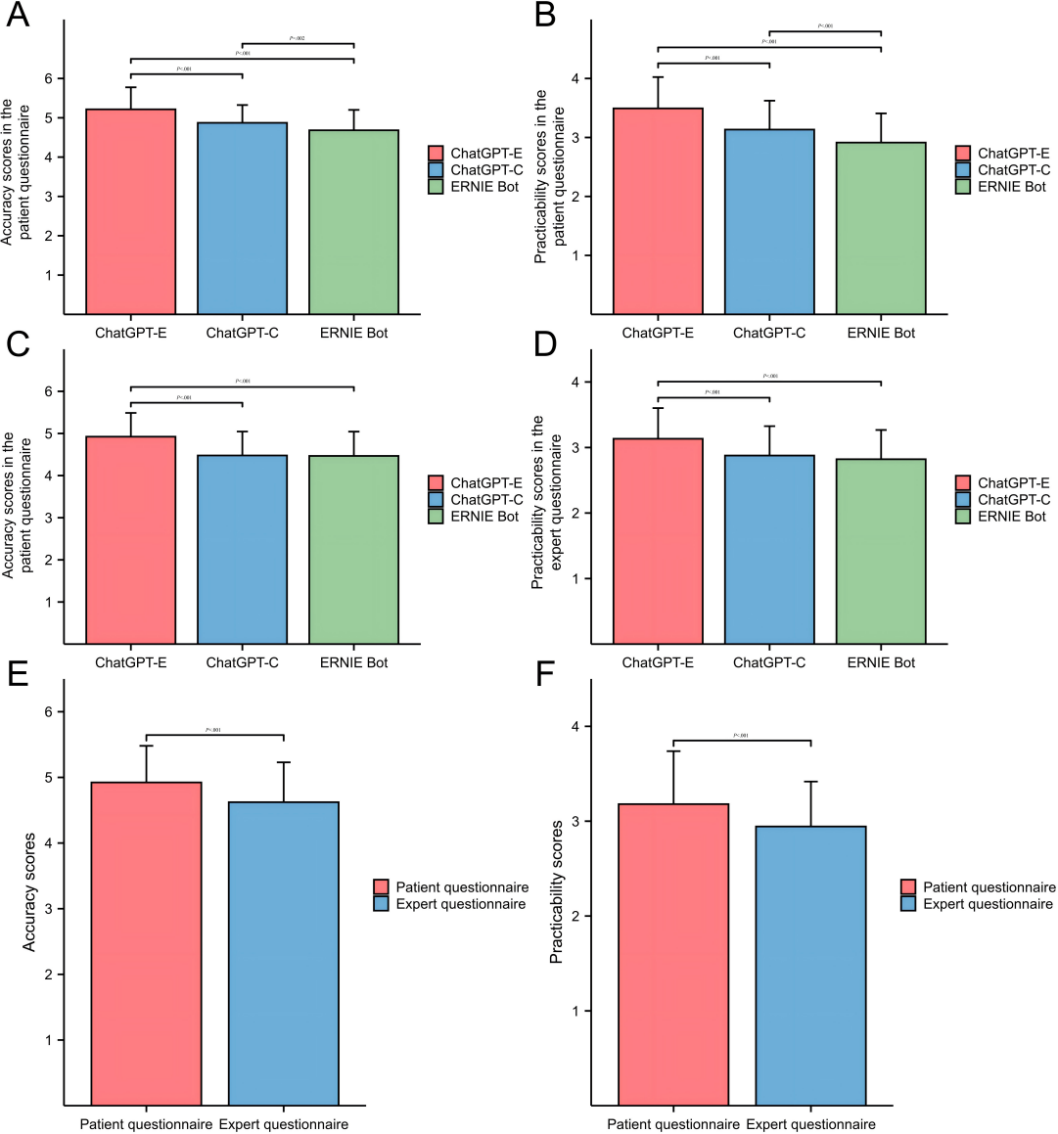
Scores and comparisons of the overall accuracy and practicality of the LLMs' responses. (A,B): Patient questionnaire; (C,D) Expert questionnaire; (E,F): Comparison of patient and expert questionnaire. Error bars represent mean ± standard error.

In the patient questionnaire, the median accuracy scores of ChatGPT-E, ChatGPT-C, and EB were 5.00 (IQR 5.00-6.00), 5.00 (IQR 5.00-6.00), and 5.00 (IQR 4.00-5.00), respectively. The median practicality scores of ChatGPT-E, ChatGPT-C, and EB were 4.00 (IQR 3.00-4.00), 3 (IQR 3.00-3.00), and 3.00 (IQR 3.00-3.00), respectively; and the median GSS were 4.00 (IQR 3.00-4.00), 3 (IQR 3.00-4.00), and 3.00 (IQR 3.00-4.00), respectively. The accuracy and practicality of ChatGPT-E were significantly higher than those of ChatGPT-C and EB (*P*<.001). The accuracy and practicality of ChatGPT-C were also significantly higher than those of EB (*P*=.002 and *P*<.001, respectively; Figure 1A and B). The specificity of the ChatGPT-E responses was significantly higher than that of the ChatGPT-C and EB (*P*=.002 and *P*<.001, respectively), whereas no significant difference was found in the specificity of the ChatGPT-C and EB responses (Multimedia Appendix 2, parts A and B).

In the expert questionnaire, the median accuracy scores of ChatGPT-E, ChatGPT-C, and EB were 5.00 (IQR 5.00-5.00), 4.00 (IQR 4.00-5.00), and 4.00 (IQR 4.00-5.00), respectively; the median practicality scores for all three were 3.00 (IQR 3.00-3.00) and the median GSS were 4.00 (IQR 3.00-4.00), 3 (IQR 2.00- 4.00), and 3.00 (IQR 2.00-3.00), respectively. The accuracy and practicality of ChatGPT-E were significantly higher than that of ChatGPT-C and EB (all *P*<.001). However, there was no significant difference in the accuracy and practicality scores between ChatGPT-C and EB (*P*=1.000 and *P*=.72, respectively) (Figure 1). For response generalization and specificity, the ChatGPT-E score was significantly higher than ChatGPT-C and EB (both *P*<.001), whereas there was no significant difference between ChatGPT-C and EB. Overall, the median accuracy scores of the patient questionnaire and the expert questionnaire were 5.00 (IQR 5.00-5.00) and 5.00 (IQR 4.00-5.00), respectively; the median practicality scores were 3.00 (IQR 3.00-4.00), and 3.00 (IQR 3.00-3.00), respectively; the median practicality scores were 3.00 (IQR 3.00-4.00), and 3.00 (IQR 3.00-3.00), respectively, and the median GSS was 3.00 (IQR 3.00-4.00) for both questionnaires. The accuracy and practicality scores from the patient questionnaire were significantly higher than those from the expert questionnaire (all *P*<.001) (Figure 1), and their specificity scores were also significantly higher (*P*<.001) (Multimedia Appendix 2, parts C-E).

In addition, to quantify the frequency of ratings for accuracy, practicality, and specificity in LLM responses, we illustrated the rating distribution as percentages in Multimedia Appendix 3. For accuracy, responses rated as 6 (All correct) accounted for only 11.96% (70/585) in the patient questionnaire, 5.64% (33/585) in the expert questionnaire, and 8.8% (103/1170) overall. Notably, the hallucination rate—defined as responses rated ≤4 on the accuracy scale—was 19.7% (115/585) in the patient questionnaire and 28.9% (169/585) in the expert questionnaire. For practicality, responses rated as 4 (Very practical) accounted for 26.15% (153/585) in the patient questionnaire, 8.55% (50/585) in the expert questionnaire, and 17.35% (203/1170) overall. For generalization-specificity, responses rated as 5 (Fully specific) accounted for 5.64% (33/585) in the patient questionnaire, 2.39% (14/585) in the expert questionnaire, and 4.02% (47/1170) overall. The 13 raters exhibited excellent inter-rater agreement in their scoring of accuracy, practicality, and GSS, with ICC values of 0.878 (95%Cl 0.837‐0.912), 0.823 (95%Cl 0.765‐0.873), and 0.809 (95%Cl 0.758‐0.855) respectively. Additionally, the statistical descriptive indices for all between-group comparisons are provided in detail in MultimediaAppendices 4^8^.

## Discussion

### Principal Findings

**Figure 2.**
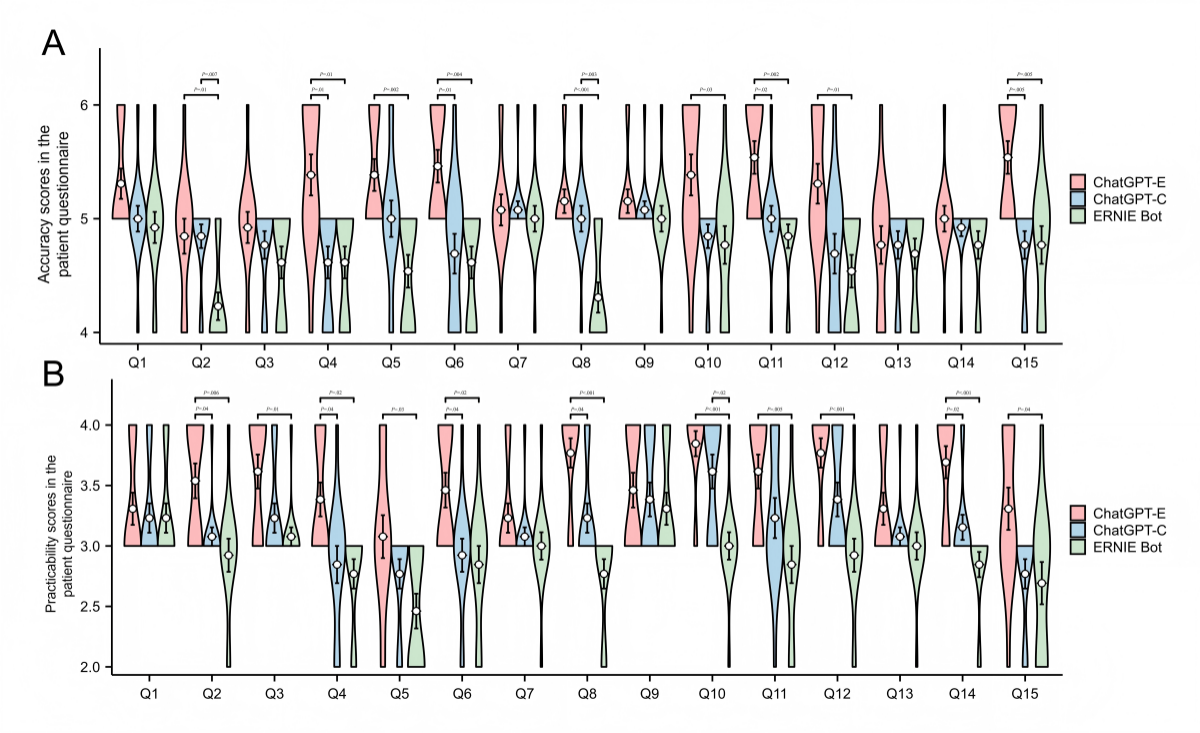
Scores and comparisons of LLMs' responses to specific questions in the patient questionnaire. A: Accuracy; B: Practicality. Error bars represent mean ± standard error. ChatGPT-E: ChatGPT-English ; ChatGPT-C: ChatGPT-Chinese; EB: ERNIE Bot.

**Figure 3.**
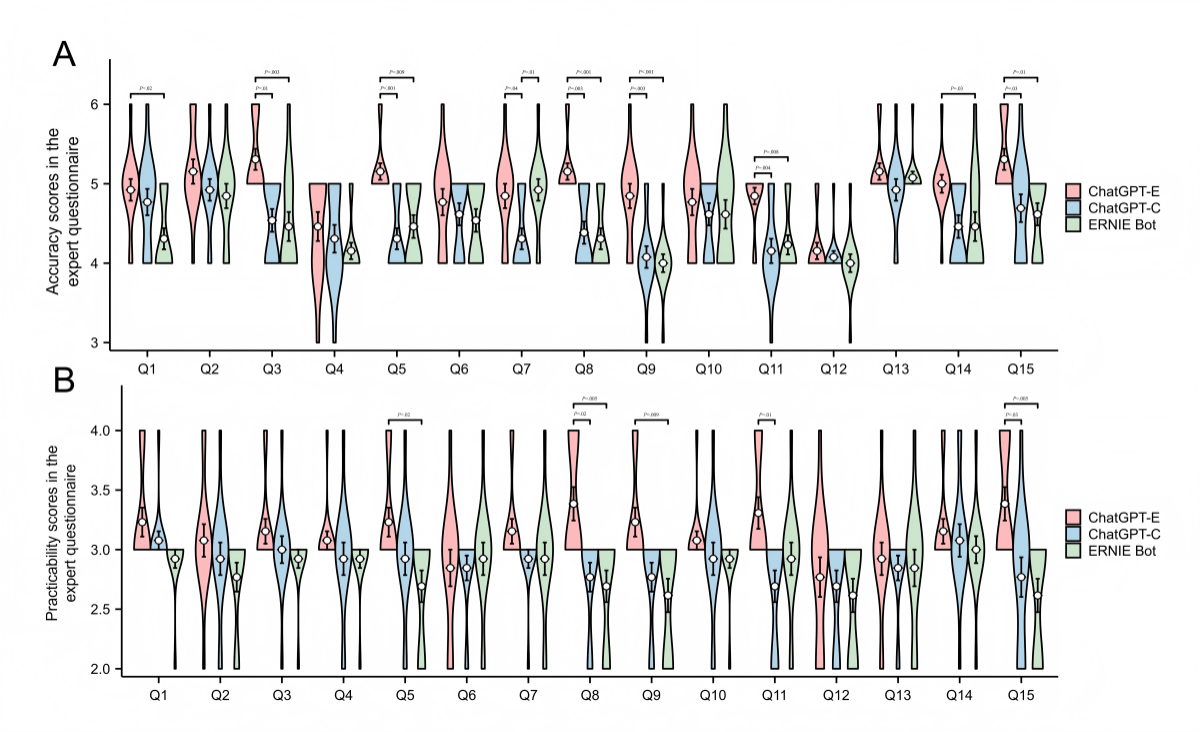
Scores and comparisons of LLMs' responses to specific questions in the expert questionnaire. A: Accuracy; B: Practicality. Error bars represent mean ± standard error. ChatGPT-E: ChatGPT-English ; ChatGPT-C: ChatGPT-Chinese; EB: ERNIE Bot.

We have reported several important findings in this study. First, based on the patient questionnaire responses, ChatGPT-E demonstrated significantly higher accuracy compared to ChatGPT-C and EB in addressing questions related to breast cancer surgery treatment (Q4) and postoperative management (Q6, Q11, and Q15) (Figure 2A). Additionally, ChatGPT-E’s responses to questions concerning breast cancer staging (Q2), treatment (Q4 and Q8), and postoperative management (Q6 and 14) were more comprehensive and practical (Figure 2). In the expert questionnaire, ChatGPT-E demonstrated similar advantages, especially for breast cancer drug treatment (Q8) and follow-up (Q15), with more comprehensive, accurate, and practical responses, reflecting higher efficiency (Figure 3). Overall, ChatGPT-E performed the best in both patient and expert questionnaires. Despite the advantages in training strategies that may have enabled ChatGPT-C to perform better than EB in answering general questions from patients with breast cancer, the performances of both models were unsatisfactory while answering comparatively specialized questions in the field of breast cancer in the Chinese-language context (Figure 1). For example, in response to the expert questionnaire Q5, both ChatGPT-C and EB only briefly mentioned several common methods of first-stage breast reconstruction. Only ChatGPT-E mentioned “latissimus dorsi flap breast reconstruction” and briefly introduced the advantages of each surgical method. Although its response was not detailed enough, the basic framework was correct. Although the overall hallucination rate of ChatGPT-E was significantly lower than that of ChatGPT-C and EB (both *P*<.001), 11.79% (23/195) of its responses in the expert questionnaire still contained inaccurate information. This finding indicates that even models with relatively superior performance must further reduce hallucination rates in the specialized field of breast cancer to meet clinical requirements.

### Cross-Language Limitations of ChatGPT

In addition, we found that ChatGPT-C’s responses to the 15 questions in the patient and expert questionnaire each contained one notable medical terminology translation error. For instance, “泛素酮 (Tamoxifen)” and “莱特罗唑 (Letrozole)” were mentioned in the response to expert questionnaire Q7. In patient questionnaire Q12, the term “port” in “subcutaneously implanted infusion port” was translated as “harbor” (ie, 港口 in Chinese). Patients may misinterpret postoperative care requirements due to this nonstandard translation, potentially leading to complications. In the medical domain, English is the primary language for international academic communication. ChatGPT’s core training data is predominantly in English, including extensive English medical literature and clinical guidelines (eg, National Comprehensive Cancer Network and Advanced Breast Cancer). Compared with EB, ChatGPT has greater access to and understanding of these professional resources, enabling it to provide more comprehensive and accurate information when answering related questions. The inferior performance of ChatGPT-C compared to ChatGPT-E may stem from limitations in cross-language processing. Although ChatGPT supports multiple languages, the semantic structure and medical terminology in Chinese differ significantly from English. During cross-language processing, ChatGPT may rely on translation mechanisms rather than native Chinese training, leading to semantic distortion in specialized content and reduced answer quality. Preliminary testing in other languages such as Spanish and French has identified similar issues when dealing with proper nouns (eg, medications, surgical procedures) and compound terms. The model tends to rely on literal translation or the creation of neologisms rather than following localized standards, which may lead to ambiguities. However, a recent study by Tian et al [19] on CHIMED-GPT found that pretraining GPT using a specific Chinese medical dataset made CHIMED-GPT perform significantly better than other models in tasks such as multiple choice and open-ended responses. To address the translation errors in ChatGPT-C’s responses to breast cancer–related questions, fine-tuning the model with Chinese medical datasets represents an effective improvement strategy. These datasets should include a wide range of Chinese medical literature, clinical guidelines, case reports, and patient-doctor dialogues specific to the field of breast cancer. By training the model on these specialized datasets would help it grasp the nuances and context of medical language better, leading to more accurate translations and responses. In addition, user error correction interfaces can be designed to allow physicians or patients to flag translation errors (eg, “莱特罗唑 → 来曲唑"), and the system could then automatically collect these error cases and add them into fine-tuning datasets, thus achieving continuous model optimization. In terms of response repeatability, the performance of ChatGPT-E (52/60, 86.67%) was significantly better than ChatGPT-C (50/60, 83.33%) and EB (40/60, 66.67%).

### Challenges Encountered by LLMs

It is worth noting that we have also found a lack of corresponding empirical data and references to support the views of the two LLMs in their responses, which could undermine the credibility and practicality of their responses, especially in evidence-based clinical practice [20,21]. For example, in responses to the expert questionnaire Q14 and patient questionnaire Q7, both LLMs mentioned that “chemotherapy affects fertility or breastfeeding,” but failed to provide any useful references, resulting in compromised credibility and possibly inability to guide physicians and patients in making correct decisions. In addition, the responses of LLMs were relatively generalized, indicating that they were widely mentioned but lacked specificity, similar to the findings of the study by Giannakopoulos et al [22] who used LLMs to answer dental-related questions. This generalized responses also resulted in the LLMs being less accurate and practical in answering the expert questionnaire than the patient questionnaire (Figure 1). For example, in the responses to expert questionnaire Q15 and patient questionnaire Q14, although LLMs mentioned the need for regular follow-up and corresponding examinations for patients with breast cancer, they did not provide specific answers. These generalized responses are of limited value to clinical professionals, who require highly accurate, comprehensive, and professional information, similar to previous studies on ChatGPT’s responses to mental health and liver cancer–related questions [23,24]. However, they may be beneficial for patients with breast cancer who lack medical expertise, as the responses from LLMs already covered the vast majority of the questions and were comparable to clinician responses, similar to the findings from a study by Endo et al [25] on the use of LLMs for answering questions related to liver transplantation. Generalized LLM–generated information poses risks ranging from clinical mismanagement to ethical violations, particularly in complex fields such as breast cancer. Given that in breast cancer treatment, timely decision-making is critical early diagnosis and intervention significantly improve cure and survival rates. Vague recommendations from LLMs may put patients at risk of missing the optimal therapeutic window, thus potentially exacerbating disease progression. While LLMs such as ChatGPT-E show promise in patient education, their utility depends on the patients’ ability to contextualize and validate the outputs provided. Patients should maintain a cautious attitude toward responses generated by LLMs that lack personalized recommendations and refrain from relying on them exclusively. It is recommended that patients use the information provided by LLMs as a general reference, while promptly communicating with professional physicians. By integrating their specific clinical circumstances, patients can obtain accurate and personalized medical advice and guidance to safeguard their health and safety.

### Ethics and Data Security in LLMs

Furthermore, LLMs have exposed potential ethical risks in responding to breast cancer–related questions. While clinical trials may offer access to the latest therapeutic regimens and advanced technologies, they inherently carry uncertainties and potential adverse effects. When responding to Patient Questionnaire Q2 and Expert Questionnaire Q6, LLMs encouraged patient participation in clinical trials without adequately explaining the risks and uncertainties involved. This could lead patients to assume unnecessary risks without being fully informed [26]. We recommend establishing a dedicated review team to systematically audit medical recommendations provided by LLMs, particularly regarding clinical trial recommendations and vague suggestions. This ensures that LLMs responses adhere to medical ethical standards and professional norms, and correct or block responses that do not meet the requirements. At the same time, the issue of data security involved in LLMs is becoming increasingly prominent [27]. Although the responses of the LLMs in this study did not inadvertently leak sensitive information and were based on general medical knowledge and standardized recommendations, some studies have shown that LLMs may inadvertently memorize and disclose original data in their responses [28,29]. In a study by Nasr et al [30], researchers were able to extract training data for various LLMs including ChatGPT through specific “attacks”. Therefore, doctors or health care organizations should obtain informed consent from patients when using real patient data for model training and application to LLMs, while ensuring the anonymization and deidentification of data [26]. Patients should also be trained in data security awareness and instructed to avoid disclosing private personal information while using LLMs to obtain health information [31]. China has established a series of laws and regulations, including the Personal Information Protection Law (PIPL) to regulate data processing and applications. However, specific regulatory details for LLM–based medical applications are still being explored and refined. The effective implementation of these laws and regulations in the LLM–based medical applications is currently a concern [32]. Based on the results of this study, future regulation should focus on standardizing the LLM–based medical applications. It is essential to strictly regulate data processing procedures to ensure data security and privacy protection in compliance with laws and regulations such as the PIPL. Further, an ethical review mechanism for LLM–based medical applications should be established, clarifying ethical guidelines in aspects such as medical information provision and patient decision-making guidance to prevent ethical risks [33]. Additionally, explicit limitations should be imposed on the scope and modalities of PIPLs in health care to prevent their excessive involvement in core medical operations when sufficient reliability is not assured.

### Conclusion

This study reflects some important issues that may arise when using LLMs in clinical scenarios related to breast cancer in China. Overall, LLMs can serve as effective tools for Chinese patients with breast cancer to obtain health information, helping to address the majority of concerns related to diagnosis, treatment, recovery, and follow up of this population. However, in the context of breast cancer specialists, the accuracy, practicality, and relevance of LLMs’ responses need improvement. We propose a multidimensional optimization framework to enhance the utility and reliability of ChatGPT in breast cancer diagnosis and management. On the one hand, the model should be trained using high-quality medical data, such as the latest breast cancer research, clinical guidelines, and case reports, to improve its accuracy and practicality in the professional domain. On the other hand, under ethical compliance, ChatGPT should be connected to deidentified electronic health records, laboratory systems, and imaging databases to access real-time patient data and provide more personalized recommendations. Based on our research, ChatGPT-E demonstrates better repeatability, accuracy, and practicality in its responses compared to other LLMs. Therefore, it is recommended that Chinese patients with breast cancer translate their questions into English before querying ChatGPT, to improve its effectiveness. In addition, considering the potential data security, ethical, and legal risks of LLMs in clinical practice, it is essential to strengthen regulation of the training and application of LLMs in the medical professional field [34]. This study has certain limitations as the response from LLMs were not applied in real time to address the questions of patients with breast cancer or to assist doctors in making clinical decisions. We also did not evaluate all issues related to breast cancer. The data collection for this study was completed in January 2024. However, certain models (eg, GPT-4-turbo and DeepSeek) had not been publicly released at that time or failed to provide stable interfaces for academic research applications and therefore were not included in this study. Additionally, this study used structured questionnaires to evaluate the responses of LLMs, which ensured standardized assessment but partially limited the assessment of LLMs’ ability to handle open-ended, unstructured, and interactive questions. Future iterations could incorporate open-ended or interactive question types to better simulate real-world clinical consultations. Lastly, patient and expert user feedback can provide critical user-perspective data, address the limitations of existing expert-only evaluations, and enhance the application effectiveness and user experience of LLMs in health care. Further research is required to evaluate the real-world clinical effectiveness of LLMs and the real user experience of patients with breast cancer in China.

## Supplementary material

10.2196/66429 Multimedia Appendix 1 Specific contents of patient questionnaire and expert questionnaire and LLMs' response to them.

10.2196/66429 Multimedia Appendix 2 Comparison of generalization-specificity score (GSS).

10.2196/66429 Multimedia Appendix 3 Percentage distribution of ratings.

10.2196/66429 Multimedia Appendix 4 Comparison of overall accuracy, practicality, and generalization-specificity score (GSS) between patient and expert questionnaires using Mann-Whitney U Test.

10.2196/66429 Multimedia Appendix 5 Multiple hypothesis testing (Dunn test) results for overall accuracy, practicality, and generalization-specificity score (GSS) of patient questionnaires among different models.

10.2196/66429 Multimedia Appendix 6 Multiple hypothesis testing (Dunn test) results for overall accuracy, practicality, and generalization-specificity score (GSS) of expert questionnaires among different models.

10.2196/66429 Multimedia Appendix 7 Results of multiple hypothesis tests (Dunn test) on the accuracy, practicality, and generalization-specificity score (GSS) of specific questions of patient questionnaires across different models.

10.2196/66429 Multimedia Appendix 8 Results of multiple hypothesis tests (Dunn test) on the accuracy, practicality, and generalization-specificity score (GSS) of specific questions of expert questionnaires across different models.

## Abbreviations

ChatGPT-C: ChatGPT-Chinese
ChatGPT-E: ChatGPT-English
EB: ERNIE Bot
GSS: generalization-specificity score
ICC: intraclass correlation coefficient
LLM: large language model
PIPL: Personal Information Protection Law
