# Supplementary material for: Large Language Models as a Consulting Hotline for Patients With Breast Cancer and Specialists in China: Cross-Sectional Questionnaire Study
Source: JMIR Med Inform. 2025 May 27;13:e66429. doi: 10.2196/66429 (PMC12133073; doi:10.2196/66429)
Supplement: Multimedia Appendix 1 [file medinform-v13-e66429-s001.docx]

**Supplementary Table 1.** Specific contents of patient questionnaire and expert questionnaire and LLMS' response to them.

| **Qusetion（English)** | **ChatGPT-E** | | **ChatGPT-C** | | **ERNIE Bot** | | |
| --- | --- | --- | --- | --- | --- | --- | --- |
|  | **Responses 1** | **Responses 2** | **Responses 1** | **Responses 2** | **Responses 1** | **Responses 2** | |
| **Patient Questionnaires** |  |  |  |  |  |  | |
| 1. Is breast cancer hereditary, and will it have an impact on my descendants? | Yes, breast cancer can have a hereditary component. About 5-10% of breast cancer cases are thought to be hereditary, meaning they result directly from gene mutations passed on from a parent. The most well-known genes associated with hereditary breast cancer are BRCA1 and BRCA2. Mutations in these genes significantly increase the risk of developing breast cancer, as well as ovarian and some other cancers.  If you have a family history of breast cancer, especially if multiple close relatives have been affected or if they were diagnosed at a young age, it's advisable to speak with a genetic counselor. They can help assess your risk and determine if genetic testing is appropriate.  As for the impact on descendants, if you carry a mutation in a gene associated with hereditary breast cancer (such as BRCA1 or BRCA2), there's a possibility you could pass that mutation on to your children. This would potentially increase their risk of developing breast cancer or other associated cancers later in life. Genetic counseling can also help your descendants understand their own risk factors and take appropriate preventive measures, such as increased screening or risk-reducing surgeries, if necessary. It's essential to remember that not everyone who inherits a mutation will develop cancer, but knowing about the increased risk allows for proactive management and monitoring. | similar | 乳腺癌通常被认为是一种复杂的疾病，其发病原因涉及到遗传因素和环境因素。遗传因素在乳腺癌的发病中起着一定的作用，但并不是唯一的影响因素。  **遗传因素**：  1. **BRCA1和BRCA2基因突变**：这两种基因的突变与乳腺癌以及其他癌症的风险增加相关。拥有这些突变的人群乳腺癌的发病风险较高，而这些突变也可能会遗传给后代。  2. **其他基因变异**：除了BRCA1和BRCA2，其他一些基因的变异也与乳腺癌的发生有关，但它们的影响可能不如BRCA基因突变那么显著。  **环境因素**：  1. **生活方式**：饮食、运动、吸烟等生活习惯对乳腺癌的发生有一定影响。  2. **激素因素**：激素水平的变化可能会增加乳腺癌的风险，比如早经期、晚绝经、未生育、晚育等。  **对后代的影响**：  如果一个人携带BRCA1或BRCA2基因的突变，他们的子女有50%的机会继承这种突变。继承这种突变并不意味着一定会患上乳腺癌，但会增加患病的风险。因此，如果一个家庭中有乳腺癌遗传史，特别是BRCA1或BRCA2突变的遗传史，建议家庭成员进行遗传咨询和遗传测试，以便更好地了解自己的风险并采取相应的预防措施。  总的来说，乳腺癌的遗传风险是存在的，但环境因素同样重要。及早的筛查、健康的生活方式和家族史的关注都是预防乳腺癌的重要措施。如果您担心自己或您的家族患上乳腺癌，建议咨询医生进行进一步的评估和建议。 | similar | 乳腺癌确实具有一定的遗传倾向，但并不意味着它一定会直接遗传给后代。让我详细解释一下：  乳腺癌的遗传风险主要来自于特定的基因突变，其中最著名的是BRCA1和BRCA2基因。当这些基因发生突变时，乳腺癌的风险确实会增加。然而，只有大约5-10%的乳腺癌病例与这些遗传性基因突变直接相关。  如果你的家族中有乳腺癌病史，特别是直系亲属（如母亲、姐妹或女儿）中有乳腺癌患者，那么你的乳腺癌风险可能会增加。但这并不意味着你一定会患上乳腺癌，只是风险相对较高。  对于你的后代来说，如果你的家族中存在BRCA1或BRCA2等遗传性基因突变，他们可能会继承这些突变基因，从而增加患乳腺癌的风险。然而，即使继承了这些突变基因，也并不意味着他们一定会患上乳腺癌。  为了更准确地了解乳腺癌的遗传风险和家族史对你的影响，建议咨询专业的遗传咨询师或医生。他们可以根据你的具体情况提供更详细的建议和指导，包括可能的基因检测和预防措施。  总之，乳腺癌确实具有一定的遗传倾向，但并不意味着它一定会直接遗传给后代。了解你的家族史和可能的遗传风险，以及采取适当的预防措施，可以帮助你和你的后代降低乳腺癌的风险。  请注意：以上回答仅供参考，不能作为医学诊断、治疗依据或指导建议。请您随时关注身体状况，切勿盲目用药或治疗，如遇病情变化或感觉任何不适，建议您及时向专业人士求助，可线上咨询医生，或者去线下医疗机构就诊。 | similar |  |
| 2. What impact does the staging of breast cancer have on treatment and prognosis? | Certainly, I can provide information on how the staging of breast cancer influences treatment and prognosis.  **Staging of Breast Cancer:**  Breast cancer staging is a crucial aspect of determining the extent of the disease and guiding treatment decisions. The staging system commonly used is the TNM system, which takes into account the size of the tumor (T), whether the cancer has spread to nearby lymph nodes (N), and whether it has metastasized to distant organs (M).  **Impact on Treatment:**  1. **Early Stages (I and II):**  - *Treatment Options:* Generally, for early-stage breast cancer, local treatments like surgery (lumpectomy or mastectomy) are common. Adjuvant therapies such as radiation therapy and hormone therapy may also be recommended based on the specific characteristics of the tumor.  - *Prognosis:* Early-stage breast cancer often has a better prognosis as the cancer is localized and hasn't spread extensively.  2. **Locally Advanced (Stage III):**  - *Treatment Options:* Treatment for locally advanced breast cancer usually involves a combination of surgery, chemotherapy, and possibly radiation therapy. Neoadjuvant therapy (given before surgery) might be used to shrink the tumor.  - *Prognosis:* Prognosis is generally less favorable than early stages, but advances in treatment have improved outcomes.  3. **Metastatic (Stage IV):**  - *Treatment Options:* Stage IV breast cancer is considered incurable, but treatments aim at controlling the disease, alleviating symptoms, and prolonging life. Systemic therapies such as chemotherapy, targeted therapy, and hormone therapy are commonly used.  - *Prognosis:* Prognosis is often less optimistic due to the presence of distant metastases, but advancements in targeted therapies have improved survival rates.  **Importance of Staging:**  1. **Treatment Tailoring:** Staging helps tailor treatment plans to the specific characteristics and spread of the cancer, optimizing the chances of successful treatment.  2. **Prognostic Information:** Staging provides valuable prognostic information, helping both patients and healthcare professionals understand the likely course of the disease and expected outcomes.  3. **Clinical Trials:** Staging is crucial in identifying eligible patients for clinical trials testing new therapies. These trials contribute to ongoing advancements in breast cancer treatment.  In conclusion, the staging of breast cancer is a critical factor influencing treatment decisions and prognosis. Tailored approaches based on the extent of the disease contribute to more effective management and improved outcomes for patients. | similar | 乳腺癌的分期对治疗和预后有着重要的影响。乳腺癌分期是根据肿瘤的大小、淋巴结的受累情况以及是否有远处转移等因素来确定的。通常分为0期到IV期。  1. **0期和I期：**  - *影响：* 这些早期阶段通常意味着肿瘤较小，局限于乳腺。治疗相对较为成功，预后较好。  - *治疗：* 一般采用手术切除肿瘤，可能会联合放疗或激素治疗。  2. **II期：**  - *影响：* 肿瘤较大，可能涉及附近的淋巴结。治疗复杂度增加，但仍有良好的治愈机会。  - *治疗：* 通常需要手术切除，可能伴随放疗、化疗或激素治疗。  3. **III期：**  - *影响：* 肿瘤更大，可能已扩散至淋巴结。治疗更为侧重全身治疗，预后相对较差。  - *治疗：* 通常需要全身性的治疗，如化疗、放疗，可能在手术前或后进行。  4. **IV期：**  - *影响：* 肿瘤可能已经扩散到其他器官，称为远处转移。治疗主要以缓解为主，治愈机会相对较低。  - *治疗：* 以化疗、激素治疗、靶向治疗和放疗为主，旨在减缓病情进展，提高生活质量。  分期的确定有助于制定个体化的治疗方案，提高治疗的精准性和效果。总体而言，早期发现和治疗乳腺癌有助于更好地控制病情，提高生存率。然而，对于晚期患者，治疗更多地偏向于缓解症状和提高生活质量。务必在医生的指导下进行治疗决策，因为每个患者的病情都是独特的。 | 乳腺癌的分期是评估肿瘤在患者身体内的扩散程度的重要指标，对治疗选择和预后评估都有着重要的影响。  ### 分期系统 乳腺癌通常使用 TNM 分期系统来描述肿瘤的大小和扩散程度：  1. **Tumor (Tumor Size)**: 肿瘤的大小和位置 2. **Node (Lymph Node Involvement)**: 淋巴结的受累情况 3. **Metastasis (Metastatic Spread)**: 是否有转移  这些信息有助于确定肿瘤的严重程度，并帮助医生选择最佳的治疗方案。  ### 影响因素  1. **治疗选择**：  - 早期乳腺癌（分期 0、I 和 II）可能会选择手术切除肿瘤，通常伴随辅助治疗，如放疗、化疗或内分泌治疗。  - 晚期乳腺癌（分期 III 和 IV）需要更侵入性的治疗，如更广泛的手术、放疗和化疗，以及靶向治疗等。  2. **预后评估**：  - 早期诊断的乳腺癌通常有更好的预后，因为肿瘤尚未扩散到其他部位，治疗效果更好。  - 晚期乳腺癌的治疗通常更具挑战性，预后相对较差，因为肿瘤已经扩散到其他器官，治疗难度增加。  ### 临床意义  1. **指导治疗选择**：分期有助于医生确定最适合患者的治疗方案，以最大程度地提高治疗效果。 2. **预测预后**：分期也是预测患者预后的重要依据，可以帮助医生和患者了解疾病的发展和治疗后的预期结果。  综上所述，乳腺癌的分期对治疗选择和预后评估都具有重要的影响，早期诊断和治疗对于提高患者的生存率和生活质量至关重要。 | 乳腺癌的分期是评估肿瘤扩散程度和病情严重程度的重要指标。它对于确定治疗方案、预测患者的预后以及制定后续随访计划都具有至关重要的作用。  乳腺癌的分期通常基于以下几个方面：  肿瘤大小：即原发肿瘤在乳腺内的直径大小。 淋巴结状态：是否有癌细胞转移至腋窝淋巴结或其他相关淋巴结。 远处转移：癌细胞是否已扩散至身体的其他部位，如肺、肝、骨或脑。 基于上述因素，乳腺癌被分为0到IV期。以下是各分期对治疗和预后的影响：  0期（原位癌）： 治疗：通常通过手术切除肿瘤，有时可能辅以放疗或激素治疗。 预后：预后非常好，五年生存率接近100%。 I期（早期）： 治疗：手术切除肿瘤，可能辅以放疗、化疗或激素治疗。 预后：预后良好，五年生存率很高。 II期（中期）： 治疗：手术切除肿瘤，通常需要辅助放疗、化疗和/或激素治疗。 预后：预后相对较好，但五年生存率较I期略低。 III期（局部晚期）： 治疗：可能需要更激进的手术，如乳房切除术，以及放疗、化疗和激素治疗的组合。 预后：预后较差，五年生存率显著降低。 IV期（晚期或转移性）： 治疗：治疗目标是控制疾病进展和缓解症状，可能包括化疗、放疗、激素治疗和靶向治疗。 预后：预后最差，五年生存率很低。治疗的主要目的是延长生存期和改善生活质量。 总体而言，乳腺癌的分期越早，治疗选择越多，预后也越好。因此，早期发现和治疗乳腺癌至关重要。定期的乳腺筛查和自检可以帮助早期发现乳腺异常，从而提高治愈率和生活质量。 | 乳腺癌的分期是评估乳腺癌严重程度和扩散程度的重要方法。它基于肿瘤的大小、是否已扩散到淋巴结或身体其他部位，以及肿瘤的生物学特性等因素。乳腺癌的分期通常使用TNM系统，其中T表示原发肿瘤的大小，N表示淋巴结的受累情况，M表示是否有远处转移。  乳腺癌的分期对治疗和预后有着显著的影响，具体表现在以下几个方面：  治疗方案选择： 早期乳腺癌（如I期和部分II期）：这些患者通常可以通过手术切除肿瘤，可能伴随化疗、放疗或激素治疗来减少复发的风险。保乳手术（乳房肿瘤切除术）或全乳切除术是常见的选择。 局部晚期乳腺癌（部分II期和III期）：这些患者可能需要更积极的治疗，包括新辅助化疗（在手术前进行化疗以缩小肿瘤），然后进行手术。放疗通常也是治疗计划的一部分。 晚期或转移性乳腺癌（IV期）：在这个阶段，癌症已经扩散到身体的其他部位（如肺、肝、骨或脑）。治疗的主要目的是控制症状、延长生存期和维持生活质量。化疗、激素治疗、靶向治疗和/或放疗可能会被用来帮助管理疾病。 预后评估： 乳腺癌的分期是预后的重要因素。通常来说，早期乳腺癌患者的预后比晚期患者要好，因为早期癌症更容易通过手术和其他治疗手段控制。 分期还影响患者的5年生存率。例如，I期乳腺癌患者的5年生存率通常很高（接近90%或更高），而IV期患者的5年生存率则显著降低。 临床决策和监测： 知道乳腺癌的分期有助于医生制定个性化的治疗计划，确定随访的频率和类型，并监测疾病的可能复发。 分期信息也用于临床研究，帮助科学家了解乳腺癌的生物学行为，开发更有效的治疗方法。 总的来说，乳腺癌的分期是确定治疗策略、评估患者预后和进行临床研究的关键因素。因此，准确地进行乳腺癌分期对于确保患者获得最佳治疗和护理至关重要。 |  |
| 3. What are the treatment methods for breast cancer, and which one should I choose? | Certainly! When it comes to breast cancer treatment, several methods may be employed, often used in combination depending on the stage, type, and characteristics of the cancer. Here are the primary treatment options:  1. **Surgery:**  - **Lumpectomy:** Also known as breast-conserving surgery, this involves removing the tumor and a small margin of surrounding tissue.  - **Mastectomy:** This involves removing the entire breast. There are different types of mastectomy, including:  - Total mastectomy (removal of the entire breast)  - Modified radical mastectomy (removal of the entire breast and some lymph nodes)  - Radical mastectomy (removal of the entire breast, lymph nodes, and chest wall muscles)  2. **Radiation Therapy:**  - Radiation therapy uses high-energy rays to kill cancer cells. It is often used after surgery to kill any remaining cancer cells in the breast, chest wall, or underarm area.  3. **Chemotherapy:**  - Chemotherapy uses drugs to kill cancer cells or stop them from growing. It can be given before surgery to shrink tumors, after surgery to kill any remaining cancer cells, or in cases of metastatic breast cancer to slow the cancer's growth.  4. **Hormone Therapy:**  - Hormone therapy is used to treat hormone receptor-positive breast cancers, which grow in response to hormones like estrogen or progesterone. It works by blocking the body's production of these hormones or by blocking the hormones from attaching to cancer cells.  5. **Targeted Therapy:**  - Targeted therapy targets specific molecules or pathways involved in the growth and spread of cancer cells. It is often used to treat HER2-positive breast cancer, which overexpresses the HER2 protein.  6. **Immunotherapy:**  - Immunotherapy boosts the body's immune system to help fight cancer. While not as commonly used in breast cancer treatment as in some other types of cancer, it may be an option in certain cases, particularly in advanced or metastatic breast cancer.  The choice of treatment depends on various factors, including the stage and characteristics of the cancer, as well as the patient's overall health and preferences. It's essential to discuss your options thoroughly with your healthcare team to determine the most suitable treatment plan for your specific situation. They can provide personalized recommendations based on your individual needs and circumstances. | similar | 乳腺癌的治疗方式通常取决于患者的具体情况，包括癌症的类型、大小、分级、扩散程度以及患者的年龄和健康状况等因素。一般来说，乳腺癌的治疗方式可以包括以下几种：  1. **手术治疗：** 手术是乳腺癌治疗的主要方式之一。根据肿瘤的类型和大小，手术可以分为乳房保留手术（如部分切除术）和乳房切除手术（全乳房切除术或根治性切除术）两种。手术通常是早期乳腺癌的首选治疗方法之一。  2. **放射治疗：** 放射治疗常常在手术后进行，旨在杀死可能残留在乳腺组织中的癌细胞，降低复发的风险。放射治疗可以通过定向放射线照射到肿瘤区域来实现。  3. **化学疗法：** 化学疗法使用药物来杀死或阻止癌细胞生长。这种治疗方法常常在手术前或手术后进行，以帮助缩小肿瘤、减少扩散并降低复发风险。  4. **内分泌治疗：** 内分泌治疗主要针对激素受体阳性的乳腺癌，通过阻断或降低体内激素水平来抑制癌细胞生长。  5. **靶向治疗：** 靶向治疗是针对乳腺癌特定的生物标志物或信号通路的治疗方法，能够更精准地攻击癌细胞而减少对健康组织的伤害。  6. **免疫治疗：** 免疫治疗利用患者自身的免疫系统来攻击癌细胞。这种治疗方式在某些乳腺癌患者中可能会产生良好的效果。  选择合适的治疗方式需要与医生进行充分的讨论和评估。医生会根据患者的病情、个体差异以及治疗目标制定最佳的治疗方案。因此，建议您与您的医生密切合作，共同决定最适合您的治疗方案。 | similar | 乳腺癌的治疗方式主要包括手术、放疗、化疗、激素治疗和靶向治疗等。这些治疗方法的选择取决于乳腺癌的类型、分期、患者的整体健康状况以及个人偏好。以下是对这些治疗方式的简要介绍：  手术治疗：手术是治疗乳腺癌的主要方法，包括乳房保留手术（如局部切除或区段切除）和乳房切除术（如全乳切除）。手术的选择取决于肿瘤的大小、位置和患者的需求。对于早期乳腺癌，手术通常能够取得较好的治疗效果。  放疗：放疗是利用高能射线杀死癌细胞的治疗方法。它通常在手术后进行，以降低局部复发的风险。在某些情况下，放疗也可以作为手术前的辅助治疗，以缩小肿瘤并提高手术的成功率。  化疗：化疗是使用药物杀死癌细胞的治疗方法。它可以通过静脉注射或口服药物的方式进行。化疗通常用于手术前以缩小肿瘤、手术后以杀死可能残留的癌细胞，或作为晚期乳腺癌的主要治疗方法。  激素治疗：激素治疗是通过调节患者体内的激素水平来抑制乳腺癌细胞的生长。这种治疗方法主要用于激素受体阳性的乳腺癌患者。常见的激素治疗药物包括他莫昔芬和芳香化酶抑制剂等。  靶向治疗：靶向治疗是针对乳腺癌细胞特定的分子靶点进行治疗的方法。这种治疗方法能够更精确地杀死癌细胞，同时减少对正常细胞的损伤。常见的靶向治疗药物包括曲妥珠单抗和帕妥珠单抗等。  在选择治疗方式时，您应该与医生进行充分的沟通和讨论，了解各种治疗方法的优缺点、可能的风险和副作用以及预期的治疗效果。医生会根据您的具体情况制定个性化的治疗方案，以帮助您做出最佳的治疗选择。同时，您还可以考虑咨询第二位医生或寻求专业的乳腺癌治疗机构的意见，以确保您做出明智的决策。 | similar |  |
| 4. What are the various surgical treatment methods for breast cancer, and how do they each impact the appearance of the breast? | Hey there! Sure, let's dive into the surgical treatment methods for breast cancer and their impact on breast appearance.  1. **Lumpectomy (Breast-Conserving Surgery):**  - **Procedure:** This involves removing the tumor and a small amount of surrounding healthy tissue, preserving the majority of the breast.  - **Impact on Appearance:** Lumpectomy typically results in minimal changes to breast appearance. However, there might be some scarring, and the breast may look slightly different post-surgery.  2. **Mastectomy:**  - **Procedure:** Mastectomy is the removal of the entire breast. There are different types, including simple (removal of the breast tissue), modified radical (removal of breast tissue and some lymph nodes), and radical (removal of breast tissue, lymph nodes, and chest muscles).  - **Impact on Appearance:** The impact is significant, as the entire breast is removed. Reconstruction options are available to restore breast shape, but there will be scarring and a different look compared to the natural breast.  3. **Breast Reconstruction:**  - **Procedure:** This can be done either at the time of mastectomy or delayed. It involves rebuilding the breast using implants or the patient's own tissue.  - **Impact on Appearance:** Reconstruction aims to restore a natural-looking breast, but it won't be identical to the original. Scarring will depend on the reconstruction method.  4. **Sentinel Lymph Node Biopsy:**  - **Procedure:** Involves the removal and examination of a few lymph nodes to check for cancer spread.  - **Impact on Appearance:** While this doesn't directly alter breast appearance, it's often done in conjunction with other surgeries, impacting the overall surgical experience.  5. **Axillary Lymph Node Dissection:**  - **Procedure:** Removal of more lymph nodes than in sentinel node biopsy.  - **Impact on Appearance:** Similar to sentinel node biopsy, the direct impact on appearance is minimal, but it's often part of a larger surgical plan.  6. **Reconstructive Techniques:**  - **Procedure:** Techniques like oncoplastic surgery involve reshaping the remaining breast tissue to maintain symmetry after lumpectomy.  - **Impact on Appearance:** These techniques aim to minimize the aesthetic impact of breast-conserving surgery.  It's important to note that each person's experience is unique, and factors like the stage of cancer, individual anatomy, and personal choices influence the outcome. If you have specific concerns, discussing them with your surgeon can provide tailored information based on your situation. | similar | 乳腺癌的手术治疗方式主要包括乳房保留手术（Breast-Conserving Surgery, BCS）和乳房切除手术（Mastectomy）。这两种手术方式对乳房外观有不同的影响。  1. **乳房保留手术（Breast-Conserving Surgery, BCS）:**  - **描述：** 也称为部分切除术或切除肿瘤术，是一种保留乳房部分组织的手术，通常包括肿瘤及其周围一些正常组织。  - **外观影响：** BCS致力于保留乳房的自然形状和外观，尽量减小手术对乳房外观的影响。通常，患者的乳房外观相对保持完整，但可能会有一些瘢痕和变形。  2. **乳房切除手术（Mastectomy）:**  - **描述：** 这是一种完全切除患乳房的手术，可以分为单侧切除（单侧乳房切除术）和双侧切除（双侧乳房切除术）。  - **外观影响：** Mastectomy明显地改变了乳房的外观，因为整个乳房被移除。患者可能会在术后感觉到失去了一侧或双侧的乳房，这对一些女性来说可能是心理和情感上的挑战。  在许多情况下，医生和患者一起讨论最适合的手术选项，考虑到肿瘤的大小、位置、患者的整体健康状况以及个体对外观的关注。此外，随着医学技术的不断发展，整形手术和乳房重建手术等方法也可以帮助改善手术后的外观。 | similar | 作为乳腺癌专家，对于乳腺癌手术治疗方式及其对乳房外观的影响有深入了解。以下是乳腺癌的三种手术治疗方式，以及它们对乳房外观的影响：  乳腺癌改良根治术：这种手术方式切除整个乳房、胸大肌、胸小肌、腋窝和淋巴结。由于手术范围较大，术后乳房的外观会受到较大影响，通常需要进行乳房重建或佩戴义乳来恢复外观。 乳腺癌保乳手术：这种手术方式仅切除肿瘤及其周围的一部分组织，保留大部分乳房。手术后乳房的外观相对较好，但需要在手术后进行放疗和化疗，以降低复发的风险。 乳腺癌前哨淋巴结活检：这种手术方式仅切除前哨淋巴结（最先接受肿瘤细胞转移的淋巴结），如果前哨淋巴结没有转移，则不进行进一步的淋巴结清扫。手术后乳房的外观影响较小，但需要密切随访，以确保没有淋巴结转移。 需要注意的是，具体的手术方式应根据患者的病情、年龄、美容需求等进行综合考虑，以达到最佳的治疗效果。 | 乳腺癌的手术治疗方式主要根据肿瘤的大小、位置以及患者的具体情况来选择。以下是几种常见的手术治疗方式及其对乳房外观的影响：  乳房保留手术（保乳手术）： 手术过程：在这种手术中，仅切除肿瘤和周围一小部分正常组织，而不是整个乳房。通常还会进行腋窝淋巴结活检或清除，以检查癌细胞是否已扩散到淋巴结。 对乳房外观的影响：由于只切除了部分组织，乳房的基本形状和大小得以保留。然而，手术部位可能会有一些凹陷或变形，这取决于切除组织的多少和位置。随着时间的推移，这些变化可能会变得不那么明显。 乳房切除术： 手术过程：包括简单全乳切除术和改良根治性乳房切除术。前者切除整个乳房，但保留胸肌。后者则切除乳房、胸肌筋膜以及腋窝淋巴结。 对乳房外观的影响：乳房被完全切除，因此术后该侧胸部将无乳房组织。这会导致明显的身体不对称和外观上的改变。患者可以选择佩戴外部乳房假体（义乳）或进行乳房重建手术来改善外观。 乳房重建手术： 手术过程：这是一种可选手术，可以在乳房切除术后立即进行（即刻重建）或稍后进行（延期重建）。重建可以使用植入物（硅胶或盐水假体）或患者自身的组织（如背部肌肉、腹部脂肪等）。 对乳房外观的影响：乳房重建手术的目标是创建一个与对侧乳房相似的新乳房，以恢复身体的对称性和外观。重建乳房的感觉和外观可能无法与原始乳房完全相同，但许多患者对此感到满意。 需要注意的是，每种手术方式都有其适应症和局限性，选择最适合患者的手术方式需要综合考虑多种因素，包括肿瘤特征、患者的健康状况和个人偏好。在做出决定之前，与外科医生进行充分的讨论和了解是非常重要的。 |  |
| 5. What is the total cost of treating breast cancer in China? | To accurately estimate the total cost of treating breast cancer in China, we need to consider several factors:  1. **Incidence and Prevalence**: The number of new cases diagnosed each year and the total number of individuals living with breast cancer in China influence the overall cost.  2. **Diagnostic Procedures**: Costs associated with screening, diagnosis, and staging of breast cancer including mammograms, ultrasounds, biopsies, and imaging studies.  3. **Treatment Modalities**: This includes the costs of surgery (mastectomy or lumpectomy), radiation therapy, chemotherapy, targeted therapy, hormone therapy, and immunotherapy.  4. **Medication Costs**: Expenses related to medications used in the treatment of breast cancer, including chemotherapy drugs, hormone therapy drugs, targeted therapy drugs, and supportive medications for managing side effects.  5. **Hospitalization and Outpatient Care**: Costs associated with hospital stays, outpatient visits, consultations with specialists, and supportive care services.  6. **Follow-Up Care and Surveillance**: Expenses related to long-term follow-up care, surveillance imaging, and monitoring for recurrence.  7. **Indirect Costs**: These include lost productivity due to illness, transportation costs for accessing treatment centers, and out-of-pocket expenses borne by patients and their families.  8. **Government Subsidies and Insurance Coverage**: The extent of government subsidies for cancer treatment programs and the level of insurance coverage available to patients also impact the overall cost burden.  9. **Research and Development**: Investment in research and development for new treatments and technologies also contributes to the overall cost of breast cancer care.  10. **Geographical Disparities**: Cost variations may exist between different regions within China due to differences in healthcare infrastructure, access to specialized care, and economic factors.  To provide an accurate estimate of the total cost, comprehensive data from healthcare institutions, government reports, and research studies specific to breast cancer in China would be required. This data would need to be analyzed to determine the average cost per patient, considering the various stages of diagnosis, treatment, and follow-up care. Additionally, projections for future costs would need to be factored in to account for the increasing burden of breast cancer as the population ages and incidence rates rise. | similar | 治疗乳腺癌的费用因个人病情、治疗方法和医疗机构而异。一般来说，治疗乳腺癌可能需要包括以下费用：  1. **手术费用**：包括手术前的检查费用、手术费用本身以及手术后的康复费用。  2. **放疗费用**：对于一些乳腺癌患者，放疗是常见的治疗方法之一，需要考虑放疗的费用。  3. **化疗费用**：化疗是治疗乳腺癌的常见方法之一，涉及到药物费用以及输液等费用。  4. **靶向治疗费用**：对于一些乳腺癌患者，靶向治疗可能是必要的，需要考虑相关药物的费用。  5. **药物费用**：除了化疗和靶向治疗，还可能需要其他药物进行辅助治疗，这些药物的费用也需要考虑。  6. **住院费用**：治疗过程中可能需要住院，需要考虑住院期间的费用，包括床位费、护理费等。  7. **其他费用**：可能还包括康复费用、康复辅助器具费用等。  总体来说，治疗乳腺癌的费用可能会很高，需要根据个人情况和医疗方案进行具体评估。在中国，一般来说，乳腺癌的治疗费用可以从几万元到数十万元不等。同时，医保政策和医院的级别也会影响患者的实际负担。建议患者在治疗前咨询医生，了解清楚相关费用和医保政策，以便做出合适的决策。 | similar | 作为乳腺癌专家，我深知治疗乳腺癌的费用因多种因素而异，包括疾病的严重程度、治疗方案的选择、所在地区的医疗水平以及患者的个人情况等。因此，很难给出一个确切的费用数字。  在中国，乳腺癌的治疗费用主要包括以下几个方面：  诊断费用：包括乳腺超声、乳腺X线摄影（钼靶）、核磁共振（MRI）等影像学检查，以及可能的活检和病理检查等。这些检查的费用因医院和地区而异。  手术费用：手术是治疗乳腺癌的重要手段之一，费用因手术类型和复杂程度而异。一般来说，乳腺切除术和淋巴结清扫术等手术费用较高。  放化疗费用：根据患者的具体病情，可能需要进行放疗或化疗。这些治疗的费用因方案、药物种类和治疗周期而异。  康复和随访费用：乳腺癌患者在治疗后需要进行康复和长期的随访，包括定期检查、药物治疗和心理咨询等。这些费用也会因患者的具体情况而异。  总体来说，乳腺癌的治疗费用是一个相对较大的经济负担。为了减轻患者的经济压力，中国政府采取了一系列措施，包括提高医保报销比例、设立大病保险等。此外，一些慈善组织和基金会也提供了针对乳腺癌患者的救助项目。  需要注意的是，以上信息仅供参考，实际费用可能因各种因素而有所不同。建议患者在确诊后，与所在地区的医院和医保部门进行详细咨询，以了解具体的治疗费用和相关政策。 | similar |  |
| 6. What aspects are included in the postoperative rehabilitation training for breast cancer, and what benefits does it bring to rehabilitation | Certainly! Postoperative rehabilitation training for breast cancer involves a multidimensional approach to address the physical, emotional, and functional aspects of recovery. Let's delve into the key aspects and benefits:  ### Aspects of Postoperative Rehabilitation Training for Breast Cancer:  1. **Physical Exercise:**  - **Range of Motion (ROM) Exercises:** To maintain and restore flexibility in the affected arm and shoulder.  - **Strength Training:** Gradual introduction of resistance exercises to regain muscle strength.  - **Cardiovascular Exercise:** Aerobic activities to improve overall fitness and stamina.  2. **Lymphedema Management:**  - **Manual Lymphatic Drainage (MLD):** Gentle massage techniques to reduce lymphedema.  - **Compression Garments:** Wearing specially designed garments to control swelling.  3. **Pain Management:**  - **Physical Therapy Techniques:** Targeted interventions to alleviate postoperative pain and discomfort.  - **Breathing Exercises:** Techniques to manage pain and improve lung function.  4. **Psychosocial Support:**  - **Counseling and Support Groups:** Emotional support to cope with the psychological impact of breast cancer and surgery.  - **Mind-Body Practices:** Incorporating techniques like mindfulness and relaxation to reduce stress.  5. **Functional Rehabilitation:**  - **Activities of Daily Living (ADL) Training:** Assisting patients in regaining independence in daily tasks.  - **Occupational Therapy:** Tailored interventions to enhance functional abilities.  6. **Educational Programs:**  - **Nutritional Guidance:** Providing information on a balanced diet to support healing.  - **Health Education:** Informing patients about potential side effects, self-care practices, and signs of complications.  ### Benefits of Postoperative Rehabilitation Training:  1. **Faster Recovery:**  - Structured rehabilitation accelerates the recovery process, enabling patients to regain functionality sooner.  2. **Reduced Complications:**  - Physical therapy and exercise contribute to preventing complications such as lymphedema and joint stiffness.  3. **Improved Quality of Life:**  - Comprehensive rehabilitation enhances overall well-being by addressing both physical and emotional aspects.  4. **Enhanced Mobility and Functionality:**  - Targeted exercises help restore range of motion, strength, and functionality of the affected arm and shoulder.  5. **Psychological Well-being:**  - Psychosocial support aids in coping with the emotional challenges, fostering a positive mindset during recovery.  6. **Long-term Health Benefits:**  - Regular exercise and lifestyle modifications contribute to long-term health, reducing the risk of recurrence and promoting overall wellness.  7. **Patient Empowerment:**  - Educational programs empower patients with knowledge, enabling them to actively participate in their recovery journey.  In summary, postoperative rehabilitation for breast cancer is a holistic approach that addresses various facets of recovery, aiming not only to restore physical function but also to improve the overall quality of life for individuals who have undergone breast cancer surgery. | similar | 嗨！当然，我很乐意为您解答有关乳腺癌术后康复训练的问题。  乳腺癌术后康复训练是一个多方面的过程，旨在帮助患者尽早恢复身体功能，提高生活质量。以下是一些可能包括在康复训练中的方面，以及它们对康复的帮助：  1. **物理康复：** 物理康复是通过运动和锻炼来帮助患者恢复身体功能的关键部分。这可能包括肩部、手臂和胸部的特定运动，旨在增加关节灵活性，减轻僵硬感，以及逐步增加运动范围。  2. **心理康复：** 乳腺癌术后，患者可能经历心理上的挑战，包括焦虑、抑郁和身体形象问题。心理康复方面可能涉及心理治疗、支持小组，以及帮助患者调整和面对新的生活状态。  3. **营养指导：** 营养在乳腺癌术后康复中扮演重要角色。合理的饮食计划可以帮助患者维持健康体重，提供足够的营养支持，促进伤口愈合和整体康复。  4. **药物管理：** 康复期间，患者可能需要特定的药物管理，如止痛药或其他辅助治疗，以减轻不适感并促进康复。  5. **定期随访：** 医生和康复专业人员将定期进行随访，以监测患者的康复进展，调整治疗计划，并提供必要的支持和建议。  这些方面的综合应用可以提高患者的身体功能，减轻不适，促进心理健康，并帮助患者更好地适应生活的变化。康复过程应该是个性化的，根据每位患者的具体状况制定相应的计划。如果您有更具体的问题或需要详细解释，请告诉我！ | 乳腺癌术后康复训练是一个综合性的过程，旨在帮助患者恢复身体功能、提升生活质量，同时减轻手术和治疗带来的负担。以下是乳腺癌术后康复训练涵盖的主要方面：  1. **物理康复训练：** 包括肩关节的活动度恢复、手臂的力量训练以及身体其他部位的运动。这有助于缓解手术后的肌肉紧张和活动受限。  2. **深呼吸和呼吸锻炼：** 有助于改善患者的肺活量，减轻手术后可能出现的肺部合并症。  3. **康复性体育锻炼：** 包括适度的有氧运动和无氧运动，以促进整体的健康和心肺功能。  4. **乳腺癌患者心理康复：** 心理康复是康复过程中不可或缺的一部分。患者可能会面临身体形象变化、焦虑、抑郁等问题，心理康复有助于应对这些挑战。  5. **饮食和营养：** 营养师可以制定合理的饮食计划，以促进患者的身体恢复和免疫力提升。  6. **乳腺癌术后护理：** 学习正确的乳房护理方法，了解如何进行乳房自我检查，以及警觉乳腺癌复发的迹象。  7. **药物管理：** 如果有需要，患者可能需要药物管理，包括镇痛药、抗生素等，以确保手术部位的适当治疗。  8. **定期随访和检查：** 定期的医学随访和检查对于监测患者的康复进程、早期发现并处理任何潜在的问题至关重要。  这些康复训练方面相互关联，共同促使患者恢复到尽可能接近手术前的状态。定制个性化的康复计划对于每位患者都很重要，考虑到他们的特定病情、手术方式和身体状况。在整个康复过程中，医疗团队的支持和指导也是至关重要的。 | 乳腺癌术后康复训练是一个综合性的过程，它涵盖了多个方面，旨在帮助患者恢复身体功能，提高生活质量，并调整心理状态。以下是一些主要的康复训练方面以及它们对康复的帮助：  肢体功能训练： 术后，患者可能会因为手术切口、淋巴结清扫等原因，出现肩关节活动度受限、上肢水肿等问题。因此，进行上肢和肩部的功能训练至关重要。 通过逐渐增加的关节活动范围练习，可以帮助患者恢复肩关节的灵活性和力量。 这些训练还有助于预防或减轻上肢淋巴水肿，促进淋巴液的循环。 有氧运动： 适度的有氧运动，如散步、慢跑、游泳等，可以改善心肺功能，增强体质，帮助患者更好地应对治疗和康复过程中的体力挑战。 有氧运动还能促进新陈代谢，有助于控制体重，而维持健康体重对乳腺癌患者来说非常重要。 心理康复： 乳腺癌的诊断和治疗过程可能会给患者带来巨大的心理压力。因此，心理康复是术后康复训练中不可或缺的一部分。 通过心理咨询、支持小组、冥想和放松训练等方式，可以帮助患者调整心态，减轻焦虑和抑郁，增强抗癌信心。 日常生活能力训练： 术后，患者可能需要重新学习一些日常生活技能，如穿衣、洗澡、梳头等。通过针对性的训练，患者可以更快地适应身体的变化，恢复独立生活的能力。 淋巴水肿管理： 对于出现上肢淋巴水肿的患者，需要采取特殊的护理措施，如穿戴压力袖套、进行轻柔的按摩和特定的理疗等，以减轻水肿和不适。 营养指导： 合理的饮食对乳腺癌患者的康复至关重要。营养师可以根据患者的具体情况提供个性化的饮食建议，帮助患者保持良好的营养状态，支持身体的康复。 综上所述，乳腺癌术后康复训练是一个多方面的过程，需要综合考虑患者的身体、心理和社会需求。通过科学的康复训练，患者不仅可以恢复身体功能，还能提高生活质量，更好地回归正常生活。 | similar |  |
| 7. Is breastfeeding possible during breast cancer treatment? | Certainly! Breastfeeding during breast cancer treatment is generally not recommended. Breast cancer treatments, such as surgery, chemotherapy, and radiation therapy, can introduce substances into the body that might be harmful to the baby. Additionally, these treatments may interfere with the mother's ability to produce breast milk.  Chemotherapy drugs, for example, can be passed to the baby through breast milk, posing potential risks to the infant's health. Radiation therapy may also affect the breast tissue and compromise the mother's ability to breastfeed.  It's crucial for women undergoing breast cancer treatment to discuss their specific situation with their healthcare team. They can provide personalized advice based on the type and stage of cancer, the specific treatments involved, and the overall health of both the mother and the baby.  In many cases, healthcare providers may advise against breastfeeding during active treatment but may explore other options, such as using stored breast milk or formula. It's essential for women to communicate openly with their healthcare team to make informed decisions that prioritize both their health and the well-being of their baby.  If you have further questions or need more detailed information, feel free to ask! | Absolutely, I'd be happy to help. Keep in mind that while I can provide information, it's essential to consult with a healthcare professional for personalized advice.  During breast cancer treatment, the feasibility of breastfeeding depends on various factors, including the type and stage of cancer, the specific treatment plan, and the individual's overall health. In general, some treatments may interfere with breastfeeding, while others might not have a significant impact.  1. **Chemotherapy:** Certain chemotherapy drugs can pass into breast milk, making it unsafe for the baby. In such cases, breastfeeding is usually not recommended during active chemotherapy.  2. **Radiation therapy:** If the breast is treated with radiation, it may affect the ability to breastfeed from that particular breast. The healthcare team will guide on the safety and feasibility.  3. **Hormone therapy:** Some breast cancer treatments involve hormone therapy, which might also impact breastfeeding. It's crucial to discuss this with the healthcare provider.  4. **Surgery:** The type of surgery (e.g., lumpectomy, mastectomy) can influence the ability to breastfeed. A lactation consultant can assist in finding the best approach.  5. **Individual health:** The overall health and well-being of the mother play a role. If the mother's health is compromised during treatment, breastfeeding may not be advised.  In many cases, healthcare professionals may suggest alternatives such as pumping and storing breast milk before starting treatment, allowing for breastfeeding to resume later. It's crucial to have open communication with the healthcare team to make informed decisions based on individual circumstances.  Remember, this information is a general guide, and individual cases can vary. Always consult with your healthcare provider for advice tailored to your specific situation. | 当然，让我详细为您解释一下。在讨论乳腺癌治疗期间是否可以哺乳时，需要考虑具体情况。  一般来说，乳腺癌治疗包括手术、放疗、化疗和激素疗法等。这些治疗可能对哺乳产生影响，因此建议在开始治疗前与医生讨论。以下是一些建议：  1. **手术：** 如果进行了乳腺手术，可能影响乳腺的结构，从而影响哺乳。在手术后，医生会根据具体情况评估是否可以进行哺乳。  2. **放疗：** 放疗可能导致乳腺组织受损，影响哺乳的能力。医生通常会评估治疗后的乳腺健康状况，确定是否适合哺乳。  3. **化疗：** 化疗药物可能通过母乳传递给婴儿，因此在接受化疗期间通常不建议哺乳。在使用化疗药物时，医生可能建议中断哺乳或推迟哺乳计划。  4. **激素疗法：** 激素疗法可能影响女性的生育能力和乳腺健康。在接受激素疗法期间，哺乳可能受到限制。医生会根据病情和治疗方案制定建议。  总体而言，乳腺癌治疗期间哺乳的可行性取决于治疗方案、个体病情和治疗后的乳腺健康状况。在制定哺乳计划前，建议与医生讨论，以确保综合考虑了治疗的各个方面，保障母婴的健康。 | similar | 作为乳腺癌专家，对于乳腺癌及其治疗期间的各种问题有着深入的了解。其中，关于乳腺癌治疗期间是否能哺乳，这是一个较为复杂且需要细致考虑的问题。  首先，要明确的是，乳腺癌治疗期间涉及到多种治疗方式，如手术、化疗、放疗、内分泌治疗和靶向治疗等。这些治疗方式都可能对乳腺组织产生不同程度的影响，从而影响到哺乳功能。  手术治疗，特别是乳房切除术或乳腺部分切除术，可能会损害乳腺的导管和腺体，导致乳汁无法正常分泌或排出。化疗和放疗则可能通过影响乳腺细胞的增殖和分化，进一步影响哺乳功能。内分泌治疗和靶向治疗也可能对乳腺产生直接或间接的影响，从而影响哺乳。  除了治疗方式本身，治疗期间患者的身体状况和心理状态也是需要考虑的因素。治疗期间，患者可能会经历身体虚弱、恶心、呕吐、疲劳等不良反应，这些都可能影响到患者的哺乳能力和意愿。  此外，从婴儿健康的角度考虑，接受治疗期间母亲体内的药物成分可能会通过乳汁传递给婴儿，对婴儿的健康产生潜在的风险。  因此，综合考虑以上因素，一般不建议乳腺癌患者在治疗期间进行哺乳。当然，每个患者的情况都是独特的，最终的决定应该基于患者的具体情况、医生的建议以及婴儿的健康考虑。在做出决定之前，与医生进行充分的沟通和讨论是非常重要的。 | similar |  |
| 8. Why do I need systemic treatment (such as chemotherapy, endocrine therapy, or targeted therapy) after breast cancer surgery? | After breast cancer surgery, systemic treatment like chemotherapy, endocrine therapy, or targeted therapy may be recommended for several reasons:  1. **Eliminating Residual Cancer Cells**: Surgery aims to remove the visible tumor and any nearby tissue that might contain cancer cells. However, some cancer cells might still remain in the body, especially if the cancer has spread beyond the breast. Systemic treatments target these remaining cancer cells, reducing the risk of cancer recurrence.  2. **Treating Micrometastases**: Even if cancer has not spread to nearby lymph nodes or other organs at the time of surgery, there could still be micrometastases, tiny cancer cell clusters that are not detectable by imaging tests. Systemic treatments can target these micrometastases, preventing them from growing and causing metastatic disease.  3. **Reducing the Risk of Recurrence**: By targeting any remaining cancer cells or micrometastases, systemic treatments help reduce the risk of cancer recurrence. This is crucial for improving long-term outcomes and increasing the chances of survival.  4. **Tailoring Treatment to the Specific Type of Breast Cancer**: Different types of breast cancer may require different types of systemic treatment. For example, hormone receptor-positive breast cancers may benefit from endocrine therapy, while HER2-positive breast cancers may benefit from targeted therapy directed against the HER2 protein. Systemic treatments are often tailored based on the specific characteristics of the cancer to optimize effectiveness.  5. **Addressing Potential Spread**: Breast cancer has the potential to spread to other parts of the body, such as the bones, liver, lungs, or brain. Systemic treatments can help prevent or delay this spread, improving overall prognosis and quality of life.  6. **Improving Survival Rates**: Clinical trials and research studies have shown that adjuvant systemic therapy following surgery can significantly improve survival rates for breast cancer patients. These treatments have been shown to reduce the risk of cancer recurrence and improve overall survival outcomes.  7. **Comprehensive Treatment Approach**: Combining surgery with systemic therapy offers a comprehensive approach to treating breast cancer. Surgery removes the primary tumor, while systemic therapy addresses any remaining cancer cells throughout the body, providing a more thorough treatment strategy.  In summary, systemic treatment after breast cancer surgery plays a crucial role in eliminating residual cancer cells, treating micrometastases, reducing the risk of recurrence, tailoring treatment to the specific type of breast cancer, addressing potential spread, improving survival rates, and offering a comprehensive treatment approach. It is an integral part of the overall management plan for breast cancer patients and is aimed at improving long-term outcomes and quality of life. | similar | 在乳腺癌手术后需要进行全身治疗的原因是为了确保尽可能地清除患者体内任何可能残留的癌细胞，并提高治疗成功的概率。手术主要是为了切除肿瘤和周围组织，但它可能无法彻底消除体内所有癌细胞。  1. **防止复发和转移：** 全身治疗可以帮助阻止癌细胞的再生和扩散，减少复发的风险。即使肿瘤在乳腺中被完全切除，仍可能存在微小的癌细胞在其他部位。  2. **治疗残余癌细胞：** 癌细胞可能已经扩散到身体其他部位，即使在手术后肉眼看不见。全身治疗可以通过化学药物、激素或靶向治疗等方式，进入血液循环，攻击和摧毁远离原发病灶的癌细胞。  3. **提高治疗效果：** 全身治疗是一种综合性的治疗策略，可以协同手术的效果，提高治疗的整体成功率。不同的治疗方式可以有针对性地攻击癌细胞，降低病情进展的风险。  4. **预防复发：** 乳腺癌具有一定的复发风险，全身治疗有助于减少病情复发的可能性，提高患者的生存率。  具体的治疗方式，如化疗、内分泌治疗或靶向治疗，会根据患者的具体情况和病理学特征而定。例如，内分泌治疗主要用于激素受体阳性的乳腺癌，而靶向治疗则可以专门攻击某些癌细胞的特定分子。  总体而言，全身治疗是为了在手术之后更全面地应对患者的乳腺癌，提高治疗效果和生存率。 | similar | 作为乳腺癌专家，深知手术虽然可以切除乳腺中的肿瘤，但癌细胞有时会在手术前或手术过程中扩散到身体的其他部位，或者手术并未能完全清除所有的癌细胞。因此，在手术后，为了杀死这些可能残留的癌细胞并降低复发的风险，通常会建议患者接受全身治疗。  化疗：这是一种使用药物杀死癌细胞的治疗方法。由于化疗药物可以通过血液流动到达全身的各个部位，因此它可以杀死那些在原发肿瘤以外的地方生长的癌细胞。化疗通常在手术后进行，以杀死可能残留的癌细胞，从而降低复发的风险。 内分泌治疗：某些乳腺癌的生长依赖于雌激素。内分泌治疗通过降低体内的雌激素水平或阻止雌激素与癌细胞相互作用来阻止癌细胞的生长。这种治疗对于雌激素受体阳性的乳腺癌特别有效。 靶向治疗：这是一种针对癌细胞上特定分子的治疗方法。通过阻止这些分子，可以阻止癌细胞的生长和扩散。靶向治疗通常与化疗一起使用，以增强治疗效果。 总之，乳腺癌手术后的全身治疗是为了杀死可能残留的癌细胞，降低复发的风险，并提高治愈率。你的医生会根据你的具体病情和肿瘤的特性，为你制定最合适的治疗方案。  请注意，以上内容仅供参考，具体的治疗方案和效果可能因个体差异和医生建议略有不同，因此应与专业医疗团队密切合作，确保获得最适合的治疗和护理。 | similar |  |
| 9. What are the adverse reactions of drugs used in breast cancer treatment? | Certainly! In breast cancer treatment, various drugs are used, each with its own set of potential adverse reactions. Here's a breakdown of some common drugs used in breast cancer treatment and their adverse reactions:  ### 1. **Chemotherapy Drugs:**  - **Adverse Reactions:**  - **Nausea and Vomiting:** Chemotherapy drugs can irritate the stomach lining, leading to nausea and vomiting.  - **Hair Loss:** Many chemotherapy drugs can cause hair loss or thinning.  - **Fatigue:** Chemotherapy can cause extreme tiredness and fatigue.  - **Bone Marrow Suppression:** Chemotherapy can suppress bone marrow function, leading to decreased production of red blood cells (anemia), white blood cells (increased risk of infections), and platelets (increased risk of bleeding).  - **Peripheral Neuropathy:** Some chemotherapy drugs can cause nerve damage, leading to symptoms like tingling, numbness, and pain in the hands and feet.  - **Increased Risk of Infections:** Chemotherapy can weaken the immune system, increasing the risk of infections.  - **Mouth Sores:** Chemotherapy drugs can cause sores or ulcers in the mouth and throat.  ### 2. **Hormone Therapy:**  - **Adverse Reactions:**  - **Hot Flashes:** Hormone therapy can cause sudden feelings of warmth, sweating, and flushing of the face and body.  - **Vaginal Dryness and Discomfort:** Hormone therapy can lead to vaginal dryness and discomfort during intercourse.  - **Bone Loss:** Some hormone therapies can accelerate bone loss and increase the risk of osteoporosis.  - **Mood Swings and Depression:** Hormone therapy can affect mood and may lead to mood swings or depression.  - **Increased Risk of Blood Clots:** Certain hormone therapies can increase the risk of blood clots in the legs or lungs.  ### 3. **Targeted Therapy:**  - **Adverse Reactions:**  - **Cardiotoxicity:** Some targeted therapy drugs can affect the heart, leading to conditions like heart failure or abnormal heart rhythms.  - **Skin Rash:** Certain targeted therapy drugs can cause skin rashes or other skin reactions.  - **Liver Toxicity:** Targeted therapy drugs may affect liver function and lead to elevated liver enzymes.  - **Diarrhea:** Some targeted therapy drugs can cause diarrhea or gastrointestinal discomfort.  - **Fatigue:** Targeted therapy can also cause fatigue and weakness.  ### 4. **Immunotherapy:**  - **Adverse Reactions:**  - **Autoimmune Reactions:** Immunotherapy drugs can sometimes trigger autoimmune reactions, where the immune system attacks healthy tissues in the body.  - **Fatigue:** Fatigue is a common side effect of immunotherapy.  - **Skin Reactions:** Immunotherapy can cause skin reactions, such as rashes, itchiness, or blistering.  - **Flu-Like Symptoms:** Some patients may experience flu-like symptoms such as fever, chills, and muscle aches after receiving immunotherapy.  These adverse reactions can vary in severity and may require management or intervention by healthcare providers to minimize their impact on the patient's quality of life and treatment adherence. It's essential for patients to communicate any side effects they experience to their healthcare team promptly. | similar | 乳腺癌治疗药物可能引起各种不良反应，这些反应因药物类型和个体差异而异。以下是一些常见的乳腺癌治疗药物及其可能的不良反应：  1. **化疗药物**：  - 恶心和呕吐  - 脱发  - 疲劳感  - 免疫系统抑制，增加感染的风险  - 贫血  2. **激素疗法**：  - 激素水平的变化可能导致情绪波动  - 骨密度减少  - 热潮红和出汗  3. **靶向治疗**：  - 皮肤疹  - 腹泻  - 高血压  - 心脏问题的风险增加  4. **免疫疗法**：  - 免疫反应过度，导致炎症  - 甲状腺功能异常  - 肝功能异常  5. **放疗**：  - 皮肤反应，如红肿和脱屑  - 疲劳  - 口干  需要强调的是，每个患者对药物的反应都是独特的，不同的治疗方案和个体健康状况会影响不良反应的程度和类型。患者在接受治疗期间应密切关注任何不寻常的症状，并随时向医生报告，以便及时调整治疗计划。此外，医生会根据患者的整体健康状况来权衡治疗的利弊，以确保获得最佳的治疗效果。 | similar | 作为乳腺癌专家，我深知治疗乳腺癌的药物虽然有助于抑制和消灭癌细胞，但同时也可能带来一些不良反应。这些不良反应因药物种类、剂量以及个体差异而异，但通常包括以下几类：  骨髓抑制：这是化疗药物最常见的副作用之一。骨髓抑制会导致白细胞、红细胞和血小板数量下降，从而增加感染、贫血和出血的风险。 消化道反应：许多化疗药物会引起恶心、呕吐、腹泻或便秘等消化道症状。这些症状通常可以通过调整饮食和服用止吐药等方式来缓解。 肝脏和肾脏损伤：部分化疗药物可能对肝脏和肾脏造成损害，导致肝功能异常或肾功能减退。因此，在治疗期间需要密切监测肝肾功能。 心脏毒性：某些化疗药物，如蒽环类药物，可能对心脏产生毒性作用，导致心肌损伤、心律失常或心力衰竭等。在使用这些药物时，需要评估患者的心脏功能并谨慎用药。 过敏反应：一些患者可能对化疗药物产生过敏反应，如皮疹、瘙痒、呼吸困难等。在出现过敏反应时，应立即停药并采取相应的抗过敏治疗。 脱发：化疗药物可能导致头发脱落，这是许多患者非常担忧的副作用之一。但请放心，脱发通常是暂时性的，治疗结束后头发会重新长出。 其他：此外，化疗药物还可能引起神经毒性、口腔粘膜炎、手足综合征等其他不良反应。这些副作用的严重程度因个体差异而异，需要根据具体情况进行处理。 请注意，以上列举的不良反应并非所有乳腺癌患者都会出现。在制定治疗方案时，医生会根据患者的具体情况和药物特性来权衡利弊，力求在控制病情的同时减少不良反应的发生。同时，患者在治疗过程中应保持良好的心态和信心，积极配合医生的治疗建议。 | 乳腺癌的治疗药物多种多样，包括化疗药物、激素治疗药物、靶向治疗药物等。每种药物都可能带来一些不良反应，这些反应因药物种类、剂量以及个体差异而异。以下是一些常见乳腺癌治疗药物及其可能的不良反应：  化疗药物： 烷化剂：如环磷酰胺，可能引起恶心、呕吐、骨髓抑制（白细胞、血小板减少等）、脱发、膀胱刺激等。 抗代谢药：如甲氨蝶呤、氟尿嘧啶等，可能导致口腔炎、腹泻、皮疹、骨髓抑制等。 抗肿瘤抗生素：如阿霉素，可能引发心脏毒性（尤其是累积剂量较高时）、骨髓抑制、恶心、呕吐、脱发等。 植物类抗肿瘤药：如紫杉醇、多西他赛，常见的不良反应有过敏反应、骨髓抑制、神经毒性（如感觉异常、神经痛）、关节肌肉疼痛、脱发等。 激素治疗药物： 抗雌激素药物：如他莫昔芬，可能的不良反应包括潮热、阴道干燥、月经不规律、子宫内膜增厚或癌症风险增加等。 芳香化酶抑制剂：如来曲唑、阿那曲唑，常见副作用有骨质疏松、骨折风险增加、关节肌肉疼痛、潮热等。 促黄体生成激素释放激素（LHRH）类似物：如戈舍瑞林，可能导致潮热、阴道干燥、性欲减退、骨质疏松等。 靶向治疗药物： 表皮生长因子受体（HER2）抑制剂：如曲妥珠单抗、帕妥珠单抗，常见的不良反应包括心脏毒性（心功能不全、心力衰竭）、输液反应、腹泻等。 血管生成抑制剂：如贝伐单抗，可能的不良反应有高血压、出血、血栓形成、伤口愈合延迟等。 CDK4/6抑制剂：如哌柏西利，常见的副作用包括中性粒细胞减少、白细胞减少、感染风险增加、恶心、疲劳、肝酶升高等。 其他药物： 免疫治疗药物：如PD-1/PD-L1抑制剂，可能的不良反应包括免疫相关的不良反应，如肺炎、结肠炎、甲状腺炎、肝炎、肾炎等。 这些不良反应的严重程度和发生率因个体差异而异，且多数不良反应可以通过调整剂量、给予支持治疗或暂停治疗来控制和缓解。在乳腺癌治疗过程中，医生会密切监测患者的反应，并根据具体情况调整治疗方案。 |  |
| 10. How do I manage psychological and emotional health issues during the treatment of breast cancer? | Managing psychological and emotional health issues during breast cancer treatment is crucial for overall well-being and coping with the challenges associated with the disease. Here are some strategies to consider:  1. **Build a Support System**: Surround yourself with supportive friends, family members, and healthcare professionals who can provide emotional support and practical assistance throughout your treatment journey.  2. **Seek Counseling or Therapy**: Professional counseling or therapy can help you navigate the complex emotions that arise during cancer treatment. Therapists can offer coping strategies, provide a safe space to express your feelings, and help you develop resilience.  3. **Join Support Groups**: Connecting with others who are going through similar experiences can be incredibly comforting and validating. Support groups offer opportunities to share stories, exchange advice, and find solidarity with others facing breast cancer.  4. **Practice Mindfulness and Relaxation Techniques**: Engage in activities that promote relaxation and stress reduction, such as meditation, deep breathing exercises, yoga, or tai chi. These practices can help calm the mind, reduce anxiety, and improve overall well-being.  5. **Maintain Open Communication**: Communicate openly and honestly with your healthcare team about your emotional concerns and challenges. They can offer guidance, support, and referrals to mental health professionals if needed.  6. **Focus on Self-Care**: Prioritize self-care activities that promote physical and emotional well-being, such as regular exercise, healthy eating, adequate sleep, and engaging in hobbies or activities you enjoy.  7. **Educate Yourself**: Knowledge about your condition, treatment options, and potential side effects can help alleviate fear and anxiety. Stay informed by asking questions, researching reliable sources, and actively participating in your treatment decisions.  8. **Set Realistic Expectations**: Understand that it's normal to experience a range of emotions during cancer treatment, including fear, anger, sadness, and uncertainty. Be gentle with yourself and allow yourself to feel and process these emotions without judgment.  9. **Address Practical Concerns**: Financial worries, work-related stress, and logistical challenges can add to the emotional burden of cancer treatment. Seek assistance from social workers or financial counselors to address practical concerns and access resources for support.  10. **Stay Connected to What Brings You Joy**: Engage in activities and relationships that bring you joy, meaning, and purpose. Whether it's spending time with loved ones, pursuing creative outlets, or enjoying nature, nurturing your sense of joy can provide comfort and strength during difficult times.  Remember that managing psychological and emotional health is an ongoing process, and it's okay to seek help and support whenever you need it. By prioritizing self-care, seeking support from others, and staying informed about your treatment, you can navigate the emotional challenges of breast cancer with resilience and strength. | similar | 治疗乳腺癌是一个综合性的过程，不仅涉及身体健康，还涉及心理和情绪健康。以下是在治疗乳腺癌过程中管理心理和情绪健康问题的一些建议：  1. **建立支持系统：** 与家人、朋友和其他乳腺癌患者建立支持系统是至关重要的。分享感受和经验可以减轻心理压力。  2. **专业心理支持：** 寻求专业心理健康支持，如心理治疗或咨询服务。专业心理医生可以帮助您处理与癌症治疗相关的情绪和心理挑战。  3. **教育与信息：** 了解更多关于乳腺癌的信息，包括治疗过程、可能的副作用和康复阶段。了解更多信息有助于减轻对未知的恐惧感。  4. **参与支持小组：** 加入乳腺癌患者支持小组，与其他患者分享经验，获取实际建议，并建立互相理解的社交网络。  5. **保持积极心态：** 尽管面临治疗的挑战，保持积极的心态对康复非常重要。培养乐观的态度有助于应对治疗中的困难。  6. **艺术和娱乐活动：** 参与喜欢的艺术和娱乐活动，如绘画、音乐、阅读等，可以分散注意力，减轻紧张情绪。  7. **定期锻炼：** 适度的运动有助于释放身体内的压力和焦虑，提升整体的心理健康。  8. **保持健康生活方式：** 健康的饮食和足够的睡眠对身体和心理的恢复都是至关重要的。  9. **与医疗团队沟通：** 与医疗团队保持良好的沟通，及时分享任何心理或情绪上的困扰。医疗团队可以提供进一步的支持和资源。  10. **制定合理目标：** 制定实际而合理的目标，逐步实现，有助于保持对治疗过程的掌控感和积极性。  在整个治疗过程中，平衡身体和心理健康的关系是至关重要的，因此，采取综合性的方法来关注整体的健康是推荐的做法。 | similar | 在治疗乳腺癌的过程中，管理心理和情绪健康问题是至关重要的。这不仅关乎患者的生活质量，还直接影响治疗的效果。以下是一些建议，帮助患者更好地应对这一挑战：  认识并接受情绪变化：乳腺癌的诊断和治疗往往伴随着强烈的情绪反应，如恐惧、焦虑、抑郁等。患者首先需要认识到这些情绪是正常的反应，并学会接受它们，而不是与之抗争。 寻求专业心理支持：心理咨询师或心理医生能够提供专业的心理支持，帮助患者处理复杂的情绪问题。他们可以通过认知行为疗法、心理动力学治疗等方法，帮助患者调整心态，增强自信。 加入支持团体：参加乳腺癌患者的支持团体，可以与有相似经历的人分享经验、互相鼓励。这种团体支持往往能够提供独特的情感慰藉和实用建议。 保持积极心态：尽管面临困境，但保持积极的心态对于恢复健康至关重要。患者可以通过冥想、瑜伽、正念练习等方法，培养积极的心态，提高自我调适能力。 与家人和朋友沟通：与家人和朋友的沟通是缓解心理压力的有效途径。患者应该坦诚地表达自己的感受和需要，让亲人成为自己的坚强后盾。 保持健康的生活方式：均衡的饮食、适量的运动、充足的睡眠等健康生活习惯，不仅有助于身体康复，还能改善心理状态。此外，避免过度劳累和压力过大也是关键。 设定可实现的目标：在治疗过程中，设定一些短期和可实现的目标，如完成一次化疗、参加一次社交活动等，可以让患者感到有所成就，增强自信心。 学会放松和减压：学习一些放松技巧，如深呼吸、渐进性肌肉松弛等，有助于缓解紧张和焦虑。此外，听音乐、阅读、绘画等兴趣爱好也能帮助患者放松心情。 总之，在治疗乳腺癌的过程中，管理心理和情绪健康问题是一个持续的过程。患者需要积极寻求帮助、调整心态、保持健康的生活方式，并与家人和朋友保持紧密沟通。通过这些努力，患者不仅能够更好地应对治疗带来的挑战，还能提高生活质量，为康复创造有利条件。 | similar |  |
| 11. What lifestyles contribute to the recovery of breast cancer patients? | Sure thing! Living a healthy lifestyle can significantly contribute to the recovery of breast cancer patients. Here are some key aspects to focus on:  1. **Nutritious Diet:**  - A well-balanced diet is crucial for overall health and can support the body during cancer treatment. Include a variety of fruits, vegetables, whole grains, lean proteins, and healthy fats in your meals.  - Adequate nutrition helps the body maintain strength and supports the immune system.  2. **Regular Exercise:**  - Engage in regular physical activity as per your doctor's recommendations. Exercise helps improve mood, reduce fatigue, and maintain a healthy weight.  - Include a mix of aerobic exercises, strength training, and flexibility exercises.  3. **Stress Management:**  - Practice stress-reducing techniques such as meditation, deep breathing exercises, yoga, or mindfulness. Chronic stress can negatively impact the immune system and overall well-being.  4. **Adequate Sleep:**  - Ensure you get enough quality sleep each night. Sleep is essential for the body to repair and regenerate cells, crucial during cancer recovery.  5. **Limit Alcohol Consumption:**  - If you consume alcohol, do so in moderation. Excessive alcohol intake has been linked to an increased risk of breast cancer recurrence.  6. **Social Support:**  - Maintain a strong support system. Friends and family can provide emotional support, assist with daily tasks, and make the recovery process more manageable.  7. **Regular Medical Check-ups:**  - Attend all scheduled medical appointments and screenings. Regular check-ups help in early detection of any potential issues.  8. **Follow Medical Recommendations:**  - Adhere to the prescribed treatment plan, medications, and follow-up care as recommended by your healthcare team.  9. **Quit Smoking:**  - If you smoke, consider quitting. Smoking is associated with various health issues, and quitting can improve overall health and recovery.  10. **Educate Yourself:**  - Understand your condition, treatment options, and potential side effects. Being informed empowers you to actively participate in your recovery.  Remember, every individual is unique, and recommendations may vary based on specific circumstances. It's crucial to consult with your healthcare team to develop a personalized plan that suits your needs and supports your recovery journey. | similar | 你好，了解到乳腺癌的康复过程对患者来说至关重要。以下是一些建议，可以帮助乳腺癌患者更好地康复：  1. **饮食健康：** 采用健康均衡的饮食是乳腺癌康复的关键。增加蔬菜、水果、全谷物和健康蛋白质的摄入，同时限制饱和脂肪和加工食品。  2. **适度运动：** 规律的适度运动对于乳腺癌患者的康复非常重要。这包括散步、游泳、瑜伽等，有助于提高身体的整体健康状况。  3. **保持健康体重：** 维持健康的体重有助于减少癌症的复发风险。医生可能会提供合适的体重管理建议。  4. **定期随访和检测：** 定期的随访和检测是确保康复的重要环节。这包括定期的医学检查、乳房自检和其他必要的检查项目。  5. **心理健康：** 康复不仅仅涉及身体，心理健康同样重要。患者可能会考虑心理支持、心理治疗或参与癌症康复支持小组。  6. **药物治疗和遵医嘱：** 如有需要，患者应该按照医生的建议进行药物治疗。遵守医嘱，按时服药，是康复过程中至关重要的一部分。  7. **避免致癌物质：** 尽量避免接触可能增加癌症风险的物质，如化学物质、放射线等。  8. **定期乳腺筛查：** 即使康复后，定期进行乳腺筛查也是防止疾病复发的有效手段。  请注意，以上建议应该根据每个患者的具体情况进行个性化调整，最好在医生的指导下制定和执行康复计划。 | similar | 作为乳腺癌专家，我深知乳腺癌患者在康复期所面临的挑战。康复不仅仅是身体上的恢复，还包括心理和社会层面的适应。以下是一些有助于乳腺癌患者康复的生活方式建议：  保持健康的饮食习惯：均衡的饮食对康复至关重要。建议患者增加蔬菜、水果和全谷物的摄入量，同时减少高脂肪、高糖和高盐食物的摄入。此外，适量摄入富含优质蛋白质的食物，如鱼、瘦肉和豆类，有助于身体的修复和免疫力的提升。 规律进行体育锻炼：适度的体育锻炼可以改善心肺功能，增强肌肉力量，提高身体耐力和免疫力。患者可以选择适合自己身体状况的运动方式，如散步、慢跑、瑜伽或游泳等，并逐渐增加运动强度和时间。 保持良好的心理状态：乳腺癌的诊断和治疗过程可能会给患者带来很大的心理压力。因此，寻求心理支持、进行心理咨询或参加心理康复小组等活动，有助于患者调整心态，增强自信，更好地面对康复过程中的挑战。 定期复查和随访：遵循医生的建议，定期进行复查和随访是确保康复顺利进行的重要环节。通过定期检查，可以及时发现并处理任何可能的复发或并发症。 避免或减少不良习惯：吸烟和饮酒等不良习惯可能对乳腺癌患者的康复产生负面影响。因此，患者应尽量戒烟限酒，以改善整体健康状况。 请注意，以上建议仅供参考，具体的康复计划应根据患者的具体情况和医生的建议来制定。每个人的康复过程都是独特的，因此与医疗团队保持密切沟通，共同制定个性化的康复方案是至关重要的。 | similar |  |
| 12. What daily care is required for a subcutaneously implanted infusion port? | Daily care for a subcutaneously implanted infusion port is essential to maintain its functionality and prevent complications. Here's a detailed overview:  1. **Keep the Area Clean**:   - Wash your hands thoroughly with soap and water before touching the port site.  - Clean the skin around the port with an antiseptic solution or sterile saline solution. Avoid using alcohol as it can cause drying and irritation.  2. **Flush the Port Regularly**:  - Flushing the port with saline or heparin solution helps prevent blood clots and keeps the catheter clear.  - The frequency of flushing depends on your healthcare provider's instructions, but it's typically done daily or weekly.  3. **Monitor for Signs of Infection**:  - Keep an eye out for redness, swelling, warmth, or pain at the port site, as these could indicate an infection.  - Report any signs of infection or unusual symptoms to your healthcare provider immediately.  4. **Protect the Port**:  - Avoid activities that could damage the port, such as heavy lifting or wearing tight clothing that rubs against the port site.  - Use a protective cover or pad over the port site if participating in contact sports or activities that may increase the risk of trauma.  5. **Maintain Hygiene During Access**:  - Ensure that anyone accessing the port follows proper sterile technique to minimize the risk of introducing bacteria into the port.  - Sterilize the access site with an antiseptic solution before inserting needles or administering medications.  6. **Stay Hydrated**:  - Adequate hydration helps maintain good blood flow, which can reduce the risk of complications such as clotting.  7. **Regular Follow-up**:  - Attend scheduled appointments with your healthcare provider for port maintenance, flushing, and monitoring.  8. **Report Any Issues Promptly**:  - If you experience any problems with the port, such as difficulty flushing, pain, or swelling, contact your healthcare provider right away.  By following these daily care practices, you can help ensure the long-term functionality and effectiveness of your subcutaneously implanted infusion port while minimizing the risk of complications. | similar | 对于皮下埋置的输液港，需要进行以下日常护理：  1. **保持清洁**：每天用清水或医生建议的皂液轻柔清洁输液港周围的皮肤。避免使用酒精或碘酒等刺激性物质清洁，因为这些物质可能刺激皮肤或损坏港口。  2. **定期注射生理盐水**：如果输液港未被使用，建议每4周注射一次生理盐水以保持通畅。这有助于防止港口内结痂或堵塞。  3. **定期使用**：如果输液港处于使用状态，请确保按照医生或护士的建议使用。使用后，及时清洁港口并注意观察是否有异常。  4. **避免外力撞击**：避免穿戴过紧的衣物或背包，以免对输液港施加压力或引起不适。  5. **避免潜在感染源**：避免在输液港周围使用粉末状化妆品或香水，以减少潜在的感染风险。  6. **定期检查**：定期由医疗专业人员进行输液港的检查，确保其功能正常并及时发现并处理任何问题。  7. **遵医嘱用药**：按照医生的建议使用抗生素或抗菌药物，以预防感染或处理已发生的感染。  通过遵循这些日常护理措施，可以最大程度地减少皮下埋置的输液港的感染和并发症的风险，并确保其长期有效使用。 | similar | 作为乳腺癌专家，对于与乳腺癌治疗相关的医疗设备和护理方法有着深入了解。其中，输液港是一种常用于化疗等长期输液治疗的医疗设备。以下是针对皮下埋置的输液港的日常护理建议：  保持皮肤干燥和清洁：这是防止感染的关键。建议患者在洗澡时避免用力擦洗输液港周围的皮肤，并在洗澡后用干净的毛巾轻轻擦干。 避免剧烈活动：剧烈活动可能会导致输液港移位或损坏。因此，建议患者在活动时要适度，并避免对埋置部位施加过大的压力。 定期检查：患者应定期检查输液港的位置和功能，以确保其正常工作。如果发现输液港有任何异常，如红肿、疼痛、流出液体等，应立即联系医生进行处理。 避免使用锐利物品：患者应避免在输液港周围使用剪刀、针等锐利物品，以防止意外损伤输液港或周围的皮肤。 此外，对于乳腺癌患者，化疗是常见的治疗方法之一，而输液港则是化疗过程中的重要辅助设备。因此，患者还需要了解化疗的相关知识，如化疗的副作用、如何应对化疗反应等，以便更好地配合治疗。  总的来说，皮下埋置的输液港的日常护理主要包括保持皮肤干燥和清洁、避免剧烈活动、定期检查以及避免使用锐利物品等。同时，患者还需要了解与输液港相关的治疗知识，以便更好地应对治疗过程中的各种情况。 |  |  |
| 13. Can breast cancer patients have normal fertility after discharge? | Absolutely, breast cancer and its treatments can have various effects on fertility, and the impact differs from person to person. Let me break it down for you.  1. **Chemotherapy and Fertility:**  - Chemotherapy, a common treatment for breast cancer, may affect fertility. Some chemotherapy drugs can damage the eggs in a woman's ovaries, potentially leading to temporary or permanent infertility.  - However, the extent of impact varies based on factors such as the specific drugs used, the woman's age, and her overall health.  2. **Hormone Therapy and Fertility:**  - Hormone therapy, another breast cancer treatment, might also influence fertility. It can induce temporary or permanent menopause, affecting a woman's ability to conceive.  3. **Surgery and Fertility:**  - Surgical procedures like mastectomy (removal of the breast) or oophorectomy (removal of ovaries) may not directly impact fertility, but they can have psychological and emotional effects on a person's perception of their body and fertility.  4. **Fertility Preservation:**  - For those concerned about fertility, discussions about fertility preservation options should happen before starting cancer treatment. This may include egg or embryo freezing.  5. **Post-Treatment Fertility:**  - After completing breast cancer treatment, some women regain normal fertility, while others may experience challenges. The duration it takes for fertility to return varies.  6. **Consultation with Specialists:**  - It's crucial for breast cancer patients to have open and detailed discussions with their oncologists and fertility specialists. These professionals can provide personalized information based on the individual's health status, treatment plan, and fertility concerns.  7. **Supportive Care:**  - Emotional and psychological support is vital. Coping with the diagnosis and treatment of breast cancer can be challenging, and addressing fertility concerns is an important aspect of holistic care.  In summary, the impact of breast cancer on fertility is multifaceted. Each case is unique, and consultation with healthcare providers, including fertility specialists, is essential to address individual concerns and explore available options. | similar | 当乳腺癌患者出院后是否能正常生育取决于多个因素，包括疾病的特定情况、治疗方案以及个体患者的生理状况。  首先，乳腺癌的治疗方法通常包括手术、放疗、化疗和荷尔蒙疗法等。这些治疗可能对生育能力产生影响。例如，化疗和荷尔蒙疗法可能对卵巢功能造成暂时或永久性的影响，从而影响生育。  其次，手术和放疗可能会对身体结构和器官产生影响，这可能会影响怀孕和分娩的过程。特定的手术类型和治疗方案会对患者的身体产生不同的影响。  最后，个体差异也是一个重要因素。一些女性在治疗后仍然能够正常怀孕和生育，而另一些可能会面临生育困难或不适宜怀孕的情况。  如果一个乳腺癌患者考虑在治疗后生育，她应该在治疗前咨询医生。医生可以评估她的个体情况，讨论潜在的影响，并提供有关如何最好地管理生育计划的建议。  总体而言，乳腺癌患者出院后能否正常生育是一个复杂的问题，需要考虑多个因素。因此，建议患者在做出决定之前与医生进行深入的讨论和咨询。 | similar | 作为乳腺癌专家，对于乳腺癌患者的生育问题有深入的研究和丰富的临床经验。首先，可以明确地告诉大家，乳腺癌患者出院后仍然有可能正常生育。  乳腺癌的治疗方式多样，包括手术、化疗、放疗、内分泌治疗和靶向治疗等。这些治疗方法在一定程度上可能会影响患者的生育能力，尤其是化疗和放疗，它们可能会导致卵巢功能的暂时或永久性损伤。但是，这并不意味着乳腺癌患者就完全失去了生育的机会。  卵巢功能保护：在治疗前，医生会根据患者的年龄、病情和治疗方案等因素，评估卵巢功能的风险，并尽可能地采取保护措施，如使用促性腺激素释放激素类似物等药物，来降低卵巢受损的风险。 生育力保存技术：对于有生育需求的患者，可以考虑采用生育力保存技术，如卵子冷冻、胚胎冷冻等，来保留生育能力。这些技术可以在治疗前或治疗期间进行，以便在未来合适的时候使用。 另外，乳腺癌患者出院后的生育决策应该是个体化的。在决定是否要生育之前，患者应该与医生进行充分的沟通，了解自身的病情、治疗情况和生育风险，并进行全面的评估。医生会根据患者的具体情况，提供个性化的建议和指导。  同时，乳腺癌患者在出院后还需要进行长期的随访和监测，以确保身体的健康和生育的安全。在随访过程中，医生会密切关注患者的病情变化和生育情况，并根据需要调整治疗方案和生育指导。  总之，乳腺癌患者出院后仍然有可能正常生育。但是，在做出生育决策之前，患者需要进行全面的评估和与医生的充分沟通，以确保自身的健康和生育的安全。同时，长期的随访和监测也是必不可少的。 | similar |  |
| 14. What is the risk of recurrence and the corresponding monitoring methods after breast cancer treatment? | Certainly! I'll provide a detailed response on the risk of recurrence and the corresponding monitoring methods after breast cancer treatment.  **Risk of Recurrence:** The risk of breast cancer recurrence varies depending on several factors, including the stage at diagnosis, tumor characteristics, treatment received, and individual patient factors. It's important to note that no two cases are exactly alike. Generally, the risk of recurrence is higher in the first few years after initial treatment, but it can persist for many years.  - **Stage of Cancer:** The stage at which breast cancer was initially diagnosed is a crucial factor. Generally, the earlier the stage, the lower the risk of recurrence.  - **Tumor Characteristics:** The biology of the tumor, such as hormone receptor status (estrogen and progesterone receptors) and HER2/neu status, influences the risk. Hormone receptor-positive tumors may have a different recurrence pattern compared to hormone receptor-negative ones.  - **Treatment Received:** The type and success of the initial treatment play a significant role. Patients who undergo surgery, chemotherapy, radiation therapy, and hormonal therapy may have different recurrence risks based on the effectiveness of each treatment.  **Monitoring Methods:** After completing breast cancer treatment, ongoing monitoring is crucial to detect any signs of recurrence or new cancer development. Monitoring methods may include:  - **Regular Follow-up Visits:** Patients typically have regular follow-up appointments with their oncologist. During these visits, the medical team assesses the patient's overall health, reviews any symptoms or concerns, and may perform physical examinations.  - **Imaging Studies:** Depending on the patient's history and initial treatment, imaging studies such as mammograms, ultrasounds, or MRIs may be used for surveillance. These imaging studies help detect any abnormalities in the breast tissue.  - **Blood Tests:** Tumor markers in the blood, such as CA 15-3 and CA 27.29, may be monitored. However, these markers are not definitive for all types of breast cancer and are used more as supplementary tools.  - **Biopsies:** In some cases, if there are suspicious findings on imaging or symptoms, a biopsy may be recommended to confirm whether the changes are due to cancer recurrence.  - **Genetic Testing:** For individuals with a strong family history or known genetic mutations (e.g., BRCA1, BRCA2), genetic testing may be recommended to assess the risk of a new breast cancer or other cancers.  It's essential for individuals who have undergone breast cancer treatment to have open and regular communication with their healthcare team to discuss any concerns, symptoms, or changes in their health. The frequency and type of monitoring can vary based on individual circumstances, and decisions are often made collaboratively between the patient and their healthcare provider. | similar | 乳腺癌治疗后的复发风险和相应的监测方法是一个复杂而且关键的话题。复发风险受多种因素影响，包括患者的病理特征、治疗方案、分子生物学特征以及遗传因素等。以下是一些可能影响复发风险的因素和一些常见的监测方法：  ### 复发风险因素：  1. **病理特征：** 包括肿瘤大小、分级、浸润深度、淋巴结转移情况等。   2. **治疗方案：** 不同的治疗方案，如手术、放疗、化疗、内分泌治疗、靶向治疗等，对复发风险有不同的影响。  3. **分子生物学特征：** 比如雌激素受体（ER）、孕激素受体（PR）、人类表皮生长因子受体2（HER2）等的表达情况，以及基因组学的变异情况。  4. **遗传因素：** 家族史中是否有乳腺癌等遗传疾病。  ### 监测方法：  1. **临床检查：** 定期由医生进行的乳腺体检和淋巴结检查，以便及早发现任何异常。  2. **影像学检查：** 如乳腺X线摄影（乳腺X光）、乳腺超声波、磁共振成像（MRI）等，可用于检测复发或转移。  3. **血液标志物检测：** 监测血液中特定标志物（如CA 15-3、CA 27.29等）的变化，有助于判断疾病复发或进展。  4. **组织学检查：** 可以通过组织学检查（如活检）来确定是否有复发并进行分子生物学特征分析。  5. **全身检查：** 包括骨扫描、PET-CT等全身性检查，用于排除或确定远处转移。  6. **基因检测：** 对一些高风险群体，如有BRCA1和BRCA2基因突变的患者，可以进行基因检测以评估复发风险。  综合利用以上多种监测方法，可以帮助医生及时发现乳腺癌的复发或转移，从而采取适当的治疗措施。但需要注意的是，监测方法的选择应根据患者的具体情况和医生的建议进行。 | similar | 乳腺癌治疗后的复发风险确实存在，但具体的风险程度因多种因素而异，包括肿瘤的生物学特性（如肿瘤大小、分级、激素受体状态、HER2状态等）、治疗的及时性和有效性、患者的整体健康状况等。为了更准确地评估个体的复发风险，通常会使用统计模型或工具，如Oncotype DX等，这些工具可以基于肿瘤的分子特征来预测复发风险。  在监测复发方面，临床医生和患者应该密切合作，制定个性化的监测计划。这通常包括定期的体检、血液检查和影像学检查。具体的监测频率和方法可能会根据患者的具体情况和医生的建议略有不同。  体检：医生通过触摸患者的乳房和腋窝区域来检查是否有异常的肿块或淋巴结肿大。  血液检查：某些肿瘤标志物（如CA 15-3和CA 27.29）的水平可能会在乳腺癌复发时升高，因此定期检测这些标志物有助于早期发现复发迹象。  影像学检查：包括乳腺X线摄影（乳腺钼靶）、超声检查、MRI等。这些检查可以帮助医生发现乳房内的异常结构或肿块。对于已经接受过乳腺癌手术的患者，还可能需要进行胸部CT扫描、骨扫描或PET-CT等全身性检查，以评估癌症是否已扩散到其他部位。  需要强调的是，即使患者已经完成了乳腺癌的初始治疗并进入了监测阶段，保持健康的生活方式仍然非常重要。这包括保持适当的体重、进行规律的体育锻炼、避免或限制酒精摄入、遵循健康的饮食习惯等。这些措施不仅有助于降低复发风险，还有助于提高患者的整体生活质量和预后。  总的来说，乳腺癌治疗后的复发风险是存在的，但通过密切的监测和健康的生活方式，可以有效地管理这一风险。患者应与医生保持良好的沟通，并遵循医生的建议进行定期检查和评估。 | similar |  |
| 15. If a breast cancer patient has other chronic illnesses or new health issues that need to be addressed, how should these issues be coordinated with the treatment of breast cancer? | Sure thing! When a breast cancer patient has other chronic illnesses or new health issues, it's crucial to approach their treatment comprehensively. Coordination among different healthcare providers is key to ensuring that the patient receives optimal care for both breast cancer and other health conditions. Here's a detailed breakdown:  1. **Multidisciplinary Approach:**  - **Breast Cancer Team:** The primary oncologist overseeing the breast cancer treatment should be informed about the patient's other health issues. They can collaborate with specialists such as cardiologists, pulmonologists, or other relevant healthcare professionals.  - **Regular Communication:** Constant communication among the various healthcare providers is essential. This includes sharing medical records, test results, and treatment plans.  2. **Prioritization of Health Issues:**  - **Assessment of Severity:** Evaluate the urgency and severity of both breast cancer and other health issues. This helps in determining which condition needs immediate attention and which can be managed concurrently.  3. **Treatment Plan Adjustment:**  - **Tailored Approach:** Develop a treatment plan that takes into account the patient's overall health status. Treatment modalities for breast cancer might need to be adjusted based on the presence of other health issues.  - **Medication Interactions:** Be mindful of potential interactions between breast cancer medications and those prescribed for other conditions. Adjustments may be necessary to prevent adverse effects.  4. **Regular Monitoring:**  - **Ongoing Assessment:** Regularly monitor the patient's overall health, both during and after breast cancer treatment. This helps in identifying any changes or developments in other health issues that may require adjustments to the treatment plan.  5. **Supportive Care:**  - **Palliative Care:** For patients with advanced breast cancer and other chronic illnesses, palliative care becomes crucial. This specialized medical care focuses on providing relief from the symptoms and stress of serious illnesses.  6. **Patient-Centered Care:**  - **Informed Decision-Making:** Involve the patient in the decision-making process. Discuss the potential benefits and risks of different treatment options, considering their overall health status and preferences.  7. **Psychosocial Support:**  - **Mental Health Considerations:** Recognize the impact of both breast cancer and other chronic illnesses on the patient's mental health. Provide access to counseling or support groups to address emotional and psychological well-being.  8. **Continuity of Care:**  - **Post-Treatment Follow-up:** After breast cancer treatment, ensure a seamless transition to post-treatment follow-up care. This includes ongoing monitoring for cancer recurrence and managing other chronic health conditions.  In summary, a collaborative and patient-centered approach, with open communication among healthcare providers, is essential when managing breast cancer in conjunction with other chronic illnesses. This ensures that the patient receives comprehensive and coordinated care tailored to their specific needs and circumstances. | similar | 你好啊！首先，我要强调的是，乳腺癌治疗的成功很大程度上依赖于一个综合性的医疗团队，包括乳腺癌专家、内科医生以及其他专业医生。当患者同时患有其他慢性疾病或者出现新的健康问题时，协调各方面的治疗变得尤为重要。  1. **医疗团队的协作：** 不同专业的医生之间需要密切合作，确保各种治疗方案不会相互干扰。这可能需要定期的团队会议，以便共享患者的信息和制定综合治疗计划。  2. **治疗优先级：** 在协调治疗时，医生需要根据疾病的紧急程度和治疗的优先级来制定计划。有时候，可能需要暂停乳腺癌治疗以处理其他急迫的健康问题。  3. **个体化治疗计划：** 每位患者的状况都是独特的，因此治疗计划应该根据个体的整体健康状况进行调整。这可能涉及到调整药物剂量、选择合适的治疗时机等。  4. **全面评估：** 在制定治疗计划时，医生需要对患者进行全面的评估，考虑到乳腺癌治疗可能对其他慢性疾病造成的影响。这包括药物相互作用、治疗的副作用等方面。  5. **患者教育：** 了解自己的病情对患者至关重要。医生需要花时间向患者解释治疗计划、可能的风险以及如何应对其他慢性疾病的变化。  总体而言，协调乳腺癌治疗与其他健康问题的关键在于个体化、全面评估和医疗团队的协作。这样可以最大程度地确保患者获得全面而有效的治疗，同时最小化不同治疗之间的干扰。如果有具体的问题或者需要更详细的信息，请随时告诉我。 | similar | 作为乳腺癌专家，我深知乳腺癌的治疗可能会受到其他慢性疾病或新的健康问题的影响。因此，协调这些问题与乳腺癌的治疗至关重要。以下是一些建议：  首先，当乳腺癌患者有其他慢性疾病时，治疗团队应该全面了解患者的病史和当前健康状况。这有助于评估慢性疾病对乳腺癌治疗的可能影响，并制定相应的治疗计划。  其次，对于需要长期管理的慢性疾病，如糖尿病、高血压或心脏病，治疗团队应与患者的其他医生密切合作。这种多学科协作可以确保患者在接受乳腺癌治疗的同时，也能得到适当的慢性病管理。例如，糖尿病患者可能需要调整胰岛素或口服药物的剂量，以保持血糖稳定；心脏病患者可能需要额外的监测来调整其心脏药物。  此外，如果患者在乳腺癌治疗期间出现新的健康问题，如感染、骨折或其他急性病症，治疗团队应立即评估这些情况对乳腺癌治疗的影响。在某些情况下，可能需要暂停或调整乳腺癌治疗方案，以优先处理急性病症。一旦急性病症得到控制，治疗团队应重新评估患者的状况，并恢复或调整乳腺癌治疗方案。  同时，患者的心理和社会支持也不容忽视。乳腺癌及其治疗可能会给患者带来很大的心理压力。其他慢性疾病或新的健康问题可能会进一步加重这种负担。因此，提供心理支持、情绪疏导和社会资源等信息对患者来说至关重要。  最后，在整个治疗过程中，持续监测和评估患者的健康状况是至关重要的。这有助于及时发现并处理任何可能出现的问题，确保患者能够获得最佳的治疗效果和生活质量。  总之，协调乳腺癌治疗与其他慢性疾病或新的健康问题需要一个综合、多学科的治疗团队。通过全面了解患者的病史、与其他医生合作、及时调整治疗方案以及提供心理和社会支持，我们可以帮助患者更好地应对这些挑战，提高治疗效果和生活质量。 | similar |  |
| **Expert Questionnaires** |  |  |  |  |  |  | |
| 1. What are the screening methods for breast cancer? | Absolutely, let's delve into breast cancer screening methods. It's crucial to note that screening is essential for early detection, significantly improving treatment outcomes.  1. **Mammography:**  - **Description:** X-ray imaging of the breast.  - **Frequency:** Generally recommended annually for women over 40.  - **Effectiveness:** High sensitivity in detecting abnormalities, but false positives can occur.  2. **Clinical Breast Exam (CBE):**  - **Description:** Physical examination by a healthcare professional.  - **Frequency:** It was often done annually, but recommendations have evolved.  - **Effectiveness:** Limited; often used in conjunction with mammography.  3. **Breast Self-Exam (BSE):**  - **Description:** Self-examination by the individual.  - **Frequency:** No longer widely recommended due to concerns about reliability.  - **Effectiveness:** Limited; may lead to unnecessary anxiety or missed detection.  4. **Breast Ultrasound:**  - **Description:** Sound waves create images of the breast.  - **Use:** As a supplemental tool, often for further evaluation after mammography.  - **Effectiveness:** Useful for differentiating between solid and fluid-filled masses.  5. **MRI (Magnetic Resonance Imaging):**  - **Description:** Powerful magnets and radio waves create detailed breast images.  - **Use:** Typically for high-risk individuals or as a follow-up for unclear mammogram results.  - **Effectiveness:** High sensitivity but lower specificity compared to mammography.  6. **Genetic Testing:**  - **Description:** Identifying specific gene mutations (e.g., BRCA1, BRCA2).  - **Use:** For individuals with a family history of breast cancer.  - **Effectiveness:** Guides preventive measures and treatment decisions.  7. **3D Mammography (Tomosynthesis):**  - **Description:** Multiple X-ray images taken from different angles, creating a 3D picture.  - **Use:** Offers improved clarity, reducing false positives.  - **Effectiveness:** Shows promise in enhancing detection rates.  8. **Breast Biopsy:**  - **Description:** Removal of a sample for lab examination.  - **Use:** Definitive diagnosis following suspicious findings in other screenings.  - **Effectiveness:** Confirms whether a detected abnormality is cancerous.  It's essential to consult with healthcare professionals to determine the most appropriate screening plan based on individual risk factors and age. Regular screenings, especially mammography, remain the cornerstone for early detection of breast cancer. | similar | 乳腺癌的筛查手段主要包括以下几种：  1. **乳房自我检查（Breast Self-Examination，BSE）**：这是一种简单的方法，可以在家中进行。通过触摸、观察乳房，寻找任何异常的变化，如肿块、皮肤变化等。虽然BSE有助于发现一些异常，但它并不是一种完全可靠的筛查方法。  2. **临床乳腺检查（Clinical Breast Examination，CBE）**：由医生或护士进行的乳房检查。在医疗机构进行，通常与其他筛查手段结合使用。  3. **乳腺X线摄影术（Mammography）**：是目前最常用的乳腺癌筛查方法之一。它利用X射线对乳房进行拍摄，以检测乳腺组织中的任何异常，如肿块或钙化。早期乳腺癌通常在乳房X线摄影术中可见，因此对于乳腺癌的早期诊断至关重要。  4. **乳腺超声检查（Breast Ultrasound）**：超声波技术可以帮助医生观察乳腺组织的内部结构，包括对肿块的评估。它通常与乳腺X线摄影术一起使用，可以帮助鉴别囊性肿块和实质性肿块。  5. **乳腺磁共振成像（Breast Magnetic Resonance Imaging，MRI）**：对于高风险人群，如家族中有乳腺癌家族史的人，乳腺MRI可能被用作筛查工具。MRI可以提供更为详细的图像，有助于识别早期的乳腺癌。  6. **乳腺活检（Biopsy）**：如果在以上筛查手段中发现了异常，医生可能会建议进行乳腺活检，以确认是否存在乳腺癌。活检是通过取样乳腺组织进行实验室检测来确定组织是否为癌细胞。  这些筛查方法通常会根据个体的年龄、乳腺癌风险因素以及医生的建议进行选择和组合使用，以达到最佳的筛查效果。及早发现乳腺癌有助于提高治疗成功率和生存率。 | similar | 乳腺癌的筛查是早期发现乳腺癌、提高治愈率、降低死亡率的重要手段。以下是目前常用的乳腺癌筛查手段：  乳腺X线摄影（乳腺钼靶）：这是最常用的乳腺癌筛查方法之一，通过X线照射乳腺组织，可以检测到乳腺内的微小钙化、肿块等异常结构，有助于早期发现乳腺癌。乳腺钼靶检查具有操作简便、辐射剂量小、价格相对较低等优点，适用于大规模的人群筛查。  乳腺超声检查：超声检查利用声波在乳腺组织中的传播和反射特性，可以显示乳腺内的肿块、囊肿等结构，对于判断肿块的性质（囊性还是实性）以及肿块的血流情况有很大帮助。乳腺超声检查具有无辐射、无创伤、可重复性好等优点，尤其适用于年轻女性和乳腺组织较致密的人群。  乳腺磁共振成像（MRI）：MRI是一种较新的乳腺癌筛查方法，具有极高的软组织分辨率，可以清晰地显示乳腺内的肿块、血管等结构。乳腺MRI检查对于发现早期乳腺癌、评估肿瘤大小、判断肿瘤浸润范围等方面具有很大优势。然而，乳腺MRI检查价格较高，操作相对复杂，且需要注射造影剂，因此一般不作为首选的筛查方法。  临床乳腺检查：这是一种简单易行的乳腺癌筛查方法，通过医生对乳腺进行触诊，可以发现乳腺内的肿块、增厚等异常表现。然而，临床乳腺检查的准确性受到医生经验和技能的影响，因此一般作为其他筛查方法的补充。  总之，乳腺癌的筛查手段多种多样，各种方法各有优缺点。在实际应用中，医生会根据患者的年龄、乳腺组织情况、家族史等因素综合选择合适的筛查方法。同时，定期进行乳腺癌筛查是预防乳腺癌、提高治愈率的关键。 | 乳腺癌的筛查是早期发现乳腺癌、提高治愈率、降低死亡率的重要手段。以下是目前常用的乳腺癌筛查手段：  乳腺X线摄影（乳腺钼靶照相）：这是目前诊断乳腺疾病的首选和最简便、最可靠的无创性检测手段。它的痛苦相对较小，简便易行，且分辨率高，重复性好，留取的图像可供前后对比，不受年龄、体形的限制，已作为常规的检查。不过，乳腺X线摄影检查对钙化灶较敏感，但对于评估肿块性质方面有一定局限性。 乳腺彩超：这是一种经济、简便、无创伤、无痛苦的检查方法，对年轻女性，尤其是妊娠、哺乳期妇女更为适宜，但对微小钙化的检测敏感性不如乳腺照相。两种方法可以互补，提高乳腺癌的早诊率。 乳腺触诊：这是最简单也最直接的方法，由专业医生进行触诊，可以检查乳腺内的肿块或异常区域。但这种方法依赖于医生的经验和技能，且对于深层或较小的病变可能无法准确检测。 乳腺MRI检查：这是近年来新兴的检查方法，对于发现早期乳腺癌具有一定的优势。然而，由于其价格昂贵且操作复杂，一般不作为首选检查方法。 乳腺穿刺活检：当乳腺X线摄影或彩超检查发现异常时，医生可能会建议进行乳腺穿刺活检以明确诊断。这是确诊乳腺癌的“金标准”。 需要注意的是，虽然以上方法可以用于乳腺癌的筛查，但并不能完全保证早期发现所有的乳腺癌。因此，建议女性定期进行乳腺检查，并结合自身情况和医生建议选择合适的筛查方法。同时，保持良好的生活习惯和心态也是预防乳腺癌的重要措施。 | |
| 2. What imaging and biomarkers will you use to assist in the preoperative diagnosis of breast cancer? | Hey there! When it comes to preoperative diagnosis of breast cancer, we typically employ a combination of imaging techniques and biomarkers to gather comprehensive information. Let's delve into the specifics:  **1. Imaging Techniques:**  - **Mammography:** This is a standard screening tool for breast cancer. It uses X-rays to create detailed images of the breast tissue. It's especially effective in detecting early signs of cancer.    - **Ultrasound:** Often used alongside mammography, ultrasound helps differentiate between solid masses and fluid-filled cysts. It's particularly useful for further evaluation of abnormalities detected on mammograms.    - **MRI (Magnetic Resonance Imaging):** This imaging technique provides detailed pictures of breast tissue, helping in the assessment of tumor size and extent. It's particularly valuable in high-risk cases or when additional information is needed.   - **CT (Computed Tomography) Scan or PET (Positron Emission Tomography) Scan:** In some cases, these scans may be used to determine whether cancer has spread to other parts of the body.  **2. Biomarkers:**  - **HER2 Status:** Human Epidermal Growth Factor Receptor 2 (HER2) is a protein that can promote the growth of cancer cells. HER2-positive breast cancers may respond to targeted therapies.    - **Estrogen and Progesterone Receptor Status:** These receptors indicate whether the cancer is sensitive to hormones. Hormone receptor-positive cancers may respond to hormone therapy.    - **BRCA Gene Mutations:** Genetic testing for BRCA1 and BRCA2 mutations can identify individuals at higher risk of developing breast cancer. It may influence treatment decisions.   - **Ki-67:** This protein is associated with cell proliferation. Higher levels may indicate a more aggressive tumor.  **3. Biopsy:**  - **Fine Needle Aspiration (FNA) or Core Needle Biopsy:** Tissue samples are taken for laboratory analysis, confirming the presence of cancer and providing details about its type and characteristics.  These tools and markers help oncologists tailor treatment plans and make informed decisions about surgery and other interventions. It's important to note that the choice of diagnostic methods may vary based on individual cases and specific clinical indications. Always consult with a healthcare professional for personalized advice. If you have more questions or need further clarification, feel free to ask! | similar | 在乳腺癌术前诊断阶段，为了进行全面的评估，医生通常会采用多种影像学和生物标志物进行辅助诊断。以下是一些常见的方法：  1. **乳腺X射线摄影（乳腺X光）：** 也称为乳腺钼靶，这是一种常见的初步筛查方法。它能够显示乳腺组织的结构，并帮助检测是否存在异常，如肿块或钙化。  2. **乳腺超声：** 超声波可以用于进一步评估乳腺组织。它在检测囊性和实质性肿块方面比X光更为敏感，可帮助医生确定肿块的性质。  3. **磁共振成像（MRI）：** 对于一些高风险人群或在X光和超声无法提供清晰信息时，MRI可能被用于更详细的图像。它对于评估乳腺组织的某些特征更为敏感。  4. **乳腺活检：** 这是一种直接获取乳腺组织样本的方法，以确定是否存在癌症细胞。有不同类型的活检，包括穿刺活检和手术活检。  5. **乳腺核磁共振弹性成像（MRE）：** 这是一种新兴的技术，结合了MRI和乳腺组织的弹性信息，有助于更准确地识别肿块的性质。  6. **乳腺癌生物标志物：** 检测血液中的生物标志物，如CA 15-3、CA 27.29等，有助于评估乳腺癌的状况。这些标志物在一些情况下可以提供额外的信息，但不能替代影像学检查。  在实际情况中，医生可能会根据患者的具体病情和风险因素来决定使用哪些检查方法。这些影像学和生物标志物的综合运用有助于医生更全面地了解患者的状况，从而制定更合理的治疗方案。 | similar | 在乳腺癌的术前诊断阶段，为了确保诊断的准确性和为后续的治疗提供有力的依据，通常会采用一系列的影像学检查和生物标志物检测。以下是目前常用的方法和手段：  影像学检查： 乳腺X线摄影（钼靶）：这是乳腺癌筛查和诊断的常用方法，尤其对于钙化灶的检出有很高的敏感性。 乳腺超声：超声对于评估肿块的性质、囊实性、边界等有很好的效果，特别是对于年轻女性和致密型乳腺更为适用。 乳腺MRI（磁共振成像）：MRI在乳腺癌的诊断、分期和治疗效果评估中有重要价值，尤其是对于多病灶、多中心性病灶以及浸润范围的评估。 生物标志物检测： 血清肿瘤标志物：如CA15-3、CEA等，这些标志物的升高可能提示乳腺癌的存在，但它们的特异性并不高，通常用于监测治疗效果和复发。 组织生物标志物：通过穿刺活检或手术取得的乳腺组织，可以进行一系列的生物标志物检测，如ER（雌激素受体）、PR（孕激素受体）、HER2（人类表皮生长因子受体2）等。这些标志物的状态对于乳腺癌的分子分型和后续治疗策略的选择至关重要。 在乳腺癌的术前诊断中，这些影像学和生物标志物的检测不是孤立的，而是需要相互结合、综合分析的。通过这些检测，可以更准确地评估患者的病情，为后续的手术和综合治疗提供有力的依据。  请注意，以上信息仅供参考，如有疑虑或症状，请及时就医并遵循专业医生的诊断和治疗建议。 | similar | |
| 3. What are the requirements for the surgical margins in breast-conserving surgery for ductal carcinoma in situ? | Certainly! In breast-conserving surgery (also known as lumpectomy) for ductal carcinoma in situ (DCIS), the goal is to remove the cancerous tissue while preserving as much of the healthy breast tissue as possible. The requirements for surgical margins in this context refer to the extent of normal tissue surrounding the tumor that should be excised to ensure complete removal of the cancer.  The recommended surgical margins for DCIS can vary, and guidelines may be subject to updates. As of my last knowledge update in September 2021, the guidelines suggested clear margins, typically aiming for a margin width of at least 2 millimeters. However, some studies and guidelines might suggest a wider margin, such as 1 cm.  It's crucial to note that the decision on surgical margins can depend on various factors, including the specific characteristics of the DCIS (such as size, grade, and presence of comedonecrosis), as well as the overall health and preferences of the patient.  In recent years, there has been ongoing research and discussion in the medical community about the optimal surgical margins for DCIS. Some studies have suggested that a wider margin might not necessarily lead to better outcomes, and a balance between ensuring complete removal of the cancer and preserving cosmetic and functional aspects of the breast is essential.  It's highly recommended for individuals to consult with their healthcare team, including their breast surgeon and oncologist, to make informed decisions based on the most current and personalized information. They can discuss the specific characteristics of the DCIS, potential risks and benefits of different margin widths, and the overall treatment plan tailored to the individual's situation.  Please keep in mind that medical guidelines and practices may have evolved since my last update, and it's advisable to consult with a healthcare professional for the latest and most accurate information. | In breast-conserving surgery (BCS) for ductal carcinoma in situ (DCIS), the requirements for surgical margins are crucial to ensure effective treatment and reduce the risk of recurrence. DCIS refers to abnormal cells lining the breast ducts, which have not invaded surrounding tissue.  The optimal surgical margins for DCIS have been a topic of debate and have evolved over time based on clinical research and consensus guidelines. As of my last update in September 2021, here are the general recommendations for surgical margins in BCS for DCIS:  1. **Negative Margins**: The primary goal is to achieve negative margins, meaning no cancer cells are present at the edge of the removed tissue. This reduces the risk of cancer recurrence.  2. **Clear Margins**: Historically, clear margins were defined as at least 2 mm of normal tissue around the DCIS lesion. However, more recent studies and guidelines suggest that a smaller margin may be acceptable in certain cases.  3. **Guidelines Variation**: It's important to note that guidelines may vary among different organizations and regions. For example, the National Comprehensive Cancer Network (NCCN) guidelines in the United States may differ slightly from those of other organizations such as the American Society of Breast Surgeons or the European Society of Breast Cancer Specialists (EUSOMA).  4. **Individualized Approach**: The decision regarding surgical margins should be individualized based on various factors, including the size and grade of the DCIS lesion, the presence of multifocality or multicentricity, patient preferences, and the expertise of the surgical team.  5. **Margin Assessment**: Surgeons typically assess margins intraoperatively using techniques such as specimen radiography, frozen section analysis, or imprint cytology. However, final margin status is often determined by postoperative pathology examination of the entire specimen.  6. **Re-excision**: If initial surgical margins are positive (indicating the presence of cancer cells at the edge of the removed tissue), re-excision or additional treatments such as radiation therapy may be recommended to achieve negative margins.  7. **Risk Factors**: Some factors associated with an increased risk of positive margins include larger tumor size, higher nuclear grade, presence of comedonecrosis, and younger age at diagnosis.  8. **Ongoing Research**: Ongoing research aims to further refine the optimal margin width and identify factors that influence the risk of recurrence after BCS for DCIS.  It's essential for patients to have a thorough discussion with their healthcare team regarding the surgical approach, including the rationale for margin decisions and potential implications for their individual case. | 导管原位癌（DCIS）是一种非侵袭性的乳腺癌前病变，通常在乳腺导管内形成异常细胞。对于导管原位癌的保乳手术，手术切缘的要求至关重要，以确保彻底切除异常细胞并降低复发的风险。  1. **负切缘的定义：** 保乳手术的目标是在手术中尽可能地切除所有异常细胞，同时保持健康的组织。切缘是指被切除组织的边缘。负切缘意味着在切除的组织边缘上未检测到任何异常细胞。  2. **距离要求：** 通常，负切缘的定义包括在离异常细胞一定距离的范围内，确保边缘的清晰和安全。这个距离的具体要求可能会因医学标准和实践而有所不同，但通常在2毫米到10毫米之间。  3. **切缘评估的方法：** 在手术过程中，医生会对被切除的组织进行切缘评估，通常通过冰冻切片或后续的病理检查。这有助于立即确定是否需要进一步切除组织以确保切缘为阴性。  4. **影响决策的因素：** 切缘的要求可能会受到多种因素的影响，包括肿瘤的大小、位置、类型，以及患者的整体健康状况。有时，为了达到负切缘的要求，可能需要额外的手术。  5. **术后监测：** 即使手术切缘是负的，患者通常还需要进行定期的随访和监测，以确保没有复发或新的问题出现。这包括乳腺X光、MRI等影像检查。  总的来说，保乳手术对导管原位癌的治疗是一种常见的选择，但术后切缘的评估和要求对于术后的疗效和患者的长期预后非常重要。患者和医生应密切合作，以制定最适合个体情况的治疗计划。 | similar | 作为乳腺癌专家，深知导管原位癌（DCIS）是乳腺癌的一种早期形式，其中异常细胞仅局限于乳腺导管内，并未侵入周围乳腺组织或扩散至身体其他部位。保乳手术（也称为乳房肿瘤切除术）是治疗DCIS的常用方法之一，其目标是切除肿瘤及周围一小部分正常组织，同时保留大部分乳房。  在保乳手术中，手术切缘的状态是一个非常重要的考虑因素。手术切缘是指切除的肿瘤组织与周围正常乳腺组织之间的边界。对于DCIS，手术切缘的要求通常比浸润性乳腺癌更为严格，因为DCIS有时会在乳腺导管内以不连续的方式生长，增加了复发的风险。  一般来说，对于DCIS的保乳手术，医生会努力确保手术切缘是“阴性”的，这意味着在显微镜下检查切缘时，没有发现癌症细胞的迹象。为了实现这一点，医生可能会在手术过程中切除比初步估计更大的区域，并在术后对切除的组织进行详细的病理学检查。  如果手术切缘呈“阳性”（即有癌症细胞残留），则可能需要再次手术以切除更多的组织，直到获得阴性的手术切缘。这是因为阳性的手术切缘与DCIS复发的风险增加有关。  然而，需要注意的是，即使手术切缘是阴性的，也不能完全保证DCIS不会复发。因此，在保乳手术后，患者通常需要接受放射治疗，以进一步降低局部复发的风险。  总之，对于DCIS的保乳手术，手术切缘的要求非常严格，医生会努力确保切缘是阴性的，以降低复发的风险。如需更多信息，建议查阅相关文献或咨询专业医生。 | 作为乳腺癌专家，我深知导管原位癌（DCIS）是乳腺癌的一种早期形态，主要局限于乳腺的导管内，尚未侵犯到周围的正常乳腺组织。保乳手术（乳房肿瘤切除术）是治疗DCIS的常用方法之一，旨在切除肿瘤及其周围一小部分正常组织，同时保留大部分乳房。  在进行保乳手术时，手术切缘的要求是非常关键的，因为切缘的状态直接影响到手术后复发的风险。以下是关于导管原位癌保乳手术对手术切缘的一般要求：  切缘阴性：这是最基本也是最重要的要求。切缘阴性意味着在显微镜下检查切除组织的边缘时，没有发现癌细胞的存在。这通常是通过病理学家对切除组织进行详细的组织学检查来确定的。 切缘宽度：虽然没有明确的最佳切缘宽度标准，但通常建议至少达到2毫米的无癌组织宽度。更宽的切缘可能进一步降低局部复发的风险，但这需要在保证乳房外观和功能的前提下进行权衡。 再次切除：如果初次手术后的病理检查显示切缘阳性（即有癌细胞残留），则可能需要进行再次手术以扩大切除范围，直至达到切缘阴性的状态。这可以通过再次乳房肿瘤切除术或全乳房切除术来实现。 术中评估：在一些先进的医疗机构，可能会使用术中快速冰冻病理检查来评估切缘状态。这种方法可以在手术过程中快速确定切缘是否有癌细胞残留，从而指导手术医生是否需要进一步扩大切除范围。 请注意，以上信息仅供参考，并不能替代专业医疗建议。具体的治疗方案和手术要求应由经验丰富的乳腺癌外科医生根据患者的具体情况来制定。 | |
| 4. For cN1 breast cancer patients who have converted to cN0 after neoadjuvant therapy, what are the requirements for sentinel lymph node biopsy at this stage? | Hey there! When dealing with cN1 (clinically node-positive) breast cancer patients who convert to cN0 (clinically node-negative) after neoadjuvant therapy, the decision on sentinel lymph node biopsy (SLNB) depends on a few factors:  1. **Initial Node Status (cN1 to cN0 conversion):** If neoadjuvant therapy successfully downstages the lymph nodes from cN1 to cN0, it suggests a good response. In such cases, SLNB might be considered.  2. **Pathologic Complete Response (pCR):** If the patient achieves a pathologic complete response (no residual tumor in the breast or lymph nodes) after neoadjuvant therapy, SLNB may not be necessary.  3. **Extent of Axillary Disease:** The extent of initial axillary involvement and the response to neoadjuvant therapy play a role. If there was extensive nodal involvement initially, clinicians might opt for SLNB even with conversion to cN0.  4. **Guidelines and Individualized Treatment:** Adherence to established guidelines and individualized treatment decisions are crucial. Guidelines from organizations like the American Society of Clinical Oncology (ASCO) and the National Comprehensive Cancer Network (NCCN) can provide specific recommendations.  5. **Multidisciplinary Team Discussion:** Decision-making often involves a multidisciplinary team, including surgeons, oncologists, and pathologists. Their collective expertise ensures comprehensive evaluation and decision-making.  6. **Consideration of Patient Preferences:** Patient preferences and values are essential in the decision-making process. Some patients may prefer a less invasive approach if the risk of axillary involvement is deemed low.  In summary, the decision on SLNB after neoadjuvant therapy for cN1 to cN0 conversion involves a nuanced assessment of various factors. It's recommended to consult with a breast cancer specialist or a multidisciplinary team to determine the most appropriate course of action based on the individual patient's characteristics and treatment response. | Hey there! When dealing with cN1 (clinically node-positive) breast cancer patients who've converted to cN0 (clinically node-negative) after neoadjuvant therapy, the decision for sentinel lymph node biopsy (SLNB) depends on a few factors:  1. **Response to Neoadjuvant Therapy:** If the patient shows a complete response to neoadjuvant therapy, meaning there's no evidence of residual disease in the lymph nodes after treatment, SLNB might not be necessary.  2. **Initial Extent of Disease:** The extent of lymph node involvement before neoadjuvant therapy is crucial. If the initial staging indicated extensive lymph node involvement, even with a complete response, the decision for SLNB might lean towards caution.  3. **Triple-Negative Breast Cancer (TNBC):** For patients with triple-negative breast cancer, SLNB is often recommended regardless of the response to neoadjuvant therapy, as TNBC can sometimes have a higher chance of residual disease.  4. **Tumor Subtype and Grade:** The subtype and grade of the tumor can influence the decision. Low-grade tumors with a good response to therapy may have a lower likelihood of nodal involvement.  5. **Clinical Judgment:** Ultimately, the decision for SLNB involves clinical judgment. Consultation with a multidisciplinary team, including surgeons, oncologists, and pathologists, is essential to tailor the approach based on the individual patient's circumstances.  It's important to note that guidelines may evolve, so always consider the latest recommendations from reputable oncology organizations. If you have specific patient details, consulting with a healthcare professional would provide the most personalized advice. | cN1患者经新辅助治疗后变为cN0，这种情况下对前哨淋巴结活检有一些特殊的要求和考虑因素。在这个背景下，以下是需要考虑的关键方面：  1. **确认临床响应：** 在进行前哨淋巴结活检之前，需要确认患者对新辅助治疗的临床响应。这包括通过临床检查、影像学和其他相应的评估手段，确保肿瘤在新辅助治疗后得到了有效的缩小或消失。  2. **影像学评估：** 使用影像学技术，如超声、MRI等，对淋巴结进行全面的评估。这有助于确定患者是否有其他淋巴结受累的证据，以及确认新辅助治疗对淋巴结的影响。  3. **综合考虑其他指标：** 除了临床响应和影像学评估外，还应该考虑其他指标，如肿瘤的分子亚型、组织学类型等。这些信息可以帮助确定是否需要进行前哨淋巴结活检以及如何选择适当的活检方法。  4. **手术计划的调整：** 如果患者经新辅助治疗后从cN1变为cN0，可能需要调整手术计划。这可能包括重新评估手术的范围和淋巴结清扫的需求。  5. **术前标记技术：** 考虑使用术前标记技术，如放射性同位素标记或纳米标记物，以提高前哨淋巴结活检的准确性。这有助于在手术中更精确地定位淋巴结。  需要注意的是，这些建议是一般性的，具体的操作和决策应由乳腺癌专业团队根据患者的具体情况和最新的临床指南来制定。 | similar | 作为乳腺癌专家，对于乳腺癌的诊断和治疗都有深入的了解。针对你所描述的问题，即cN1（临床腋窝淋巴结阳性）患者经新辅助治疗后变为cN0（临床腋窝淋巴结阴性）时，对前哨淋巴结活检的要求，以下是详细的解答：  前哨淋巴结活检（SLNB）是一种用于评估乳腺癌患者腋窝淋巴结状态的手术技术。在cN1患者经新辅助治疗后转为cN0的情况下，进行SLNB的主要目的是确认腋窝淋巴结的病理状态，以指导后续的治疗策略。  对于这类患者，进行SLNB时应满足以下要求：  技术熟练：执行SLNB的医生应具备丰富的经验和熟练的技术，以确保活检的准确性和安全性。 使用合适的示踪剂：为了准确识别前哨淋巴结，应使用合适的示踪剂，如蓝色染料和/或放射性胶体。这些示踪剂有助于在手术中精确定位前哨淋巴结。 确保取样的代表性：应至少取出2个（最好3个）蓝色的淋巴结和/或任何放射性阳性的淋巴结进行病理检查。这样可以提高检测的准确性，减少假阴性的风险。 详细的病理检查：对取出的淋巴结应进行详细的病理检查，包括常规苏木精-伊红染色和免疫组织化学染色等，以准确评估淋巴结的状态。 考虑新辅助治疗的影响：由于患者接受了新辅助治疗，应意识到这可能对淋巴结的病理表现产生影响。因此，在解读病理结果时应特别谨慎。 制定后续治疗策略：根据SLNB的结果，结合患者的其他临床和病理特征，制定合适的后续治疗策略。例如，如果SLNB结果为阴性，可能考虑避免进一步的腋窝淋巴结清扫手术。 请注意，以上内容仅供参考，并不能替代专业医疗建议。在实际操作中，应根据患者的具体情况和医生的判断来制定合适的治疗方案。 | similar | |
| 5. What surgical methods do you know for stage I breast reconstruction? | Certainly! In the context of stage I breast reconstruction, several surgical methods are commonly considered. It's essential to note that the choice of method depends on various factors, including the patient's preferences, medical history, and the specific characteristics of the breast cancer and treatment. Here are some surgical methods for stage I breast reconstruction:  1. **Breast Implants (Implant-Based Reconstruction):**  - *Procedure:* In this method, a silicone or saline implant is used to reconstruct the breast. The surgeon may place the implant either directly under the chest muscle or over the muscle, depending on the patient's anatomy and other factors.  - *Advantages:* Generally a quicker procedure with a shorter recovery time compared to other methods.  2. **Autologous Tissue Flap Reconstruction:**  - *Procedure:* This method involves using the patient's own tissue, usually from the abdomen (TRAM flap), buttocks (SGAP flap), or thighs (TMG flap), to reconstruct the breast. The tissue is transferred along with its blood supply to create a new breast mound.  - *Advantages:* Results in a more natural look and feel. Can provide additional benefits like tummy tuck (in the case of TRAM flap).  3. **Latissimus Dorsi Flap Reconstruction:**  - *Procedure:* This technique uses muscle, fat, and skin from the back (latissimus dorsi muscle) to reconstruct the breast. It's often combined with an implant to achieve the desired volume.  - *Advantages:* Suitable for patients who may not be candidates for other types of autologous flap reconstruction.  4. **Nipple-Sparing Mastectomy with Reconstruction:**  - *Procedure:* In certain cases, the nipple can be preserved during mastectomy, and reconstruction is performed either immediately or in a staged manner.  - *Advantages:* Preserving the nipple can contribute to a more natural-looking reconstructed breast.  5. **Direct-to-Implant (One-Stage) Reconstruction:**  - *Procedure:* This method involves placing a breast implant immediately after the mastectomy in a single surgery.  - *Advantages:* Reduces the need for tissue expanders and multiple surgeries, providing a quicker reconstruction process.  It's crucial for patients to have detailed discussions with their healthcare team, including a plastic surgeon, to determine the most suitable reconstruction method based on individual circumstances and preferences. The decision-making process should consider factors such as the patient's overall health, body type, and the extent of breast tissue removal. | similar | I期乳房重建通常是指乳腺癌手术后进行的乳房再建手术。有几种常见的手术方式，具体选择会根据患者的健康状况、个体差异以及医生的建议而定。以下是一些可能的手术方式：  1. **乳房植入物重建（Breast Implant Reconstruction）：** 这是一种常见的方法，其中植入物被置于乳腺区域，以还原乳房的形状和容积。植入物可以是盐水或硅胶填充的。  2. **自体组织移植（Autologous Tissue Reconstruction）：** 这种方法使用患者自己的组织，通常是腹部、臀部或大腿的皮肤和脂肪，来重建乳房。这包括LD 肌肉皮瓣、腹直肌皮瓣等。  3. **乳头-乳晕复合体重建（Nipple-Areola Complex Reconstruction）：** 在乳房重建的过程中，还可以考虑对乳头和乳晕进行再建，以增强外观的自然性。  4. **两侧对称整形（Contralateral Symmetry Procedures）：** 为了达到更加对称的外观，有时候需要对对侧的乳房进行整形手术，以匹配重建的乳房。  5. **联合手术（Combined Procedures）：** 在一些情况下，医生可能会采用多种手术方式的组合，以达到最佳的整体效果。  这只是一些常见的选择，具体的手术方式会因个体差异而异。患者应与医疗团队充分讨论各种选项，包括手术的风险和优势，以便做出符合个人情况的明智决定。 | similar | 作为乳腺癌专家，对于乳房重建的手术方式有着深入了解。乳房重建手术旨在帮助乳腺癌患者在切除肿瘤后恢复乳房的外观和形态，从而提高其生活质量和心理健康。以下是关于I期乳房重建的手术方式：  植入物重建：这是一种常见的乳房重建方式。在此过程中，外科医生会在胸部的肌肉下放置一个乳房植入物（硅胶或盐水填充），以模拟自然乳房的形状和大小。植入物可以根据患者的需求和身体情况进行定制。 自体组织重建（皮瓣重建）：这种方式涉及使用患者自身的组织来重建乳房。外科医生会从患者的腹部、背部或臀部等部位切取一部分组织（包括皮肤、脂肪和有时还包括肌肉），然后将其移植到胸部以形成新的乳房。这种方法可以产生非常自然的外观和感觉，因为使用的是患者自己的组织。根据切取组织的方式和位置，自体组织重建有多种技术，如TRAM皮瓣、DIEP皮瓣和LATF皮瓣等。 组合重建：在某些情况下，外科医生可能会选择结合使用植入物和自体组织进行乳房重建。例如，他们可能会先使用植入物来创建乳房的基础结构，然后再在顶部添加一层自体组织，以获得更自然的外观和感觉。 这些手术方式的选择取决于多种因素，包括患者的健康状况、手术史、个人偏好以及外科医生的经验和专长。每种方法都有其独特的优点和潜在的并发症，因此，在决定接受哪种乳房重建手术之前，患者应与外科医生进行详细的咨询和讨论。  请注意，虽然乳房重建手术可以帮助患者恢复自信和改善生活质量，但它并不能完全替代自然乳房。因此，患者在做出决定之前应充分了解手术的风险、恢复过程和预期效果。同时，术后的护理和康复也非常重要，患者需遵循医生的术后指导，包括伤口护理、锻炼和定期随访等，以促进愈合和减少并发症的风险。 | similar | |
| 6. What is the strategy for adjuvant chemotherapy in early-stage triple-negative breast cancer? | Certainly! Adjuvant chemotherapy for early-stage triple-negative breast cancer (TNBC) is a crucial component of the treatment strategy. TNBC is characterized by the absence of estrogen receptor (ER), progesterone receptor (PR), and human epidermal growth factor receptor 2 (HER2) expression. Given its aggressive nature, a multimodal approach is often employed. Here's a detailed explanation:  **1. **Surgery**: The primary treatment for early-stage breast cancer is surgery, usually either a lumpectomy (removal of the tumor and surrounding tissue) or a mastectomy (removal of the entire breast). The choice depends on various factors, including tumor size and patient preference.  **2. **Adjuvant Chemotherapy**: Since TNBC lacks specific receptors targeted by hormonal therapies (ER, PR) or HER2-targeted therapies, chemotherapy plays a central role. Anthracycline- and taxane-based regimens are commonly used. Examples include the combination of doxorubicin and cyclophosphamide followed by paclitaxel or docetaxel.  **3. **Timing and Duration**: Adjuvant chemotherapy is administered after surgery to eliminate any remaining cancer cells. The duration and frequency of chemotherapy cycles depend on the specific regimen chosen. Typically, treatment lasts for several months.  **4. **Response Monitoring**: During and after chemotherapy, patients are monitored for response to treatment through imaging studies and other tests. This helps assess the effectiveness of the therapy and guides further management.  **5. **Consideration of Clinical Trials**: Given the aggressive nature of TNBC, participation in clinical trials is often considered. New and experimental treatments may provide additional options for patients with TNBC.  **6. **Radiation Therapy**: Depending on the extent of surgery and other factors, radiation therapy may be recommended to target any remaining cancer cells and reduce the risk of recurrence.  **7. **Follow-up Care**: After completing adjuvant chemotherapy, patients undergo regular follow-up care, including imaging studies and blood tests, to monitor for any signs of recurrence and manage potential side effects of treatment.  It's important to note that the specific treatment plan may vary based on individual patient characteristics and advancements in medical research. Consultation with an oncologist is crucial for tailoring the treatment to the specific needs of the patient. Always seek advice from healthcare professionals for the most accurate and up-to-date information. | similar | 早期三阴性乳腺癌是一种缺乏雌激素受体（ER）、孕激素受体（PR）和人类表皮生长因子受体2（HER2）表达的乳腺癌类型。辅助化疗在早期乳腺癌治疗中扮演着重要的角色，特别是对于三阴性乳腺癌。  辅助化疗的策略通常取决于多个因素，包括患者的整体健康状况、肿瘤的大小、淋巴结的状态以及分子亚型等。以下是一般情况下对早期三阴性乳腺癌辅助化疗的一些建议：  1. **多药联合疗法（Combination Chemotherapy）：** 通常使用多种药物的组合，这有助于提高治疗的有效性。常用的药物包括顺铂（cisplatin）、多西他赛（docetaxel）、卡巴班酯（carboplatin）等。  2. **术前化疗（Neoadjuvant Chemotherapy）：** 在手术前进行化疗，有助于缩小肿瘤的大小，使手术更容易实施。这也可以评估肿瘤对化疗的敏感性。  3. **辅助化疗的持续时间：** 通常，化疗的疗程为数个周期，每个周期之间有一定的间隔。具体的疗程可能因患者的具体情况而异。  4. **个体化治疗：** 针对肿瘤的分子特征，可以考虑使用靶向治疗。然而，在三阴性乳腺癌的情况下，由于缺乏ER、PR和HER2的表达，靶向治疗的选择相对有限。  5. **治疗反应监测：** 在化疗过程中，医生通常会监测肿瘤的反应。这有助于调整治疗方案，确保最佳的疗效。  需要注意的是，以上只是一般性的建议，具体的治疗策略应该由专业的乳腺癌团队根据患者的具体情况进行制定。早期三阴性乳腺癌的治疗是一个复杂而个体化的过程，需要综合考虑多个因素。 | 在处理早期三阴性乳腺癌时，辅助化疗是常见的治疗策略之一。请注意，我的知识截止日期是2021年9月，因此最新的指南和研究可能有所更新。以下是一般性的策略，具体的治疗方案应该由专业医生根据患者的具体情况制定。  1. **手术治疗：** 通常，早期乳腺癌的首要治疗是手术切除肿瘤，可能是保乳手术或全乳切除。  2. **辅助化疗：** 对于三阴性乳腺癌，辅助化疗在手术前或手术后被用来降低复发风险。这可能包括使用化疗药物，如紫杉醇（paclitaxel）、多西他赛（doxorubicin）和环磷酰胺（cyclophosphamide）等。  3. **个体化治疗：** 乳腺癌的分子亚型对治疗的选择至关重要。对于三阴性乳腺癌，特别是在HER2（人类表皮生长因子受体2）阴性的情况下，激素治疗可能不起作用。因此，个体化的治疗方案会考虑到这些因素。  4. **放疗：** 辅助放疗可能会在手术后进行，以减少局部复发的风险。  5. **临床试验：** 有时医生会鼓励患者参与临床试验，以尝试新的治疗方法或药物。  总体来说，治疗策略的选择应该根据患者的具体病情、病理学特征和分子亚型来制定。患者应该与专业的乳腺癌医生进行深入的讨论，以确保制定最合适的治疗计划。 | 作为乳腺癌专家，对于乳腺癌的各类型及各阶段的诊疗方案都有深入的了解。以下针对早期三阴性乳腺癌（TNBC）的辅助化疗策略进行解答：  三阴性乳腺癌是指雌激素受体（ER）、孕激素受体（PR）和人表皮生长因子受体2（HER2）均为阴性的乳腺癌。这种类型的乳腺癌通常对激素治疗和HER2靶向治疗不敏感，因此化疗在TNBC的治疗中扮演着重要角色。  对于早期TNBC，辅助化疗的主要目的是减小肿瘤大小、消灭微小转移灶，从而降低复发风险，并提高患者的生存率。具体的化疗策略通常根据患者的个体情况（如肿瘤大小、淋巴结状态、病理分级等）和医生的经验来选择。  常用的化疗药物包括蒽环类药物（如多柔比星、表柔比星等）和紫杉类药物（如紫杉醇、多西他赛等）。这些药物可以单独使用，也可以联合使用。例如，AC方案（多柔比星+环磷酰胺）和TAC方案（多西他赛+多柔比星+环磷酰胺）是常用的联合化疗方案。  近年来，一些新的化疗药物和方案也在不断研究中，如铂类药物（如顺铂、卡铂等）在TNBC中的应用也取得了一定的疗效。此外，还有一些研究在探索将免疫治疗与化疗相结合的策略，以期进一步提高治疗效果。  需要注意的是，化疗药物的使用需要严格遵循医生的建议和指导，因为化疗药物在杀灭癌细胞的同时，也会对正常细胞造成一定的损伤，从而产生一些副作用。因此，在化疗期间，患者需要密切关注自己的身体状况，并及时向医生反馈任何不适。  最后，需要强调的是，早期TNBC的辅助化疗策略并非一成不变，而是需要根据患者的具体情况和最新的研究进展进行个体化的调整和优化。因此，建议患者与医生保持密切的沟通和合作，共同制定最适合自己的治疗方案。 | 作为乳腺癌专家，深知三阴性乳腺癌相比其他类型的乳腺癌更具有侵袭性，且由于其激素受体阴性和HER2阴性的特点，很多靶向治疗或激素治疗不适用。因此，化疗在治疗三阴性乳腺癌中扮演着非常重要的角色。  对于早期三阴性乳腺癌，辅助化疗是标准治疗方案之一，主要策略包括：  蒽环类与紫杉类化疗药物组合：这是最常用的化疗方案之一。蒽环类药物（如阿霉素、表阿霉素）和紫杉类药物（如紫杉醇、多西紫杉醇）在多个临床试验中被证明对三阴性乳腺癌有效。  密集化疗：通过缩短化疗周期之间的间隔，增加化疗药物的剂量密度，以期在更短的时间内给予更多的治疗，从而可能提高治疗效果。但这也可能增加副作用。  序贯化疗：即先使用一种或一组化疗药物，然后再使用另一种或另一组药物。这种策略可以针对不同阶段的肿瘤细胞，或利用不同药物之间的协同作用。  辅助放疗：对于部分高危患者，如肿瘤较大、淋巴结阳性等，化疗后可能还需要辅助放疗以降低局部复发的风险。  临床试验与新药探索：由于三阴性乳腺癌的治疗挑战，许多患者可能会考虑参加临床试验，以尝试新的治疗策略或药物。  个体化治疗：根据患者的具体情况（如年龄、身体状况、肿瘤特征等）和基因检测结果，制定个体化的化疗方案。  持续监测与随访：化疗结束后，患者需要接受定期的监测和随访，以确保肿瘤没有复发或转移，并及时处理任何可能的长期副作用。  值得注意的是，化疗药物的选择和方案的制定应基于患者的具体情况和医生的建议。化疗可能带来一系列的副作用，如恶心、呕吐、脱发、骨髓抑制等，因此在治疗过程中需要密切监测患者的身体状况，并及时调整治疗方案以减轻副作用。  以上信息仅供参考，如有需要，建议咨询专业的乳腺癌医生或医疗团队。 | |
| 7. For early-stage high-risk breast cancer patients with strongly positive hormone receptors, which adjuvant endocrine therapy would you recommend? | Hey there! When it comes to early-stage high-risk breast cancer patients with strongly positive hormone receptors, the choice of adjuvant endocrine therapy depends on several factors. The two main classes of drugs used for this purpose are aromatase inhibitors (AIs) and selective estrogen receptor modulators (SERMs). Let's break it down:  1. **Aromatase Inhibitors (AIs):**  - Examples: Anastrozole, Letrozole, Exemestane.  - AIs are often considered the preferred choice, especially in postmenopausal women, as they inhibit the production of estrogen in peripheral tissues.  2. **Selective Estrogen Receptor Modulators (SERMs):**  - Example: Tamoxifen.  - Tamoxifen is an option for premenopausal and postmenopausal women. It works by blocking estrogen receptors.  **Recommendation:**  - For postmenopausal women, AIs are generally the preferred choice due to their effectiveness in reducing estrogen levels.  - For premenopausal women, Tamoxifen may be the initial choice, and in some cases, it might be followed by an AI.  The duration of endocrine therapy is typically recommended for 5 to 10 years, and the decision may be influenced by the specific characteristics of the tumor and the patient's overall health.  It's crucial to note that individual cases may vary, and the final decision should be made after a thorough discussion between the patient and their oncologist, considering factors like age, potential side effects, and overall health.  Feel free to ask if you have more questions or need further clarification! | similar | 对于激素受体强阳性的早期高危乳腺癌患者，一般来说，辅助内分泌治疗是一种常见的治疗方案。最常用的药物包括：  1. **泛素酮（Tamoxifen）：** 这是一种选择性雌激素受体调节剂，用于预防和治疗激素受体阳性的乳腺癌。它通常用于更年期前的妇女。  2. **阿那曲唑（Anastrozole）、莱特罗唑（Letrozole）、依西美坦（Exemestane）等 aromatase 抑制剂：** 这些药物通过抑制体内雌激素的产生，从而减少对乳腺癌细胞的刺激。它们通常在更年期后的妇女中使用。  在具体推荐哪种药物之前，需要考虑患者的年龄、更年期状态、合并症等因素。这些决定通常由乳腺癌专业医生根据患者的具体情况来制定。因此，建议您咨询专业医生，以确保制定的治疗方案最适合患者的需要。 | similar | 作为乳腺癌专家，深知乳腺癌的治疗需要综合考虑患者的具体情况，包括肿瘤的分期、分级、激素受体状态等因素。对于激素受体强阳性的早期高危乳腺癌患者，辅助内分泌治疗是非常重要的一部分。  内分泌治疗主要是通过调节患者体内的激素水平，来抑制肿瘤的生长和扩散。对于激素受体强阳性的乳腺癌患者，内分泌治疗的效果通常比较好。  目前，常用的辅助内分泌治疗药物包括选择性雌激素受体调节剂（如他莫昔芬、托瑞米芬等）和芳香化酶抑制剂（如阿那曲唑、来曲唑、依西美坦等）。这些药物的作用机制不同，但都能有效地降低患者体内的雌激素水平，从而抑制肿瘤的生长。  在选择具体的内分泌治疗方案时，需要考虑患者的年龄、月经状况、肿瘤分期、分级以及患者是否存在其他健康问题等因素。例如，对于绝经前的女性患者，通常会选择选择性雌激素受体调节剂进行治疗；而对于绝经后的女性患者，则更倾向于使用芳香化酶抑制剂。  此外，新型内分泌治疗药物，如CDK4/6抑制剂等也在临床研究中显示出了一定的疗效，对于某些高危患者，也可以考虑在标准内分泌治疗的基础上联合使用这些药物。  总的来说，对于激素受体强阳性的早期高危乳腺癌患者，我会根据患者的具体情况推荐合适的辅助内分泌治疗方案。同时，我也会密切监测患者的治疗反应和耐受性，并根据需要及时调整治疗方案，以确保患者能够获得最佳的治疗效果和生活质量。 | similar | |
| 8. What are the different classes of drugs for anti-HER2 therapy? | Certainly! In the realm of anti-HER2 therapy, there are several classes of drugs that are commonly used. These drugs are designed to target the human epidermal growth factor receptor 2 (HER2), which is overexpressed in some breast cancers. Here are the main classes of drugs used in anti-HER2 therapy:  1. **Monoclonal Antibodies (mAbs):**  - *Trastuzumab (Herceptin):* This was one of the pioneering drugs in anti-HER2 therapy. It is a monoclonal antibody that targets the HER2 protein on the surface of cancer cells, inhibiting their growth.   - *Pertuzumab (Perjeta):* Another monoclonal antibody, pertuzumab works in conjunction with trastuzumab, targeting a different region of the HER2 receptor. This combination is often used in the treatment of HER2-positive breast cancer.  2. **Tyrosine Kinase Inhibitors (TKIs):**  - *Lapatinib (Tykerb):* Lapatinib is a tyrosine kinase inhibitor that blocks signals inside the cancer cell that promote its growth. It is often used in combination with other anti-HER2 therapies.   - *Neratinib (Nerlynx):* This is another tyrosine kinase inhibitor that works by inhibiting multiple receptors, including HER2. It is used as an adjuvant therapy in certain cases.  3. **Antibody-Drug Conjugates (ADCs):**  - *Ado-Trastuzumab Emtansine (Kadcyla):* This is a unique form of therapy where a chemotherapy drug is linked to the trastuzumab antibody. It delivers the chemotherapy directly to the cancer cells, minimizing damage to normal cells.  4. **Bispecific Antibodies:**  - *Trastuzumab Deruxtecan (Enhertu):* This is a newer class of drug that combines trastuzumab with a chemotherapy drug. It has shown effectiveness in patients who have previously received other anti-HER2 therapies.  These drugs may be used alone or in combination depending on the specific characteristics of the cancer and the treatment plan devised by the oncologist. The goal is to effectively target and inhibit the HER2 protein, thereby impeding the growth of HER2-positive breast cancer cells. | similar | 抱歉，我并不是一位医生，也不是乳腺癌专家。然而，我可以为你提供一些一般性的信息。抗HER2治疗通常可以分为几类：  1. **单克隆抗体：** 这是一类通过基因工程技术制造的抗体，专门针对HER2受体。例子包括特鲁普鲁茨单抗（Trastuzumab）和帕博利珠单抗（Pertuzumab）。  2. **酪氨酸激酶抑制剂：** 这类药物通过抑制HER2受体上的激酶活性来阻止信号传导。拉普尼希尼布（Lapatinib）就是这一类的代表。  3. **ADC药物：** 这是一种将化疗药物与抗体结合的治疗方法，通过抗体将化疗药物精确地送到HER2受体处。特拉斯图固定（Trastuzumab emtansine）是其中的一种。  请注意，具体的治疗方案和药物选择可能会因患者的具体情况而异，只有专业医生才能为患者提供个性化的建议。如果你或者你认识的人面临乳腺癌问题，强烈建议咨询专业医生以获取准确的信息和建议。 | similar | 作为乳腺癌专家，我深知HER2（人类表皮生长因子受体2）阳性乳腺癌是一种特殊类型的乳腺癌，其癌细胞表面有过多的HER2蛋白。这种过量的蛋白会促进癌细胞的生长和分裂。因此，针对HER2的靶向治疗在乳腺癌治疗中占有重要地位。  目前，抗HER2治疗的药物主要可以分为以下几类：  单克隆抗体药物：这类药物能够特异性地结合到HER2蛋白上，从而阻断其信号传导，抑制癌细胞的生长。例如，曲妥珠单抗（Trastuzumab）是第一个被批准用于临床的抗HER2单克隆抗体，它能够与HER2蛋白结合，激活机体的免疫系统来攻击癌细胞。帕妥珠单抗（Pertuzumab）是另一种单克隆抗体，它与HER2蛋白的不同部位结合，进一步增强了对HER2阳性乳腺癌的治疗效果。 抗体药物偶联物（ADCs）：这类药物将单克隆抗体与细胞毒性药物连接在一起，使药物能够更精确地输送到癌细胞中，减少对正常细胞的损害。例如，曲妥珠单抗-美坦新偶联物（Trastuzumab emtansine）就是这样一种药物，它将曲妥珠单抗与一种细胞毒性药物连接在一起，提高了对HER2阳性乳腺癌的治疗效果。 小分子酪氨酸激酶抑制剂（TKIs）：这类药物能够抑制HER2蛋白的酪氨酸激酶活性，从而阻断其信号传导。例如，拉帕替尼（Lapatinib）和奈拉替尼（Neratinib）就是这样的小分子抑制剂，它们能够穿透细胞膜，直接抑制HER2蛋白的活性。 这些药物可以单独使用，也可以与其他化疗药物或激素治疗药物联合使用，以提高对HER2阳性乳腺癌的治疗效果。然而，需要注意的是，不同患者对这些药物的反应可能会有所不同，因此在选择治疗方案时应根据患者的具体情况进行个体化治疗。  请注意，以上信息仅供参考，具体的治疗方案需要由医生根据患者的具体情况来制定。 | similar | |
| 9. What is the first-line treatment of choice for stage IV or recurrent metastatic HR-positive/HER2-negative breast cancer? | Certainly, I'd be happy to provide information on the first-line treatment for stage IV or recurrent metastatic HR-positive/HER2-negative breast cancer.  The primary goal in treating metastatic HR-positive/HER2-negative breast cancer is typically to control the disease, alleviate symptoms, and improve the patient's quality of life. The first-line treatment often involves endocrine therapy, which targets the hormone receptors (estrogen or progesterone receptors) on the cancer cells.  **First-Line Treatment Options:**  1. **Aromatase Inhibitors (AI):** These drugs, such as anastrozole, letrozole, and exemestane, block the production of estrogen in postmenopausal women. They are often used as the first-line treatment for HR-positive breast cancer.  2. **Selective Estrogen Receptor Modulators (SERMs):** Tamoxifen is a commonly used SERM. It interferes with the action of estrogen in the breast tissue. Tamoxifen is sometimes used in premenopausal women or postmenopausal women who cannot tolerate AIs.  3. **CDK4/6 Inhibitors:** Drugs like palbociclib, ribociclib, and abemaciclib are often used in combination with endocrine therapy. They target specific proteins involved in cell cycle regulation and enhance the effectiveness of hormonal therapy.  4. **Fulvestrant:** This is another option used in postmenopausal women. It acts as both an estrogen receptor antagonist and downregulator.  **Considerations:**  - **Patient Characteristics:** The choice of therapy may depend on factors such as menopausal status, overall health, and previous treatments.  - **Combination Therapies:** Sometimes, a combination of endocrine therapy and targeted therapies may be recommended for more aggressive disease.  - **Regular Monitoring:** Periodic monitoring through imaging studies and other tests is crucial to assess the response to treatment and make adjustments if needed.  It's important to note that treatment decisions are highly individualized, and the oncologist will consider various factors before determining the most suitable approach for a specific patient. Always consult with a healthcare professional for personalized advice based on the latest medical information. | similar | IV期或复发性转移性HR阳性/HER2阴性乳腺癌的首选一线治疗通常包括内分泌治疗。对于这种类型的乳腺癌，激素受体阳性（HR阳性）意味着肿瘤对雌激素或孕激素敏感，因此抑制这些激素的作用是一种有效的治疗策略。  常见的内分泌治疗包括使用抑制雌激素生产的药物，如芳香化酶抑制剂（比如阿那曲唑）或阻断雌激素受体的药物，比如抗雌激素药物（比如他莫昔芬）。这些治疗可以帮助控制肿瘤的生长，并在一定程度上延长患者的生存期。  需要注意的是，具体的治疗方案可能因患者的具体情况而有所不同，包括年龄、整体健康状况、以及其他潜在的合并症。因此，最佳的治疗决策应该由患者与其医疗团队共同商讨。在制定治疗计划时，通常还会考虑其他因素，比如手术、放疗以及可能的靶向治疗。  请注意，这些信息可能会随着时间的推移而发生变化，因此建议在采取任何治疗措施之前，与医疗专业人士进行详细咨询。 | similar | 作为乳腺癌专家，深知乳腺癌是一种复杂的疾病，其治疗需要根据患者的具体情况进行个体化定制。针对IV期或复发性转移性HR阳性（激素受体阳性）/HER2阴性乳腺癌，首选的一线治疗方案通常基于内分泌治疗，因为这类乳腺癌的生长往往依赖于雌激素。  内分泌治疗：这是HR阳性乳腺癌的主要治疗手段。通过药物来降低体内雌激素水平或阻止雌激素与乳腺癌细胞上的受体结合，从而抑制肿瘤的生长。常用的内分泌治疗药物包括：  选择性雌激素受体调节剂（SERMs），如他莫昔芬。 芳香化酶抑制剂（AIs），如来曲唑、阿那曲唑和依西美坦，这些药物通过阻止雌激素的生成来发挥作用。 对于绝经前女性，还可能使用卵巢功能抑制（如通过手术、放疗或药物抑制卵巢功能）。 靶向治疗：虽然问题是针对HER2阴性的乳腺癌，但对于部分有特定基因改变的患者，也可能考虑使用靶向治疗药物。不过，这并不是首选一线治疗，而是作为内分泌治疗的辅助或后续治疗。  化疗：在某些情况下，如果内分泌治疗不足以控制疾病进展，或者患者病情较为严重，医生可能会考虑使用化疗药物。化疗可以杀死快速分裂的癌细胞，包括乳腺癌细胞。  局部治疗：对于某些局部复发或转移的患者，可能还需要手术或放疗来控制局部病灶。  临床试验：对于一些标准治疗不再有效的患者，参与临床试验可能是一个选择。鼓励患者可以尝试新的治疗方法，同时也为医学研究和未来患者的治疗做出贡献。  总之，针对IV期或复发性转移性HR阳性/HER2阴性乳腺癌，首选的一线治疗通常是内分泌治疗。但具体的治疗方案需要根据患者的年龄、健康状况、肿瘤特性以及先前的治疗反应等因素来综合决定。因此，强烈建议患者在专业医生的指导下进行治疗。 | 作为乳腺癌专家，对于不同分型和分期的乳腺癌治疗有着深入的了解。针对IV期或复发性转移性HR阳性（激素受体阳性）/HER2阴性乳腺癌，其首选的一线治疗方案主要是基于激素治疗，特别是使用内分泌治疗药物。  这类乳腺癌患者的肿瘤表达激素受体，尤其是雌激素受体（ER）和/或孕激素受体（PR），这意味着肿瘤的生长和发展可能受到体内激素的影响。因此，通过药物来降低或阻断这些激素的作用，可以达到控制肿瘤生长的目的。  一线治疗中常用的药物包括选择性雌激素受体调节剂（SERMs，如他莫昔芬）、芳香化酶抑制剂（AIs，如阿那曲唑、来曲唑和依西美坦）等。这些药物通过不同的机制减少体内雌激素的水平或阻断雌激素与其受体的结合，从而抑制肿瘤的生长。  需要注意的是，虽然内分泌治疗是这类乳腺癌的首选一线治疗，但具体的治疗方案应根据患者的具体情况（如年龄、月经状态、肿瘤特点、先前治疗等）进行个体化选择。此外，随着医学研究的不断进步，新的治疗方法和药物也在不断涌现，因此，患者在接受治疗时应与医生充分沟通，了解最新的治疗动态和可能的治疗选择。  总的来说，IV期或复发性转移性HR阳性/HER2阴性乳腺癌的首选一线治疗是基于激素的内分泌治疗，具体药物和方案应根据患者的个体情况来确定。 | |
| 10. What are the conditions for exemption from radiotherapy after breast-conserving surgery | Certainly! It's important to note that decisions about exemption from radiotherapy after breast-conserving surgery are complex and depend on various factors. I'll provide a general overview, but it's crucial to consult with a qualified healthcare professional for personalized advice.  **Conditions for Exemption from Radiotherapy after Breast-Conserving Surgery:**  1. **Low Risk of Recurrence:**  - Patients with a low risk of recurrence based on the characteristics of the tumor may be considered for exemption.  - Factors such as tumor size, grade, hormone receptor status, and lymph node involvement are assessed.  2. **Clear Surgical Margins:**  - Adequate surgical margins, meaning that no cancer cells are found at the edges of the removed tissue, increase the likelihood of exemption.  3. **Age and Health Status:**  - Older age or certain health conditions may influence the decision. In some cases, the potential risks of radiotherapy may outweigh the benefits, especially if life expectancy is limited.  4. **Patient Preferences:**  - Patient preferences and values play a role. Some individuals may choose to forgo radiotherapy due to concerns about potential side effects.  5. **Receptor Status:**  - The hormone receptor status of the tumor (estrogen and progesterone receptors) and human epidermal growth factor receptor 2 (HER2) status are considered in the decision-making process.  6. **Genetic Factors:**  - Genetic testing for mutations such as BRCA1 and BRCA2 may influence the decision, especially if the cancer is not associated with high genetic risk.  7. **Postoperative Pathological Assessment:**  - Detailed examination of the tumor tissue after surgery helps in determining the aggressiveness of the cancer and guides treatment decisions.  8. **Adjuvant Therapies:**  - If other adjuvant therapies such as hormonal therapy or chemotherapy are recommended, they may influence the decision regarding radiotherapy.  9. **Multidisciplinary Consultation:**  - A multidisciplinary team, including surgeons, oncologists, and radiation oncologists, collaborates to make the best-informed decision based on the individual patient's case.  It's essential to emphasize that each case is unique, and decisions should be made in consultation with a healthcare team. The goal is to balance the benefits of reducing the risk of cancer recurrence with potential side effects and individual preferences. | similar | 保乳术后是否需要豁免放疗取决于多个因素，包括患者的临床病理特征和个体情况。一般来说，保乳术后豁免放疗可能考虑在以下情况下：  1. **早期乳腺癌：** 保乳手术豁免放疗通常适用于早期乳腺癌，即病灶较小、未侵犯淋巴结或其他组织的情况。  2. **负面淋巴结：** 如果淋巴结检查结果为阴性，即未发现癌细胞转移至淋巴结，那么豁免放疗的可能性较大。  3. **雌激素受体阳性：** 如果肿瘤对雌激素敏感（雌激素受体阳性），患者可能更有资格豁免放疗，因为这类肿瘤对激素治疗的反应较好。  4. **年龄和整体健康状况：** 年轻患者或整体健康状况较好的患者可能更容易被考虑为豁免放疗，因为放疗可能对其它器官有一定的副作用。  5. **分子生物学因素：** 某些乳腺癌的分子生物学特征可能影响是否需要放疗，例如HER2/neu过表达的情况可能需要额外的治疗手段。  需要注意的是，最终的治疗方案应由乳腺癌专家根据患者的具体情况来制定。此外，医学领域的知识在不断更新，因此建议患者与医生进行详细的讨论，以制定最合适的治疗计划。 | 保乳术后是否需要放疗是基于一系列因素来决定的，包括患者的个人情况、肿瘤的生物学特征以及手术时对肿瘤的处理情况等。一般来说，如果患者符合以下条件，可能会考虑豁免放疗：  1. **早期乳腺癌：** 保乳手术通常适用于早期乳腺癌患者，即癌症尚未扩散到淋巴结或其他部位。  2. **肿瘤大小：** 肿瘤直径小于2厘米（T1N0M0）的患者通常具有较低的复发风险，因此可能不需要接受放疗。  3. **淋巴结状态：** 如果淋巴结检查结果显示未受累（N0），通常意味着癌症尚未扩散到淋巴系统，这些患者可能不需要放疗。  4. **肿瘤类型：** 某些生物学较温和的乳腺癌亚型，如雌激素受体阳性、HER2阴性的乳腺癌，可能在满足其他条件时可以考虑豁免放疗。  5. **负面边缘：** 如果手术时切除的肿瘤边缘均为负性，即没有癌细胞残留，也可能降低复发风险，从而不需要放疗。  6. **患者偏好：** 一些患者可能希望尽可能避免接受放疗的副作用和不便，因此在患者和医生共同决定下，可以考虑豁免放疗。  需要强调的是，以上条件并非绝对，具体的治疗方案应由专业医生根据患者的具体情况和最新的临床指南来确定。治疗方案的制定需要综合考虑患者的整体情况，以达到最佳的治疗效果和生活质量。 | 作为乳腺癌专家，对于乳腺癌的各种治疗方法及其后续管理有着深入的了解。保乳手术是乳腺癌早期患者常用的一种治疗方式，而放疗则通常是保乳手术后的辅助治疗手段，用以减少局部复发的风险。然而，并非所有保乳手术后的患者都需要接受放疗，某些情况下，患者可能符合豁免放疗的条件。  豁免放疗的条件通常基于患者的具体病情和一系列的临床病理特征。以下是一些可能考虑豁免放疗的情况：  年龄因素：一些年龄较大的患者，由于其预期寿命相对较短，且放疗的副作用可能对其生活质量产生较大影响，可能会考虑豁免放疗。但这一决定需要综合考虑患者的整体健康状况和具体病情。  病理特征：患者的肿瘤大小和分级、淋巴结状态等病理特征也是决定是否需要放疗的重要因素。一般来说，肿瘤较小、分级较低且淋巴结未受累的患者，豁免放疗的可能性较大。  手术切除范围：保乳手术中，如果切除的肿瘤边缘干净，即切缘阴性，且手术切除了足够范围的正常组织，也可能考虑豁免放疗。  患者的意愿和身体状况：患者的个人意愿和身体状况也是决定是否豁免放疗的重要考量。如果患者有强烈的豁免放疗意愿，且其身体状况不适合接受放疗，医生可能会在满足一定条件下考虑豁免放疗。  其他辅助治疗：一些患者可能接受了其他的辅助治疗，如激素治疗或化疗，这些治疗可能在一定程度上降低了局部复发的风险，从而影响了放疗的必要性。  需要注意的是，每个患者的情况都是独特的，豁免放疗的决定应该由医生根据患者的具体情况和最新的临床指南来做出。在做出决定之前，医生通常会与患者进行充分的沟通，解释放疗的利弊以及豁免放疗的可能风险。  总的来说，豁免放疗是一个需要综合考虑多种因素的复杂决策过程。患者应该与医生密切合作，充分了解所有可能的治疗选项和后果，以便做出最适合自己的治疗决策。 | 作为乳腺癌专家，我深知保乳手术（乳腺肿瘤切除术或乳腺区段切除术）后，放疗通常是减少局部复发风险的重要治疗手段。然而，在某些特定情况下，根据患者的具体病情和最新的临床研究结果，部分患者可能可以豁免放疗。以下是考虑豁免放疗时通常需要满足的条件：  患者的年龄：通常，年龄较大的患者（如65岁及以上）由于其较低的复发风险和可能存在的共病，可能更适合豁免放疗。  肿瘤大小和分期：肿瘤的大小和分期是决定是否需要放疗的关键因素。一般来说，肿瘤越小，分期越早（如T1a或T1b期），豁免放疗的可能性越大。  肿瘤的生物学特征：肿瘤的激素受体状态（如ER/PR阳性）和HER2状态也可能影响放疗的决策。某些低风险的生物学特征组合可能支持豁免放疗。  手术切缘情况：手术切缘的状态是评估肿瘤是否已被完全切除的重要指标。如果切缘干净（即切缘阴性），则可能考虑豁免放疗。  淋巴结状态：淋巴结是否受累也是决定放疗需求的重要因素。如果患者的淋巴结未受累（即N0期），则可能更容易豁免放疗。  患者的健康状况和偏好：患者的整体健康状况、对放疗的耐受性以及个人偏好也可能影响治疗决策。  多基因检测：一些多基因检测（如Oncotype DX或MammaPrint）可以帮助评估患者的复发风险，并可能指导是否需要放疗。  临床试验和最新研究：随着医学的不断进步，新的临床试验和研究成果可能为豁免放疗提供更多的依据。  需要注意的是，豁免放疗的决策应该是个体化的，并且需要由多学科团队（包括外科医生、放疗医生、病理医生和肿瘤内科医生）共同讨论决定。此外，即使患者满足了豁免放疗的条件，也应密切监测并定期接受复查，以确保肿瘤没有复发或转移。 | |
| 11. What are the common regimens for neoadjuvant therapy in triple-negative breast cancer? | Sure, I'd be happy to provide information on neoadjuvant therapy regimens for triple-negative breast cancer.  Neoadjuvant therapy, also known as preoperative or primary systemic therapy, is a treatment approach where systemic therapy is administered before surgery. This approach is commonly used in triple-negative breast cancer (TNBC) to shrink tumors before surgical removal. TNBC is known for its aggressive nature and lack of expression of estrogen receptor (ER), progesterone receptor (PR), and human epidermal growth factor receptor 2 (HER2).  1. **Chemotherapy Regimens:**  - **Anthracycline and Taxane-Based Regimens:** One of the commonly used neoadjuvant chemotherapy regimens for TNBC includes a combination of anthracycline (such as doxorubicin or epirubicin) and taxane (paclitaxel or docetaxel). This combination has shown effectiveness in reducing tumor size.   - **Carboplatin Addition:** Some regimens may include carboplatin, especially in patients with BRCA mutations, as TNBC with BRCA mutations may be more responsive to platinum-based chemotherapy.   - **Cisplatin:** Cisplatin-based regimens are also considered in some cases, particularly in patients where anthracyclines or taxanes may not be suitable.  2. **Immunotherapy:**  - **PD-1/PD-L1 Inhibitors:** Immune checkpoint inhibitors, such as pembrolizumab or atezolizumab, have shown promise in treating TNBC. They are often used in combination with chemotherapy in the neoadjuvant setting.  3. **PARP Inhibitors:**  - For patients with TNBC and BRCA mutations, poly ADP-ribose polymerase (PARP) inhibitors like olaparib or talazoparib may be considered.  4. **Clinical Trials:**  - Ongoing clinical trials may explore novel therapeutic approaches, and participation in such trials can be considered for eligible patients.  5. **HER2-Directed Therapy:**  - Although TNBC is characterized by the absence of HER2 expression, in some cases, there may be a small subset of tumors that show low-level HER2 expression. In such cases, HER2-directed therapies might be considered.  It's crucial to note that the choice of neoadjuvant therapy is individualized based on various factors such as the stage of the disease, patient's overall health, and specific molecular characteristics of the tumor. The landscape of breast cancer treatment is continuously evolving, and consulting with an oncologist who can tailor the treatment plan to the individual patient is essential. | similar | 三阴性乳腺癌的新辅助治疗方案通常包括多种治疗方法，根据患者的具体情况和病理特征来制定个性化的治疗方案。以下是一些常见的三阴性乳腺癌新辅助治疗方案：  1. **化疗（Chemotherapy）**：  - 化疗是三阴性乳腺癌新辅助治疗的主要方式之一。常用的化疗药物包括阿霉素类、紫杉醇类等，这些药物可以通过杀死癌细胞来缩小肿瘤的体积。  2. **手术治疗**：  - 新辅助治疗可能在手术前进行，以缩小肿瘤的体积，使手术更容易实施。手术的方式可能包括乳腺保留手术或乳腺切除术。  3. **放疗（Radiation Therapy）**：  - 放疗通常在手术后进行，但在新辅助治疗中，它有时也可能在手术前使用，特别是在肿瘤较大或侵袭性较强的情况下。  4. **靶向治疗**：  - 针对三阴性乳腺癌的特定分子靶点，例如PARP抑制剂、PD-1/PD-L1抑制剂等，是近年来的研究热点。这些治疗方法有望提供更为精准和有效的治疗。  5. **激素治疗**：  - 由于三阴性乳腺癌不表达雌激素受体、孕激素受体和HER2受体，传统的激素治疗对其无效。因此，激素治疗在这类乳腺癌中并不常见。  治疗方案的选择会受到患者年龄、肿瘤大小、淋巴结状况等多方面因素的影响。最佳的治疗方案需要由一支多学科的医疗团队共同制定，以确保患者能够获得最合适的个体化治疗。如果有具体的患者信息或病例细节，可以更详细地定制治疗方案。 | similar | 作为乳腺癌专家，深知三阴性乳腺癌的复杂性和治疗的挑战性。三阴性乳腺癌是指癌组织免疫组织化学检查结果为雌激素受体（ER）、孕激素受体（PR）和原癌基因Her-2均为阴性的乳腺癌。这种类型的乳腺癌通常对激素治疗和针对Her-2的靶向治疗不敏感，因此治疗方案的选择相对有限。  新辅助治疗通常指的是在手术前给予的治疗，旨在缩小肿瘤、降低临床分期，从而提高手术的成功率和患者的生存率。对于三阴性乳腺癌，新辅助治疗的常见方案主要包括化疗和免疫治疗。  化疗： 蒽环类药物和紫杉类药物联合使用是常用的化疗方案，如AC（阿霉素+环磷酰胺）到T（紫杉醇）的方案。 其他可能使用的药物包括铂类药物（如顺铂）、5-氟尿嘧啶、卡培他滨等。 这些药物可以单独使用，也可以联合使用，具体取决于患者的具体情况和医生的判断。 免疫治疗： 近年来，免疫治疗在乳腺癌治疗中取得了显著进展，尤其是对于三阴性乳腺癌。 PD-1/PD-L1抑制剂和CTLA-4抑制剂等免疫检查点抑制剂在某些患者中显示出良好的疗效。 然而，需要注意的是，并非所有三阴性乳腺癌患者都适合免疫治疗，因此需要进行相应的生物标志物检测来预测疗效。 除了上述治疗方案外，还有一些新兴的治疗方法正在研究中，如靶向治疗针对三阴性乳腺癌特有的基因突变或通路等。然而，目前这些方法仍处于临床试验阶段，尚未广泛应用于临床。  总之，三阴性乳腺癌的新辅助治疗方案需要根据患者的具体情况进行个体化选择。在制定治疗方案时，医生会综合考虑患者的年龄、身体状况、肿瘤大小和位置以及患者的偏好等因素。同时，患者需要密切关注治疗过程中的副作用和疗效，及时调整治疗方案以达到最佳治疗效果。 | 三阴性乳腺癌（TNBC）是一种特殊类型的乳腺癌，其特点是雌激素受体（ER）、孕激素受体（PR）和人表皮生长因子受体2（HER2）均为阴性。由于这些受体阴性，三阴性乳腺癌对于许多常规乳腺癌治疗药物（如激素治疗和HER2靶向药物）不敏感，因此治疗起来更具挑战性。  新辅助治疗是指在手术前给予的治疗，目的是缩小肿瘤、降低肿瘤分期，从而提高手术的成功率和患者的生存率。对于三阴性乳腺癌，新辅助治疗尤为重要。  以下是三阴性乳腺癌新辅助治疗的常见方案：  化疗：由于三阴性乳腺癌对激素治疗不敏感，化疗通常是其主要的治疗方式。常用的化疗药物包括蒽环类药物（如多柔比星）、紫杉类药物（如紫杉醇、多西他赛）以及铂类药物（如顺铂、卡铂）等。这些药物可以单独使用，也可以联合使用，具体方案需根据患者的具体情况和医生的建议来确定。 免疫治疗：近年来，免疫治疗在三阴性乳腺癌的治疗中逐渐展现出潜力。一些免疫检查点抑制剂（如PD-1/PD-L1抑制剂）正在临床试验中，并已取得了一些令人鼓舞的结果。 靶向治疗：尽管三阴性乳腺癌缺乏常见的激素受体和HER2靶点，但研究人员仍在寻找其他可能的靶点。例如，针对某些特定的基因突变或通路异常，可能会开发出新的靶向治疗药物。目前，一些针对PARP、血管生成等靶点的药物正在临床试验中。 局部治疗：在某些情况下，新辅助治疗可能还包括局部治疗，如放疗。放疗可以在手术前或手术后进行，旨在减小肿瘤的大小或消灭可能残留的癌细胞。 需要强调的是，每个患者的情况都是独特的，因此治疗方案应根据患者的具体情况、肿瘤的生物学特征以及医生的建议来制定。同时，患者在治疗过程中应保持良好的心态，积极配合医生的治疗，以期获得最佳的治疗效果。 | |
| 12. What are your basic principles for the treatment of metastatic breast cancer? | I'm not a specialized breast cancer specialist, but I can certainly provide some general principles that are commonly followed in the treatment of metastatic breast cancer. Keep in mind that specific recommendations may vary based on individual cases, and it's essential for patients to consult with their healthcare team for personalized advice.  1. **Multidisciplinary Approach:**  - Metastatic breast cancer often requires a team of specialists, including medical oncologists, radiation oncologists, and surgeons, to develop a comprehensive treatment plan.  2. **Systemic Therapies:**  - **Chemotherapy:** Depending on the subtype of breast cancer, chemotherapy may be used to target rapidly dividing cancer cells throughout the body.  - **Hormone Therapy:** For hormone receptor-positive breast cancer, hormone therapy can be effective in blocking hormone signals that fuel cancer growth.  - **Targeted Therapies:** Specific targeted therapies may be employed based on the presence of certain molecular markers, such as HER2-positive breast cancer.  3. **Immunotherapy:**  - Emerging as a promising area of research, immunotherapy aims to enhance the body's immune system to recognize and attack cancer cells.  4. **Palliative Care:**  - Focuses on improving the quality of life for patients, managing symptoms, and providing emotional support. It's an integral part of metastatic breast cancer care.  5. **Clinical Trials:**  - Participation in clinical trials can provide access to innovative treatments and contribute to advancing the understanding of metastatic breast cancer.  6. **Regular Monitoring:**  - Close monitoring through imaging studies and other tests helps assess the response to treatment and make necessary adjustments.  7. **Patient Education and Support:**  - Empowering patients with information about their condition and involving them in decision-making can be crucial. Support groups and counseling services also play a significant role.  8. **Personalized Medicine:**  - Advances in molecular profiling allow for more personalized treatment approaches, tailoring therapies to the specific characteristics of the individual's cancer.  Remember, these principles serve as a general framework, and the specifics will depend on factors like the type and stage of breast cancer, the patient's overall health, and individual preferences. Always consult with a healthcare professional for the most accurate and personalized advice. | simialr | 嗨！当然，我会尽力提供有关转移性乳腺癌治疗的基本原则。请记住，以下建议可能需要根据患者的具体情况进行调整，最好由专业医生进行个性化评估。  1. **病理评估和分期：** 首先，了解病理学特征和疾病分期对于确定治疗方案至关重要。这有助于医生了解肿瘤的生物学行为和扩散程度。  2. **手术治疗：** 在可能的情况下，手术是治疗转移性乳腺癌的一部分。手术的类型可能包括乳腺切除术、淋巴结清扫或其他相关程序，目的是最大限度地切除癌组织。  3. **放疗：** 放射疗法可用于减少残余癌细胞的数量，特别是在手术后。它也可以用于缓解症状，如骨转移时的骨痛。  4. **系统治疗：** 这包括化疗、内分泌治疗和靶向治疗等方法，旨在通过全身性手段杀死或抑制癌细胞的生长。  5. **个体化治疗：** 针对乳腺癌的分子亚型进行精准治疗。某些亚型对荷尔蒙疗法或靶向药物可能更为敏感。  6. **临床试验：** 对于转移性乳腺癌，参与临床试验可能是一种选择，因为这提供了新治疗方法的机会。  7. **支持性治疗：** 转移性乳腺癌的治疗不仅仅关乎抗癌手段，还包括对患者身心健康的支持，如疼痛管理、心理支持等。  请注意，以上只是一般性建议，具体的治疗计划应该根据患者的病情和其他相关因素而定。最好的建议是与专业医生一起讨论患者的具体情况，以制定最合适的治疗方案。 | similar | 作为乳腺癌专家，深知转移性乳腺癌的治疗是一个复杂且需要多学科合作的过程。治疗的基本原则主要包括以下几个方面：  个体化治疗：每位患者的情况都是独特的，因此治疗方案需要根据患者的具体病情、身体状况、年龄、生活习惯等因素进行个体化制定。例如，对于ER（雌激素受体）和/或PR（孕激素受体）阳性的患者，内分泌治疗通常是一个有效的选择；而对于HER2（人类表皮生长因子受体2）过表达的患者，抗HER2治疗则是关键。 综合治疗：转移性乳腺癌的治疗往往需要结合多种治疗手段，如手术、化疗、放疗、内分泌治疗、生物免疫治疗等。这些手段的选择和组合需要根据患者的病情和医生的建议进行。 控制疾病进展：转移性乳腺癌的治疗目标之一是控制疾病的进展，延长患者的生存期。这通常需要通过持续的治疗和监测来实现。 提高生活质量：在治疗过程中，关注患者的生活质量同样重要。医生需要努力减轻患者的症状，提高患者的生活质量，同时也要关注患者的心理健康。 持续监测和调整治疗方案：由于转移性乳腺癌的病情可能会随着时间的推移而发生变化，因此患者需要接受持续的监测，并根据病情的变化及时调整治疗方案。 参与临床试验：对于某些患者，参与临床试验可能是一个合适的选择。通过参与临床试验，患者可以获得最新的治疗方法，并为乳腺癌的研究做出贡献。 总之，转移性乳腺癌的治疗是一个复杂且需要持续关注的过程。在制定治疗方案时，医生会综合考虑患者的病情、身体状况、生活习惯等因素，力求为患者提供最佳的治疗效果和生活质量。同时，患者也需要积极参与治疗过程，与医生保持良好的沟通，共同应对这一挑战。 | similar | |
| 13. What are your recommendations for the management of bone health in patients during adjuvant endocrine therapy? | Certainly! It's crucial to address bone health in patients undergoing adjuvant endocrine therapy, especially since these treatments may impact bone density. Here are some recommendations:  1. **Calcium and Vitamin D Supplementation:**  - Ensure an adequate intake of calcium and vitamin D. These are essential for maintaining bone health.  - Calcium requirements may vary, but a general guideline is around 1000 to 1200 mg per day. Vitamin D intake should be sufficient to maintain blood levels in the normal range.  2. **Regular Bone Density Monitoring:**  - Conduct baseline bone density assessments before initiating endocrine therapy.  - Follow-up with periodic monitoring to detect any decline in bone density. This helps in early intervention if necessary.  3. **Lifestyle Modifications:**  - Encourage weight-bearing exercises, as they stimulate bone formation. This includes activities like walking, jogging, or weight training.  - Discourage smoking and excessive alcohol intake, as they can negatively impact bone health.  4. **Bisphosphonate Therapy:**  - Consider the use of bisphosphonates in certain cases, especially if the patient is at high risk of osteoporosis or fracture.  - Bisphosphonates, like alendronate or zoledronic acid, can help maintain or improve bone density.  5. **Individualized Risk Assessment:**  - Assess the patient's individual risk factors for bone-related complications.  - Factors such as age, menopausal status, previous fractures, and family history contribute to the overall risk profile.  6. **Hormone Replacement Therapy (HRT) Consideration:**  - Discuss the potential benefits and risks of hormone replacement therapy with the patient.  - In some cases, HRT may be considered to mitigate the bone-related effects of endocrine therapy.  7. **Collaboration with Bone Health Specialists:**  - Consider involving a bone health specialist, such as an endocrinologist or rheumatologist, in the management of bone health.  - This collaboration can provide a more comprehensive approach to address bone-related concerns.  It's crucial to note that individual patient characteristics and the specific endocrine therapy being used can influence the approach to managing bone health. Therefore, decisions should be personalized based on the patient's unique circumstances. Regular communication between the oncology team and other specialists can optimize the overall care for these patients. | similar | 首先，要注意在乳腺癌治疗中，特别是在进行辅助内分泌治疗期间，患者的骨健康管理至关重要。辅助内分泌治疗通常包括使用激素阻断剂，如Tamoxifen或Aromatase抑制剂，这可能对骨密度产生一些影响。  以下是一些建议：  1. **骨密度监测：** 建议定期进行骨密度检测，以评估治疗对患者骨骼的影响。这可以帮助及早发现任何骨健康问题。  2. **鈣和维生素D补充：** 确保患者摄取足够的钙和维生素D。这对于维持骨密度和骨健康至关重要。如果患者的饮食无法满足这些需求，可能需要考虑补充。  3. **运动：** 鼓励适度的体育锻炼，特别是重力负荷的活动，如步行、慢跑或举重。这有助于维持骨密度和强度。  4. **避免骨折风险因素：** 帮助患者识别和减少骨折的风险因素，例如提供关于安全行走和防摔的建议。  5. **药物治疗：** 在一些情况下，医生可能会考虑使用骨密度增强药物，如双膦酸类药物，以降低骨折风险。这应该在医生的监督下进行。  6. **监测和调整治疗：** 定期监测患者的骨密度和整体骨健康，并根据需要调整治疗计划。  请注意，这些建议是基于一般情况的，并且具体的治疗方案可能因个体情况而异。患者应该与他们的医疗团队合作，根据个人的状况和需求制定最适合他们的骨健康管理计划。 | similar | 作为乳腺癌专家，深知辅助内分泌治疗在乳腺癌患者综合管理中的重要性，同时也清楚这一治疗手段可能会对患者的骨健康产生一定的不良影响。以下是对于辅助内分泌治疗期间患者骨健康管理的一些专业建议：  了解骨质疏松的风险：首先，患者和医生都应认识到辅助内分泌治疗可能增加骨质疏松和骨折的风险。这是因为某些内分泌治疗药物，如芳香化酶抑制剂和选择性雌激素受体调节剂，可能会降低雌激素水平，从而影响骨密度。 定期监测骨密度：建议患者在开始内分泌治疗前进行基线的骨密度检测，并在治疗期间定期重复检测，以便及时发现并跟踪骨密度的变化。 生活方式的调整：鼓励患者通过饮食和锻炼来维护骨骼健康。富含钙和维生素D的均衡饮食对骨骼有益。此外，负重锻炼和力量训练可以增强肌肉和骨骼，减少跌倒和骨折的风险。 考虑钙和维生素D的补充：根据患者的具体情况和医生的建议，可能需要补充钙和维生素D。这些营养素对于骨骼健康至关重要，但补充的剂量需根据个体情况而定。 药物治疗：对于已经出现骨质疏松或骨密度明显降低的患者，医生可能会考虑使用抗骨质疏松药物，如双膦酸盐或地诺单抗。这些药物可以帮助增强骨骼，减少骨折的风险。 预防跌倒：除了直接关注骨骼健康外，预防跌倒也是减少骨折风险的重要措施。患者可以通过改善家居环境（如增加照明、减少杂物）和使用辅助设备（如手杖、防滑垫）来降低跌倒的可能性。 持续沟通与监测：在治疗期间，患者应与医生保持密切沟通，及时报告任何骨骼相关的不适或问题。医生也应根据患者的具体情况和治疗反应，调整治疗方案和骨健康管理策略。 综上所述，辅助内分泌治疗期间患者骨健康管理是一个多方面的过程，需要患者和医生的共同努力和持续关注。通过综合应用上述策略，可以有效降低骨质疏松和骨折的风险，提高患者的生活质量和治疗预后。 | similar | |
| 14. For young female breast cancer patients with HR-positive tumors who express a desire for fertility, what considerations do you have in the treatment plan? | Certainly! When addressing the treatment plan for young female breast cancer patients with Hormone Receptor (HR)-positive tumors who express a desire for fertility, several considerations come into play. Let's delve into the specifics:  1. **Fertility Preservation Options:**  - It's crucial to discuss and explore fertility preservation options before starting cancer treatment. This may include egg or embryo freezing.  - Coordination with a reproductive specialist is essential to guide patients through fertility preservation procedures.  2. **Hormone Receptor-Positive (HR+) Tumors:**  - HR+ tumors typically respond well to hormonal therapies. Discussing the potential impact of these treatments on fertility is important.  - The choice of hormonal therapy and its duration should be balanced with the patient's fertility goals.  3. **Chemotherapy Considerations:**  - Chemotherapy can have varying effects on fertility. Discuss the specific chemotherapy drugs being considered and their potential impact on ovarian function.  - Some chemotherapy regimens may pose a higher risk of infertility, and alternatives or modifications might be considered.  4. **Timing of Treatment and Fertility Interventions:**  - Coordination between the oncology and reproductive medicine teams is crucial to optimize the timing of fertility interventions and cancer treatment.  - Delays in cancer treatment to pursue fertility preservation should be minimized to ensure optimal oncologic outcomes.  5. **Risk of Recurrence and Long-Term Hormonal Therapy:**  - Balancing the risk of cancer recurrence with the desire for fertility is a critical aspect.  - Long-term hormonal therapy, if recommended, may impact fertility, and alternative strategies or breaks in therapy could be discussed based on individual cases.  6. **Patient Education and Shared Decision-Making:**  - Provide comprehensive education on the potential impact of cancer treatments on fertility and the available fertility preservation options.  - Engage in shared decision-making, considering the patient's values, priorities, and the medical context.  7. **Supportive Care and Psychosocial Considerations:**  - Address the emotional and psychological aspects of fertility preservation and cancer treatment.  - Offer appropriate support services, such as counseling and support groups, to help patients cope with the emotional challenges.  8. **Follow-Up and Monitoring:**  - Establish a follow-up plan to monitor the patient's response to treatment, fertility status, and overall well-being.  - Periodic reevaluation of fertility goals and adjustments to the treatment plan may be necessary based on the evolving clinical situation.  Remember, individualized care is crucial in these cases, and decisions should be made collaboratively with the patient, considering both their oncologic and fertility goals. | similar | 针对HR阳性年轻女性乳腺癌患者，如果她们有生育要求，诊疗计划需要考虑多个方面，以确保在治疗乳腺癌的同时最大程度地保留生育能力和维持患者的健康。以下是一些考虑因素：  1. **治疗选择**：在制定治疗计划时，需要选择对乳腺癌有效的治疗方法，但也要考虑这些治疗对生育能力的影响。通常，手术、放疗、化疗和内分泌治疗等方法都可能对生育能力产生不同程度的影响。  2. **手术考量**：保留乳房的手术（如保乳手术）可能对患者的心理健康和生活质量产生积极影响，但可能会影响乳腺组织的结构，从而影响未来的哺乳能力。这需要在患者和医生之间进行充分的讨论和决策。  3. **化疗和放疗**：化疗和放疗可能会对卵巢功能产生负面影响，从而影响生育能力。在制定治疗计划时，医生需要权衡治疗的必要性和可能带来的生育影响，并在可能的情况下采取措施来保护卵巢功能。  4. **内分泌治疗**：对于HR阳性的乳腺癌患者，内分泌治疗是常见的治疗选择。然而，某些内分泌治疗可能会导致月经不规律甚至停经，影响生育能力。在这种情况下，可能需要考虑辅助生育技术或者暂时中断内分泌治疗来实现生育愿望。  5. **生育规划和咨询**：对于有生育意愿的患者，需要提供全面的生育规划和咨询服务。这包括评估患者的生育状况、讨论治疗选择的影响以及提供必要的支持和建议，以确保患者在治疗过程中了解自己的选择，并做出符合自己意愿和健康状况的决定。  6. **监测和随访**：对于完成治疗的患者，需要定期进行监测和随访，以确保其身体健康和生育功能的恢复情况。在随访过程中，医生需要关注患者的生育愿望和生育能力恢复情况，并提供必要的支持和指导。  总体而言，针对HR阳性年轻女性乳腺癌患者的生育要求，诊疗计划需要综合考虑患者的治疗需求、生育愿望以及可能的生育影响，以达到在保证治疗效果的前提下最大程度地满足患者的生育需求。因此，医生和患者之间的沟通和共同决策非常重要。 | similar | 作为乳腺癌专家，面对HR阳性（激素受体阳性，通常是指雌激素受体ER和/或孕激素受体PR阳性）的年轻女性乳腺癌患者，特别是有生育要求的患者，我会在诊疗计划中做如下特殊考量：  保护卵巢功能：对于这类患者，保护卵巢功能至关重要，因为激素受体阳性的乳腺癌可能与患者的内分泌系统有关。在治疗过程中，我会尽可能选择对卵巢功能影响较小的治疗方案，如使用新一代的化疗药物或调整药物剂量，以减少对卵巢的损害。 内分泌治疗策略：由于HR阳性乳腺癌对激素治疗敏感，内分泌治疗是重要的治疗手段。然而，传统的内分泌治疗药物可能会影响患者的生育能力。因此，我会考虑使用短期内分泌治疗方案，或者在完成必要的治疗后，为患者安排一段时间的生育窗口。 生育力保存咨询：在治疗开始前，我会建议患者咨询生育力保存专家，了解并考虑如卵子冷冻、胚胎冷冻等生育力保存技术。这些技术可以在治疗前保存患者的生育能力，以便在治疗结束后实现生育愿望。 治疗顺序和时间安排：对于需要接受多种治疗（如手术、化疗、放疗和内分泌治疗）的患者，我会仔细安排治疗的顺序和时间，以最大限度地减少对生育能力的影响。例如，可以先进行手术治疗，然后进行化疗和放疗，最后进行内分泌治疗，以便为患者留出更多的时间来考虑生育问题。 心理支持和咨询：面对乳腺癌的诊断和治疗，患者可能会感到焦虑和恐惧。对于有生育需求的患者来说，这种心理压力可能会更大。因此，我会为患者提供心理支持和咨询，帮助她们更好地应对疾病和治疗带来的挑战。 长期随访和监测：在完成治疗后，我会为患者安排长期的随访和监测计划，以确保疾病没有复发或转移。在随访过程中，我也会关注患者的生育需求和生育能力恢复情况，并根据需要调整治疗策略。 总之，对于HR阳性的年轻女性乳腺癌患者，特别是有生育需求的患者，我会在诊疗计划中综合考虑患者的生育需求、疾病特点和治疗手段的影响，制定个性化的治疗方案，以最大限度地保护患者的生育能力和提高治疗效果。 | similar | |
| 15. How should long-term follow-up and monitoring be conducted for breast cancer patients? | Certainly! Regular follow-up and monitoring are crucial components of breast cancer survivorship care. The approach may vary based on individual cases, but here are general guidelines:  1. **Frequency of Follow-up Visits:**  - **First Two Years:** Frequent visits, typically every 3-6 months.  - **Years 3-5:** Visits may be less frequent, every 6-12 months.  - **After 5 Years:** Annual visits are common.  2. **Medical History and Physical Examination:**  - Regularly review the patient's medical history, paying attention to any new symptoms or concerns.  - Conduct a thorough physical examination, with a focus on the breast area, to check for any signs of recurrence.  3. **Imaging Studies:**  - Mammograms may continue annually, especially for the opposite breast.  - Other imaging studies, such as ultrasounds or MRIs, may be recommended based on the patient's specific situation.  4. **Tumor Markers:**  - Some breast cancer subtypes have associated tumor markers (e.g., CA 15-3, CA 27.29). While not perfect, these markers can sometimes aid in monitoring.  5. **Bone Health:**  - Assess and monitor bone health, especially if the patient has been on certain therapies that may impact bone density.  6. **Assessment of Treatment Side Effects:**  - Check for any lingering side effects from previous treatments, addressing them appropriately.  7. **Psychosocial Support:**  - Consider the emotional well-being of the patient and provide access to support groups or counseling services.  8. **Genetic Counseling:**  - For patients with a family history or known genetic mutations, regular counseling and testing may be necessary.  9. **Lifestyle and Wellness:** | similar | 首先，我要强调我不是医生，但我可以为你提供一些关于乳腺癌患者长期随访和监测的一般性建议。请务必在实际情况中咨询专业医生以获取个性化的建议。  长期随访和监测对于乳腺癌患者的康复和预防复发至关重要。以下是一些建议：  1. **定期体检：** 患者应该定期接受乳腺体检，包括乳腺自检和定期的临床检查。这有助于早期发现任何异常。  2. **影像学检查：** 医生可能会建议进行定期乳腺X光摄影（乳腺X光）或其他影像学检查，如乳腺超声或核磁共振成像。这些检查有助于监测肿瘤的生长和变化。  3. **血液检查：** 有时医生可能会建议进行一些血液检查，以监测肿瘤标志物或检查其他与癌症相关的指标。  4. **临床评估：** 医生会进行全面的临床评估，包括了解患者的症状、身体状况和生活质量等方面。这有助于提前发现潜在问题。  5. **心理支持：** 乳腺癌患者长期随访中，心理健康同样重要。提供专业的心理支持，帮助患者应对可能的情绪和心理压力。  6. **药物治疗：** 如果患者正在接受药物治疗，医生会监测治疗的效果和任何潜在的副作用。患者应按医嘱规定正确服药。  7. **生活方式管理：** 长期随访中，医生可能会关注患者的生活方式，包括饮食、运动和戒烟等。这有助于提高康复的成功率。  总体而言，乳腺癌患者的长期随访应该是个性化的，并基于患者的具体情况和治疗方案进行调整。患者应定期与医疗团队进行沟通，分享任何疑虑或变化，以确保获得最佳的医疗关怀。 | 你好！了解到乳腺癌患者的长期随访和监测对于确保他们的健康非常关键。下面是一些建议，但请注意这只是一般性的指导，具体的随访计划可能会因患者的个体差异而有所不同。  1. **手术后的初期阶段：**  - 在手术后的最初几周内，随访通常会更为频繁，以确保患者适应手术后的康复和生活。  - 通过体检和相关检查来监测伤口愈合情况，并评估患者的整体健康状况。  2. **放疗或化疗后：**  - 如果患者接受了放疗或化疗，随访计划可能会更为密集，以监测患者的反应和副作用。  - 定期进行血液检查和影像学检查，以评估治疗的效果。  3. **长期随访：**  - 随着时间的推移，随访的频率可能会减少，但仍应定期进行。  - 这期间的随访可能包括定期的临床检查、乳腺摄影、超声检查或其他必要的检查。  4. **个性化治疗计划：**  - 长期随访计划应该是个性化的，根据患者的具体状况和治疗历史进行调整。  - 针对患者的生活方式、遗传因素和其他相关因素，制定相应的监测方案。  5. **心理健康支持：**  - 在整个随访过程中，心理健康的支持同样重要。患者可能经历焦虑、抑郁或其他情绪问题，及时介入是必要的。  6. **乳腺影像学监测：**  - 定期进行乳腺摄影和其他影像学检查，以便及早发现任何复发或新的病变。  7. **关注转移风险：**  - 特别关注乳腺癌的转移风险，可能需要进行骨密度扫描等检查，以排除骨转移的可能性。  请记住，以上建议是基于一般情况的指导。实际的随访计划应该由乳腺癌专业医生根据患者的具体情况 | 作为乳腺癌专家，深知长期随访和监测对于乳腺癌患者的重要性。以下是一些关于如何进行乳腺癌患者的长期随访和监测的建议：  一、临床体检  临床体检是乳腺癌患者长期随访和监测的基础。建议患者至少每年进行一次全面的身体检查，包括乳房和腋窝淋巴结的检查。对于有高风险因素的患者，如年轻、家族史等，可以增加体检的频率。  二、乳腺影像学检查  乳腺X线摄影（钼靶检查）：这是乳腺癌筛查和监测的常用方法。对于一般风险的患者，建议每年进行一次乳腺X线摄影。对于高风险患者，如BRCA基因突变携带者，可能需要增加检查的频率或使用其他影像学方法，如乳腺磁共振成像（MRI）。 乳腺超声检查：超声检查对于评估乳腺肿块的性质、引导穿刺活检等方面具有重要价值。在随访过程中，可以根据需要进行超声检查。 乳腺MRI：对于高风险患者或具有特定临床情况的患者，乳腺MRI可以提供更敏感的检测手段。但考虑到其成本和假阳性率较高，通常不作为常规监测手段。 三、实验室检查  包括肿瘤标志物等实验室检查可以作为辅助监测手段。但需要注意的是，这些指标并不是特异性的，其升高并不一定意味着肿瘤复发或转移。因此，在解读实验室检查结果时，需要结合患者的临床情况进行综合判断。  四、生活方式和心理支持  在随访过程中，应关注患者的生活方式，包括饮食、运动、睡眠等，并给予必要的指导。同时，提供心理支持，帮助患者调整心态，积极面对疾病和治疗带来的挑战。  五、关注并发症和副作用  乳腺癌治疗可能导致一系列并发症和副作用，如淋巴水肿、骨质疏松、心脏毒性等。在随访过程中，应密切关注这些问题的出现，并及时采取措施进行干预和治疗。  六、多学科协作  乳腺癌患者的长期随访和监测需要多学科团队的协作，包括乳腺外科医生、肿瘤内科医生、放疗科医生、影像科医生、病理科医生以及护士等。通过多学科讨论和协作，可以为患者制定更加全面和个性化的随访和监测方案。 | similar | |
|  | - Emphasize healthy lifestyle choices, including exercise, a balanced diet, and avoiding tobacco and excessive alcohol consumption.  10. **Surveillance for Second Cancers:**  - Some breast cancer survivors may be at increased risk for developing other cancers. Tailor surveillance accordingly.  11. **Individualized Approach:**  - Recognize that each patient's journey is unique. Tailor the follow-up plan based on the initial stage of cancer, type of treatment received, and any specific risk factors.  12. **Shared Decision-Making:**  - Involve the patient in decision-making regarding the follow-up plan. Consider their preferences and address any concerns they may have.  It's essential to note that these recommendations can evolve based on emerging research and advancements in breast cancer care. Therefore, it's crucial for patients to stay informed and engage in ongoing discussions with their healthcare team. Regular communication and a personalized approach are key to effective long-term follow-up and monitoring for breast cancer survivors. |  |  | 和治疗方案进行制定。患者在整个过程中的积极参与和定期随访对于确保乳腺癌的有效监测和管理至关重要。如果有任何疑虑或症状，患者应及时向医生报告。 | 总之，乳腺癌患者的长期随访和监测是一个综合性的过程，需要临床体检、影像学检查、实验室检查等多种手段的结合。同时，关注患者的生活方式、心理状态以及并发症和副作用的出现，通过多学科协作，为患者提供全面、个性化的长期随访和监测服务。 |  | |

***: The fixed format of large language model response, which has no practical significance.**
