# Supplementary figures and images for: Large Language Models as a Consulting Hotline for Patients With Breast Cancer and Specialists in China: Cross-Sectional Questionnaire Study
Source: JMIR Med Inform. 2025 May 27;13:e66429. doi: 10.2196/66429 (PMC12133073; doi:10.2196/66429)

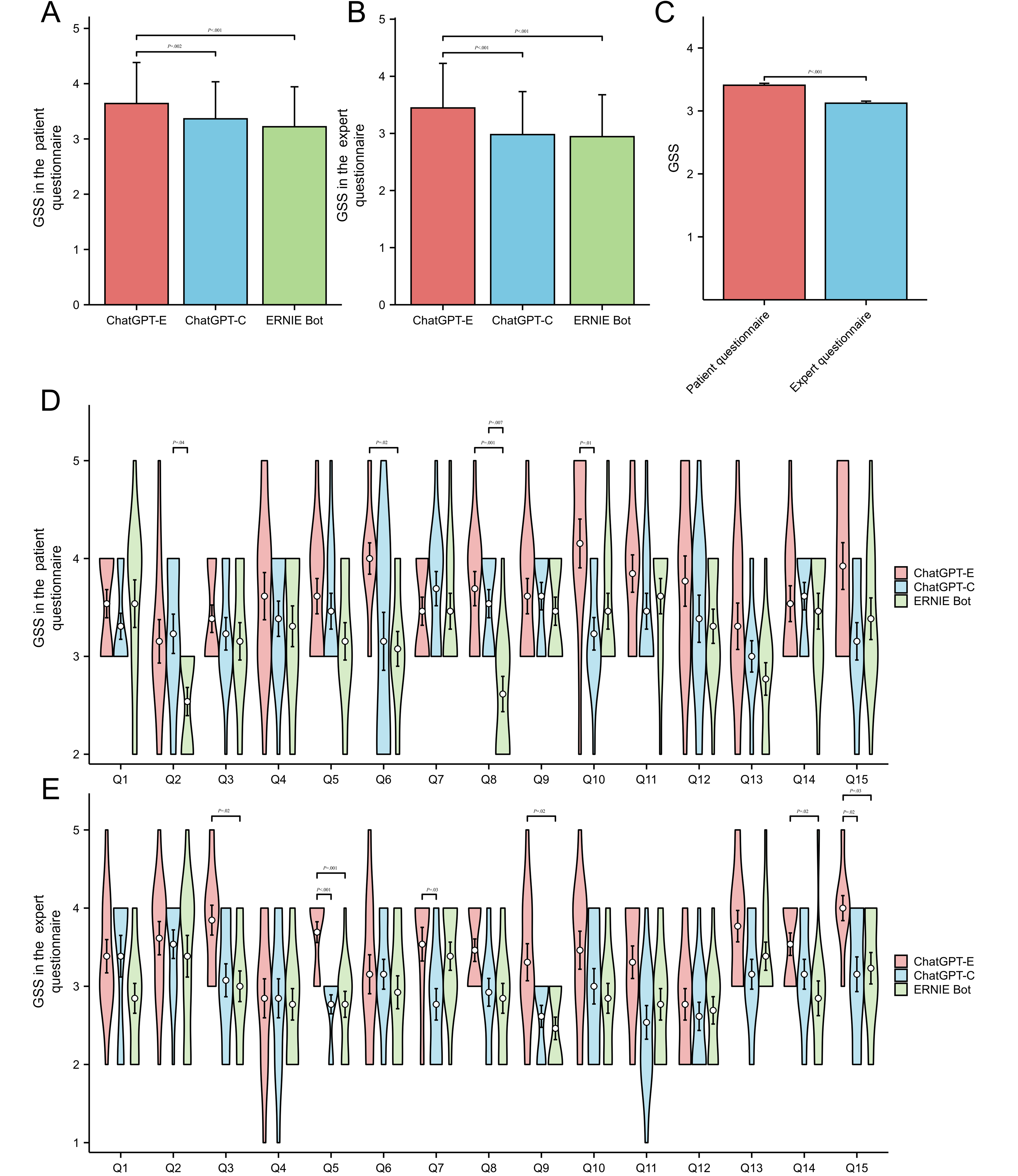

Supplement: Multimedia Appendix 2 [file medinform-v13-e66429-s002.png]

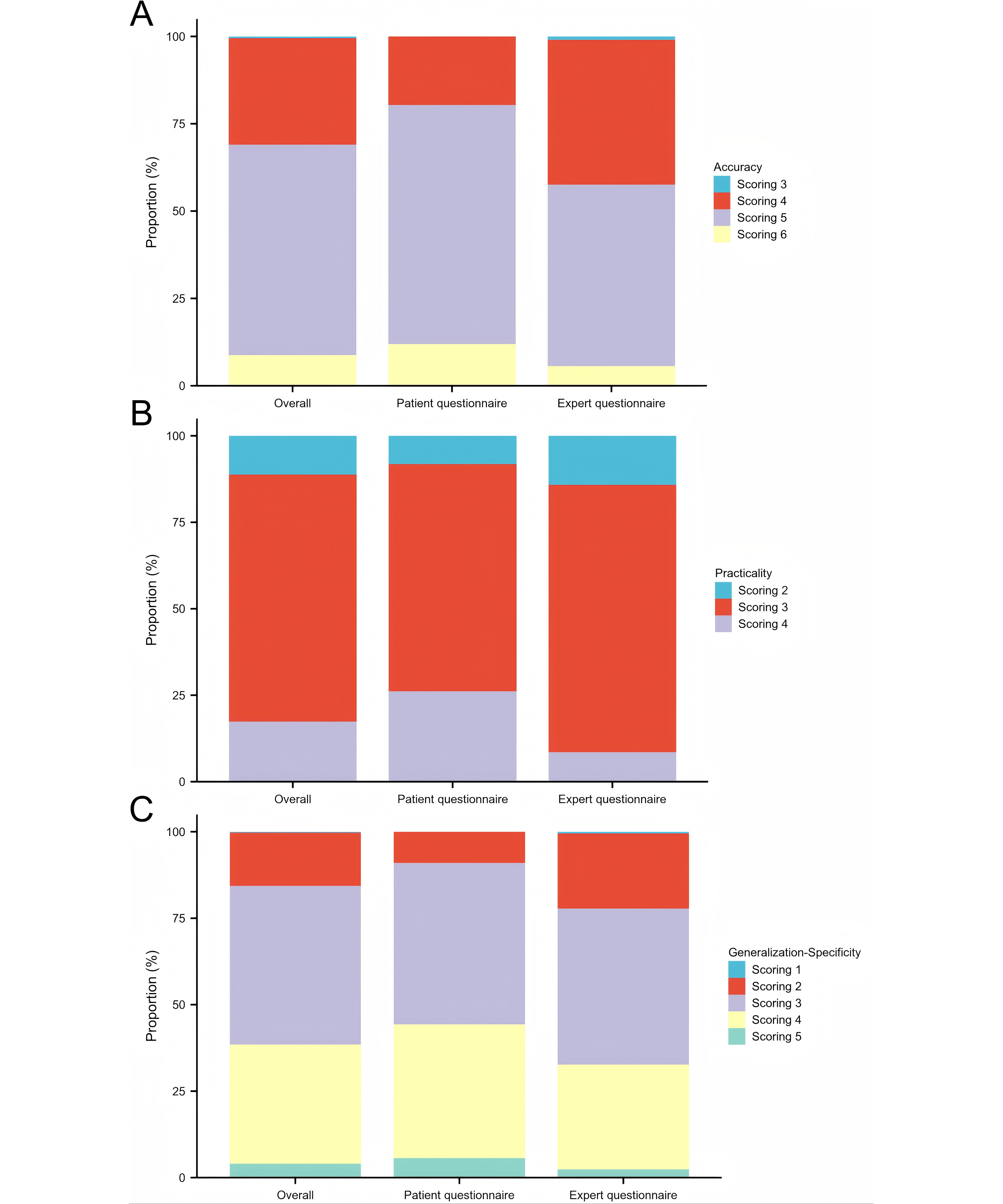

Supplement: Multimedia Appendix 3 [file medinform-v13-e66429-s003.png]
