# Supplementary material for: Large Language Models as a Consulting Hotline for Patients With Breast Cancer and Specialists in China: Cross-Sectional Questionnaire Study
Source: JMIR Med Inform. 2025 May 27;13:e66429. doi: 10.2196/66429 (PMC12133073; doi:10.2196/66429)
Supplement: Multimedia Appendix 4 [file medinform-v13-e66429-s004.docx]

**Supplementary table S2.** Comparison of overall accuracy, practicality and Generalization-Specificity Score (GSS) between patient and expert questionnaires using Mann-Whitney U Test.

| **Category** | **Group I (Patient questionnaire)** | **Group J (Expert questionnaire)** | **Mann-Whitney U Statistic** | **Difference (J-I)** | **95% Confidence Interval (CI)** | **p-value** |
| --- | --- | --- | --- | --- | --- | --- |
| **Overall accuracy** | Patient Questionnaire | Expert Questionnaire | 214400 | -3.84E-06 | -2.985e-06 – -4.3314e-05 | 6.04e-18 |
| **Overall practicality** | Patient Questionnaire | Expert Questionnaire | 206300 | -5.81E-06 | -6.1313e-05 – -4.4795e-05 | 1.52e-14 |
| **Overall GSS** | Patient Questionnaire | Expert Questionnaire | 203200 | -2.0959e-05 | -3.9245e-05 - -2.8128e-05 | 2.15e-09 |

Difference (J-I), the average difference between the two groups.
