# Supplementary material for: Large Language Models as a Consulting Hotline for Patients With Breast Cancer and Specialists in China: Cross-Sectional Questionnaire Study
Source: JMIR Med Inform. 2025 May 27;13:e66429. doi: 10.2196/66429 (PMC12133073; doi:10.2196/66429)
Supplement: Multimedia Appendix 5 [file medinform-v13-e66429-s005.docx]

**Supplementary table S3.** Multiple hypothesis testing (Dunn’s test) results for overall accuracy, practicality and Generalization-Specificity Score (GSS) of patient questionnaires among different models.

| **Comparison Groups** | **Accuracy: Statistic** | **Accuracy: Estimator** | **Accuracy: Adjusted p-value** | **Practicality: Statistic** | **Practicality: Estimator** | **Practicality: Adjusted p-value** | **GSS: Statistic** | **GSS: Estimator** | **GSS:**  **Adjusted p-value** |
| --- | --- | --- | --- | --- | --- | --- | --- | --- | --- |
| **ChatGPT-E vs. ChatGPT-C** | -5.9231 | -83.051 | 9.48E-09 | -6.5669 | -93.962 | 1.54E-10 | -3.4047 | -53.408 | 0.0020 |
| **ChatGPT-E vs. ERNIE Bot** | -9.3747 | -131.45 | 2.08E-20 | -10.31 | -147.52 | 1.91E-24 | -5.2535 | -82.408 | 4.48e-07 |
| **ChatGPT-C vs. ERNIE Bot** | -3.4516 | -48.397 | 0.0017 | -3.7428 | -53.554 | 0.0005 | -1.8488 | -29 | 0.1935 |
