# Supplementary material for: Large Language Models as a Consulting Hotline for Patients With Breast Cancer and Specialists in China: Cross-Sectional Questionnaire Study
Source: JMIR Med Inform. 2025 May 27;13:e66429. doi: 10.2196/66429 (PMC12133073; doi:10.2196/66429)
Supplement: Multimedia Appendix 6 [file medinform-v13-e66429-s006.docx]

**Supplementary table S4.** Multiple hypothesis testing (Dunn’s test) results for overall accuracy, practicality and Generalization-Specificity Score (GSS) of expert questionnaires among different models.

| **Comparison Groups** | **Accuracy: Statistic** | **Accuracy: Estimator** | **Accuracy: Adjusted p-value** | **Practicality: Statistic** | **Practicality: Estimator** | **Practicality: Adjusted p-value** | **GSS: Statistic** | **GSS: Estimator** | **GSS:**  **Adjusted p-value** |
| --- | --- | --- | --- | --- | --- | --- | --- | --- | --- |
| **ChatGPT-E vs. ChatGPT-C** | -7.242 | -110.09 | 1.33E-12 | -5.2946 | -66.305 | 3.58E-07 | -5.704 | -91.077 | 3.51e-08 |
| **ChatGPT-E vs. ERNIE Bot** | -7.5469 | -114.73 | 1.34E-13 | -6.4725 | -81.056 | 2.89E-10 | -6.404 | -102.25 | 4.54e-10 |
| **ChatGPT-C vs. ERNIE Bot** | -0.30496 | -4.6359 | 1 | -1.1779 | -14.751 | 0.7165 | -0.69999 | -11.177 | 1.0000 |
