# Supplementary material for: Large Language Models as a Consulting Hotline for Patients With Breast Cancer and Specialists in China: Cross-Sectional Questionnaire Study
Source: JMIR Med Inform. 2025 May 27;13:e66429. doi: 10.2196/66429 (PMC12133073; doi:10.2196/66429)
Supplement: Multimedia Appendix 7 [file medinform-v13-e66429-s007.docx]

**Supplementary table S5.** Results of multiple hypothesis tests (Dunn’s test) on the accuracy, practicality and Generalization-Specificity Score (GSS) of specific questions of patient questionnaires across different models.

| **Subgroup** | **Comparison Groups** | **Accuracy: Statistic** | **Accuracy: Estimator** | **Accuracy: Adjusted p-value** | **Practicality: Statistic** | **Practicality: Estimator** | **Practicality: Adjusted p-value** | **GSS: Statistic** | **GSS: Estimator** | **GSS:**  **Adjusted p-value** |
| --- | --- | --- | --- | --- | --- | --- | --- | --- | --- | --- |
| **Q1** | ChatGPT-E vs ChatGPT-C | -1.6485 | -5.4231 | 0.2977 | -0.44334 | -1.5 | 1 | -1.036 | -4.1538 | 0.9006 |
| **Q1** | ChatGPT-E vs ERNIE Bot | -2.0343 | -6.6923 | 0.1258 | -0.44334 | -1.5 | 1 | 0.14389 | 0.57692 | 1.0000 |
| **Q1** | ChatGPT-C vs ERNIE Bot | -0.38582 | -1.2692 | 1 | 0 | 0 | 1 | 1.1799 | 4.7308 | 0.7141 |
| **Q2** | ChatGPT-E vs ChatGPT-C | 0.14011 | 0.53846 | 1 | -2.4288 | -8.5385 | 0.0434 | 0.45539 | 1.8462 | 1.0000 |
| **Q2** | ChatGPT-E vs ERNIE Bot | -2.9023 | -11.154 | 0.0111 | -3.0853 | -10.846 | 0.0061 | -2.0492 | -8.3077 | 0.1213 |
| **Q2** | ChatGPT-C vs ERNIE Bot | -3.0424 | -11.692 | 0.007 | -0.65645 | -2.3077 | 1 | -2.5046 | -10.154 | 0.0368 |
| **Q3** | ChatGPT-E vs ChatGPT-C | -0.73569 | -2.5769 | 1 | -2.0972 | -7.5 | 0.1079 | -0.61249 | -2.3846 | 1.0000 |
| **Q3** | ChatGPT-E vs ERNIE Bot | -1.5702 | -5.5 | 0.3491 | -2.936 | -10.5 | 0.01 | -0.86934 | -3.3846 | 1.0000 |
| **Q3** | ChatGPT-C vs ERNIE Bot | -0.83451 | -2.9231 | 1 | -0.83887 | -3 | 1 | -0.25685 | -1 | 1.0000 |
| **Q4** | ChatGPT-E vs ChatGPT-C | -2.8938 | -11.538 | 0.0114 | -2.4429 | -8.8846 | 0.0437 | -0.6633 | -2.7308 | 1.0000 |
| **Q4** | ChatGPT-E vs ERNIE Bot | -2.8938 | -11.538 | 0.0114 | -2.7919 | -10.154 | 0.0157 | -0.85014 | -3.5 | 1.0000 |
| **Q4** | ChatGPT-C vs ERNIE Bot | 0 | 0 | 1 | -0.34899 | -1.2692 | 1 | -0.18684 | -0.76923 | 1.0000 |
| **Q5** | ChatGPT-E vs ChatGPT-C | -1.556 | -6.0385 | 0.3591 | -1.1714 | -4.5 | 0.7243 | -0.67397 | -2.6923 | 1.0000 |
| **Q5** | ChatGPT-E vs ERNIE Bot | -3.439 | -13.346 | 0.0018 | -2.6132 | -10.038 | 0.0269 | -1.6079 | -6.4231 | 0.3236 |
| **Q5** | ChatGPT-C vs ERNIE Bot | -1.883 | -7.3077 | 0.1791 | -1.4417 | -5.5385 | 0.4481 | -0.93393 | -3.7308 | 1.0000 |
| **Q6** | ChatGPT-E vs ChatGPT-C | -2.9357 | -11.731 | 0.01 | -2.3961 | -8.9231 | 0.0437 | -2.3804 | -10 | 0.0519 |
| **Q6** | ChatGPT-E vs ERNIE Bot | -3.2148 | -12.846 | 0.0039 | -2.7162 | -10.115 | 0.0198 | -2.8382 | -11.923 | 0.0136 |
| **Q6** | ChatGPT-C vs ERNIE Bot | -0.27913 | -1.1154 | 1 | -0.32017 | -1.1923 | 1 | -0.45777 | -1.9231 | 1.0000 |
| **Q7** | ChatGPT-E vs ChatGPT-C | -0.02743 | -0.076923 | 1 | -1.0435 | -2.9231 | 0.8901 | 0.90406 | 3.5769 | 1.0000 |
| **Q7** | ChatGPT-E vs ERNIE Bot | -0.50745 | -1.4231 | 1 | -1.5104 | -4.2308 | 0.3928 | -0.17498 | -0.69231 | 1.0000 |
| **Q7** | ChatGPT-C vs ERNIE Bot | -0.48002 | -1.3462 | 1 | -0.46685 | -1.3077 | 1 | -1.079 | -4.2692 | 0.8417 |
| **Q8** | ChatGPT-E vs ChatGPT-C | -0.67452 | -2.5 | 1 | -2.4895 | -9.6923 | 0.0384 | -0.44588 | -1.8462 | 1.0000 |
| **Q8** | ChatGPT-E vs ERNIE Bot | -3.9641 | -14.692 | 0.0002 | -4.3269 | -16.846 | 4.54E-05 | -3.5113 | -14.538 | 0.0013 |
| **Q8** | ChatGPT-C vs ERNIE Bot | -3.2896 | -12.192 | 0.003 | -1.8375 | -7.1538 | 0.1984 | -3.0654 | -12.692 | 0.0065 |
| **Q9** | ChatGPT-E vs ChatGPT-C | -0.56335 | -1.4615 | 1 | -0.39791 | -1.5 | 1 | 0.16691 | 0.65385 | 1.0000 |
| **Q9** | ChatGPT-E vs ERNIE Bot | -1.0822 | -2.8077 | 0.8375 | -0.79582 | -3 | 1 | -0.57927 | -2.2692 | 1.0000 |
| **Q9** | ChatGPT-C vs ERNIE Bot | -0.51887 | -1.3462 | 1 | -0.39791 | -1.5 | 1 | -0.74617 | -2.9231 | 1.0000 |
| **Q10** | ChatGPT-E vs ChatGPT-C | -2.2617 | -8.6154 | 0.0711 | -1.1193 | -4.3846 | 0.7891 | -2.9267 | -12.192 | 0.0103 |
| **Q10** | ChatGPT-E vs ERNIE Bot | -2.5848 | -9.8462 | 0.0292 | -3.9174 | -15.346 | 0.0003 | -2.2805 | -9.5 | 0.0677 |
| **Q10** | ChatGPT-C vs ERNIE Bot | -0.3231 | -1.2308 | 1 | -2.7982 | -10.962 | 0.0154 | 0.64628 | 2.6923 | 1.0000 |
| **Q11** | ChatGPT-E vs ChatGPT-C | -2.6951 | -9.5 | 0.0211 | -1.6135 | -6.3846 | 0.3199 | -1.5592 | -6.2692 | 0.3568 |
| **Q11** | ChatGPT-E vs ERNIE Bot | -3.4261 | -12.077 | 0.0018 | -3.1395 | -12.423 | 0.0051 | -0.59309 | -2.3846 | 1.0000 |
| **Q11** | ChatGPT-C vs ERNIE Bot | -0.73106 | -2.5769 | 1 | -1.526 | -6.0385 | 0.381 | 0.96616 | 3.8846 | 1.0000 |
| **Q12** | ChatGPT-E vs ChatGPT-C | -2.2911 | -9.2308 | 0.0659 | -1.8071 | -7.1154 | 0.2123 | -1.1049 | -4.6154 | 0.8076 |
| **Q12** | ChatGPT-E vs ERNIE Bot | -2.8639 | -11.538 | 0.0126 | -3.7021 | -14.577 | 0.0006 | -1.3811 | -5.7692 | 0.5017 |
| **Q12** | ChatGPT-C vs ERNIE Bot | -0.57277 | -2.3077 | 1 | -1.895 | -7.4615 | 0.1743 | -0.27622 | -1.1538 | 1.0000 |
| **Q13** | ChatGPT-E vs ChatGPT-C | 0.10699 | 0.38462 | 1 | -1.471 | -4.3846 | 0.4239 | -0.95372 | -3.7692 | 1.0000 |
| **Q13** | ChatGPT-E vs ERNIE Bot | -0.29957 | -1.0769 | 1 | -1.8968 | -5.6538 | 0.1736 | -1.849 | -7.3077 | 0.1934 |
| **Q13** | ChatGPT-C vs ERNIE Bot | -0.40656 | -1.4615 | 1 | -0.42582 | -1.2692 | 1 | -0.89533 | -3.5385 | 1.0000 |
| **Q14** | ChatGPT-E vs ChatGPT-C | -0.46685 | -1.3077 | 1 | -2.6981 | -9.9615 | 0.0209 | 0.51519 | 2.0385 | 1.0000 |
| **Q14** | ChatGPT-E vs ERNIE Bot | -1.5104 | -4.2308 | 0.3928 | -4.0523 | -14.962 | 0.0002 | -0.019441 | -0.076923 | 1.0000 |
| **Q14** | ChatGPT-C vs ERNIE Bot | -1.0435 | -2.9231 | 0.8901 | -1.3542 | -5 | 0.527 | -0.53463 | -2.1154 | 1.0000 |
| **Q15** | ChatGPT-E vs ChatGPT-C | -3.1417 | -12.192 | 0.005 | -2.1429 | -8.3077 | 0.0964 | -2.1973 | -9.0769 | 0.0840 |
| **Q15** | ChatGPT-E vs ERNIE Bot | -3.1318 | -12.154 | 0.0052 | -2.5 | -9.6923 | 0.0373 | -1.5456 | -6.3846 | 0.3666 |
| **Q15** | ChatGPT-C vs ERNIE Bot | 0.0099107 | 0.038462 | 1 | -0.35714 | -1.3846 | 1 | 0.65174 | 2.6923 | 1.0000 |
