# Supplementary material for: Large Language Models as a Consulting Hotline for Patients With Breast Cancer and Specialists in China: Cross-Sectional Questionnaire Study
Source: JMIR Med Inform. 2025 May 27;13:e66429. doi: 10.2196/66429 (PMC12133073; doi:10.2196/66429)
Supplement: Multimedia Appendix 8 [file medinform-v13-e66429-s008.docx]

**Supplementary table S6.** Results of multiple hypothesis tests (Dunn’s test) on the accuracy, practicality and Generalization-Specificity Score (GSS) of specific questions of expert questionnaires across different models.

| **Subgroup** | **Comparison Groups** | **Accuracy: Statistic** | **Accuracy: Estimator** | **Accuracy: Adjusted p-value** | **Practicality: Statistic** | **Practicality: Estimator** | **Practicality: Adjusted p-value** | **GSS: Statistic** | **GSS: Estimator** | **GSS:**  **Adjusted p-value** |
| --- | --- | --- | --- | --- | --- | --- | --- | --- | --- | --- |
| **Q1** | ChatGPT-E vs ChatGPT-C | -0.72812 | -2.8462 | 1 | -1.1267 | -2.9231 | 0.7796 | 0.28338 | 1.1923 | 1.0000 |
| **Q1** | ChatGPT-E vs ERNIE Bot | -2.7846 | -10.885 | 0.0161 | -2.2089 | -5.7308 | 0.0815 | -1.5723 | -6.6154 | 0.3476 |
| **Q1** | ChatGPT-C vs ERNIE Bot | -2.0565 | -8.0385 | 0.1192 | -1.0822 | -2.8077 | 0.8375 | -1.8557 | -7.8077 | 0.1905 |
| **Q2** | ChatGPT-E vs ChatGPT-C | -1.0882 | -3.8462 | 0.8295 | -0.80672 | -2.6538 | 1 | -0.21848 | -0.88462 | 1.0000 |
| **Q2** | ChatGPT-E vs ERNIE Bot | -1.4582 | -5.1538 | 0.4344 | -1.6134 | -5.3077 | 0.3199 | -0.60793 | -2.4615 | 1.0000 |
| **Q2** | ChatGPT-C vs ERNIE Bot | -0.36999 | -1.3077 | 1 | -0.80672 | -2.6538 | 1 | -0.38946 | -1.5769 | 1.0000 |
| **Q3** | ChatGPT-E vs ChatGPT-C | -2.892 | -11.692 | 0.0115 | -1.0814 | -2.8077 | 0.8385 | -2.3635 | -9.8462 | 0.0543 |
| **Q3** | ChatGPT-E vs ERNIE Bot | -3.301 | -13.346 | 0.0029 | -1.6295 | -4.2308 | 0.3096 | -2.6498 | -11.038 | 0.0242 |
| **Q3** | ChatGPT-C vs ERNIE Bot | -0.40906 | -1.6538 | 1 | -0.54811 | -1.4231 | 1 | -0.28621 | -1.1923 | 1.0000 |
| **Q4** | ChatGPT-E vs ChatGPT-C | -0.73537 | -2.8462 | 1 | -1.0962 | -2.8462 | 0.8189 | 0 | 0 | 1.0000 |
| **Q4** | ChatGPT-E vs ERNIE Bot | -1.59 | -6.1538 | 0.3355 | -1.0814 | -2.8077 | 0.8385 | -0.38702 | -1.6154 | 1.0000 |
| **Q4** | ChatGPT-C vs ERNIE Bot | -0.85462 | -3.3077 | 1 | 0.014814 | 0.038462 | 1 | -0.38702 | -1.6154 | 1.0000 |
| **Q5** | ChatGPT-E vs ChatGPT-C | -3.7021 | -14.577 | 0.0006 | -1.5299 | -5.2308 | 0.3782 | -3.6094 | -14.423 | 0.0009 |
| **Q5** | ChatGPT-E vs ERNIE Bot | -2.9792 | -11.731 | 0.0087 | -2.6885 | -9.1923 | 0.0215 | -3.5805 | -14.308 | 0.0010 |
| **Q5** | ChatGPT-C vs ERNIE Bot | 0.72283 | 2.8462 | 1 | -1.1586 | -3.9615 | 0.7398 | 0.028875 | 0.11538 | 1.0000 |
| **Q6** | ChatGPT-E vs ChatGPT-C | -0.6205 | -2.3846 | 1 | 0.058557 | 0.19231 | 1 | 0.15805 | 0.65385 | 1.0000 |
| **Q6** | ChatGPT-E vs ERNIE Bot | -1.0008 | -3.8462 | 0.9508 | 0.43332 | 1.4231 | 1 | -0.63218 | -2.6154 | 1.0000 |
| **Q6** | ChatGPT-C vs ERNIE Bot | -0.3803 | -1.4615 | 1 | 0.37476 | 1.2308 | 1 | -0.79023 | -3.2692 | 1.0000 |
| **Q7** | ChatGPT-E vs ChatGPT-C | -2.4545 | -9.5 | 0.0423 | -1.4806 | -4.1538 | 0.4161 | -2.6202 | -10.885 | 0.0264 |
| **Q7** | ChatGPT-E vs ERNIE Bot | 0.36769 | 1.4231 | 1 | -1.4806 | -4.1538 | 0.4161 | -0.65736 | -2.7308 | 1.0000 |
| **Q7** | ChatGPT-C vs ERNIE Bot | 2.8222 | 10.923 | 0.0143 | 0 | 0 | 1 | 1.9628 | 8.1538 | 0.1490 |
| **Q8** | ChatGPT-E vs ChatGPT-C | -3.3052 | -13.077 | 0.0028 | -2.7721 | -10.077 | 0.0167 | -2.0694 | -8.2692 | 0.1155 |
| **Q8** | ChatGPT-E vs ERNIE Bot | -3.6649 | -14.5 | 0.0007 | -3.1318 | -11.385 | 0.0052 | -2.3485 | -9.3846 | 0.0565 |
| **Q8** | ChatGPT-C vs ERNIE Bot | -0.35968 | -1.4231 | 1 | -0.35973 | -1.3077 | 1 | -0.27913 | -1.1154 | 1.0000 |
| **Q9** | ChatGPT-E vs ChatGPT-C | -3.2733 | -12.577 | 0.0032 | -2.1932 | -7.7308 | 0.0849 | -2.1581 | -8.7308 | 0.0928 |
| **Q9** | ChatGPT-E vs ERNIE Bot | -3.6337 | -13.962 | 0.0008 | -2.9788 | -10.5 | 0.0087 | -2.8046 | -11.346 | 0.0151 |
| **Q9** | ChatGPT-C vs ERNIE Bot | -0.36036 | -1.3846 | 1 | -0.78561 | -2.7692 | 1 | -0.64649 | -2.6154 | 1.0000 |
| **Q10** | ChatGPT-E vs ChatGPT-C | -0.60021 | -2.3462 | 1 | -1.0962 | -2.8462 | 0.8189 | -1.3617 | -5.7308 | 0.5198 |
| **Q10** | ChatGPT-E vs ERNIE Bot | -0.72812 | -2.8462 | 1 | -1.0814 | -2.8077 | 0.8385 | -1.8735 | -7.8846 | 0.1830 |
| **Q10** | ChatGPT-C vs ERNIE Bot | -0.12791 | -0.5 | 1 | 0.014814 | 0.038462 | 1 | -0.5118 | -2.1538 | 1.0000 |
| **Q11** | ChatGPT-E vs ChatGPT-C | -3.2128 | -12.538 | 0.0039 | -2.9164 | -10.308 | 0.0106 | -2.3795 | -9.9615 | 0.0520 |
| **Q11** | ChatGPT-E vs ERNIE Bot | -2.996 | -11.692 | 0.0082 | -1.8173 | -6.4231 | 0.2075 | -1.7547 | -7.3462 | 0.2379 |
| **Q11** | ChatGPT-C vs ERNIE Bot | 0.21681 | 0.84615 | 1 | 1.0991 | 3.8846 | 0.8152 | 0.62473 | 2.6154 | 1.0000 |
| **Q12** | ChatGPT-E vs ChatGPT-C | -0.56335 | -1.4615 | 1 | -0.26726 | -1 | 1 | -0.55261 | -2.2308 | 1.0000 |
| **Q12** | ChatGPT-E vs ERNIE Bot | -1.0822 | -2.8077 | 0.8375 | -0.65787 | -2.4615 | 1 | -0.21914 | -0.88462 | 1.0000 |
| **Q12** | ChatGPT-C vs ERNIE Bot | -0.51887 | -1.3462 | 1 | -0.39061 | -1.4615 | 1 | 0.33347 | 1.3462 | 1.0000 |
| **Q13** | ChatGPT-E vs ChatGPT-C | -1.4675 | -4.1154 | 0.4267 | -0.37476 | -1.2308 | 1 | -2.049 | -8.2308 | 0.1214 |
| **Q13** | ChatGPT-E vs ERNIE Bot | -0.50745 | -1.4231 | 1 | -0.43332 | -1.4231 | 1 | -1.4554 | -5.8462 | 0.4367 |
| **Q13** | ChatGPT-C vs ERNIE Bot | 0.96005 | 2.6923 | 1 | -0.058557 | -0.19231 | 1 | 0.59364 | 2.3846 | 1.0000 |
| **Q14** | ChatGPT-E vs ChatGPT-C | -2.3932 | -9.4231 | 0.0501 | -0.43802 | -1.3077 | 1 | -1.4261 | -5.7692 | 0.4615 |
| **Q14** | ChatGPT-E vs ERNIE Bot | -2.5299 | -9.9615 | 0.0342 | -0.91469 | -2.7308 | 1 | -2.7381 | -11.077 | 0.0185 |
| **Q14** | ChatGPT-C vs ERNIE Bot | -0.13675 | -0.53846 | 1 | -0.47667 | -1.4231 | 1 | -1.312 | -5.3077 | 0.5686 |
| **Q15** | ChatGPT-E vs ChatGPT-C | -2.5548 | -9.8846 | 0.0319 | -2.5 | -9.6923 | 0.0373 | -2.6935 | -11.077 | 0.0212 |
| **Q15** | ChatGPT-E vs ERNIE Bot | -2.8431 | -11 | 0.0134 | -3.125 | -12.115 | 0.0053 | -2.5251 | -10.385 | 0.0347 |
| **Q15** | ChatGPT-C vs ERNIE Bot | -0.28828 | -1.1154 | 1 | -0.625 | -2.4231 | 1 | 0.16834 | 0.69231 | 1.0000 |
